# Supplementary material for: Targeting non-canonical pathways as a strategy to modulate the sodium iodide symporter
Source: Cell Chem Biol. 2022 Mar 17;29(3):502–516.e7. doi: 10.1016/j.chembiol.2021.07.016 (PMC8958605; doi:10.1016/j.chembiol.2021.07.016)
Supplement: Document S1. Figures S1–S7 and Tables S1–S7 [file mmc1.pdf]

Cell Chemical Biology, Volume *29*

Supplemental information

Targeting non-canonical pathways as a strategy to modulate the sodium iodide symporter

Martin L. Read, Katie Brookes, Caitlin E.M. Thornton, Alice Fletcher, Hannah R. Nieto, Mohammed Alshahrani, Rashida Khan, Patricia Borges de Souza, Ling Zha, Jamie R.M. Webster, Luke J. Alderwick, Moray J. Campbell, Kristien Boelaert, Vicki E. Smith, and Christopher J. McCabe

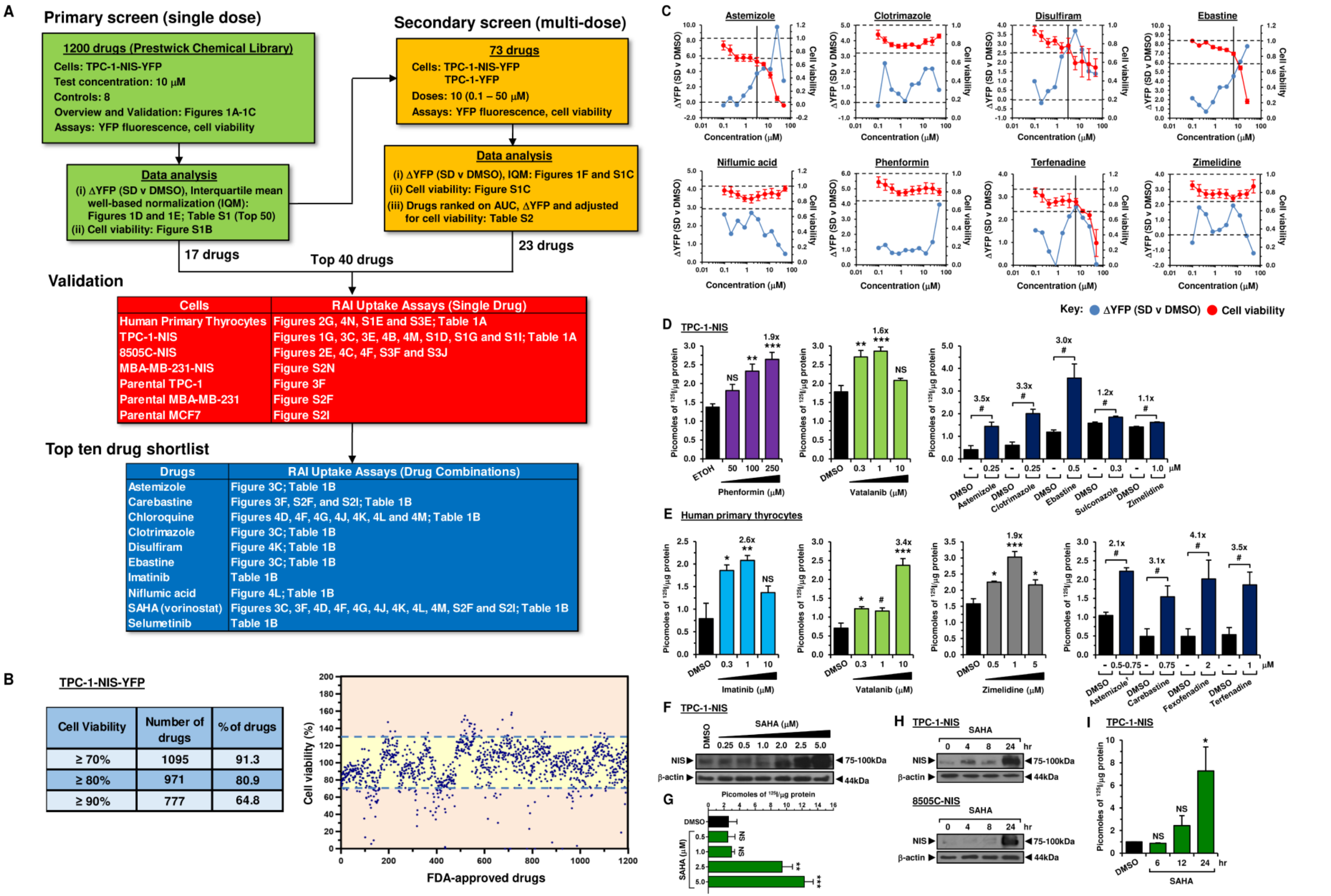

A
Primary screen (single dose)
1200 drugs (Prestwick Chemical Library)
Cells: TPC-1-NIS-YFP
Test concentration: 10 μM
Controls: 8
Overview and Validation: Figures 1A-1C
Assays: YFP fluorescence, cell viability
Data analysis
(i) ΔYFP (SD v DMSO), Interquartile mean well-based normalization (IQM): Figures 1D and 1E; Table S1 (Top 50)
(ii) Cell viability: Figure S1B
Secondary screen (multi-dose)
73 drugs
Cells: TPC-1-NIS-YFP
TPC-1-YFP
Doses: 10 (0.1 – 50 μM)
Assays: YFP fluorescence, cell viability
Data analysis
(i) ΔYFP (SD v DMSO), IQM: Figures 1F and S1C
(ii) Cell viability: Figure S1C
(iii) Drugs ranked on AUC, ΔYFP and adjusted for cell viability: Table S2
17 drugs
Top 40 drugs
23 drugs
Validation
Cells
RAI Uptake Assays (Single Drug)
Human Primary Thyrocytes
Figures 2G, 4N, S1E and S3E; Table 1A
TPC-1-NIS
Figures 1G, 3C, 3E, 4B, 4M, S1D, S1G and S1I; Table 1A
8505C-NIS
Figures 2E, 4C, 4F, S3F and S3J
MBA-MB-231-NIS
Figure S2N
Parental TPC-1
Figure 3F
Parental MBA-MB-231
Figure S2F
Parental MCF7
Figure S2I
Top ten drug shortlist
Drugs
RAI Uptake Assays (Drug Combinations)
Astemizole
Figure 3C; Table 1B
Carebastine
Figures 3F, S2F, and S2I; Table 1B
Chloroquine
Figures 4D, 4F, 4G, 4J, 4K, 4L and 4M; Table 1B
Clotrimazole
Figure 3C; Table 1B
Disulfiram
Figure 4K; Table 1B
Ebastine
Figure 3C; Table 1B
Imatinib
Table 1B
Niflumic acid
Figure 4L; Table 1B
SAHA (vorinostat)
Figures 3C, 3F, 4D, 4F, 4G, 4J, 4K, 4L, 4M, S2F and S2I; Table 1B
Selumetinib
Table 1B
B
TPC-1-NIS-YFP
Cell Viability | Number of drugs | % of drugs
≥ 70% | 1095 | 91.3
≥ 80% | 971 | 80.9
≥ 90% | 777 | 64.8
Cell viability (%)
FDA-approved drugs
C
Astemizole
Clotrimazole
Disulfiram
Ebastine
Niflumic acid
Phenformin
Terfenadine
Zimelidine
ΔYFP (SD v DMSO)
Cell viability
Concentration (μM)
Key: ΔYFP (SD v DMSO) Cell viability
D
TPC-1-NIS
Picomoles of 125I/μg protein
ETOH
Phenformin (μM)
Vatalanib (μM)
DMSO
Astemizole
Clotrimazole
Ebastine
Sulconazole
Zimelidine
3.5x
3.3x
3.0x
1.2x
1.1x
1.9x
1.6x
E
Human primary thyrocytes
Imatinib (μM)
Vatalanib (μM)
Zimelidine (μM)
Astemizole
Carebastine
Fexofenadine
Terfenadine
2.6x
3.4x
1.9x
2.1x
3.1x
4.1x
3.5x
F
TPC-1-NIS
SAHA (μM)
DMSO
NIS
β-actin
75-100kDa
44kDa
G
Picomoles of 125I/μg protein
SAHA (μM)
H
TPC-1-NIS
SAHA
8505C-NIS
hr
I
TPC-1-NIS
SAHA
hr

Figure S1 (related to Fig. 1, 2G, 3C, 3D). Identification of FDA-Approved Drugs that Enhance NIS Function

(**A**) High throughput screen (HTS) based on a newly adapted YFP biosensor assay was used in the primary screen (green boxes) to determine the efficacy of 1200 drugs (Prestwick Chemical Library, single dose) to increase intracellular iodide in TPC1-NIS-YFP cells. A secondary multi-dose screen (orange boxes) was undertaken to validate 73 of these drugs at 10 different doses in two YFP-expressing cell lines (i.e., TPC-1-NIS-YFP and TPC1-YFP). From this, a total of 40 drugs were validated in radioiodide uptake assays (red box) in multiple cell types as listed. Finally, a shortlist of 10 drugs was generated (blue box) and different drug combinations evaluated in radioiodide uptake assays. (**B**) TPC-1-NIS-YFP cell viability following treatment with a 10 μM dose of 1200 drugs (Prestwick Chemical Library) for 24 hr. Each circle represents a mean value from 2 biological replicates. Table (left) summarizes number of drugs associated with TPC-1-NIS-YFP cellular viability equal or greater than 70, 80 or 90%. Cellular viability determined using the alamarBlue (resazurin) assay. (**C**) YFP-based biosensing and cell viability of TPC-1-NIS-YFP cells treated for 24 hr at 10 different drug doses (0.1 – 50 μM). 8 out of 73 representative drug profiles are shown. Blue circles represent mean ΔYFP values from 2 biological replicates. Cell viability (red circles) was determined using the alamarBlue (resazurin) assay (Mean ± S.E.M., n = 4). Pharmacologic parameters (e.g., AUC) were determined up to maximum drug dose associated with cell viability > 70% (indicated by vertical black line). (**D, E**) Radioiodide uptake of TPC-1-NIS cells (D) and primary human thyrocytes (E) treated with the indicated drug for 24 hr. Data for astemizole[†] is compiled from human primary thyrocytes treated with two different doses (0.5 and 0.75 μM). (**F**) Western blot analysis of NIS expression in TPC-1-NIS cells treated with SAHA at indicated doses for 24 hr. (**G**) Radioiodide uptake of TPC-1-NIS cells treated with SAHA at indicated doses for 24 hr.(**H**) Western blot analyses of NIS protein levels in TPC-1-NIS (upper) and 8505C-NIS cells (lower) at indicated times post-treatment (hr) with 2.5 μM SAHA. (**I**) Same as (D) but radioiodide uptake in TPC-1-NIS cells at indicated times post-treatment (hr) with 2.5 μM SAHA. Data presented as mean ± S.E.M., n = 3, one-way ANOVA followed by Dunnett's post hoc test (NS, not significant; *p < 0.05; **p < 0.01; ***p < 0.001) or unpaired two-tailed *t*-test ($^{\#}p < 0.05$).

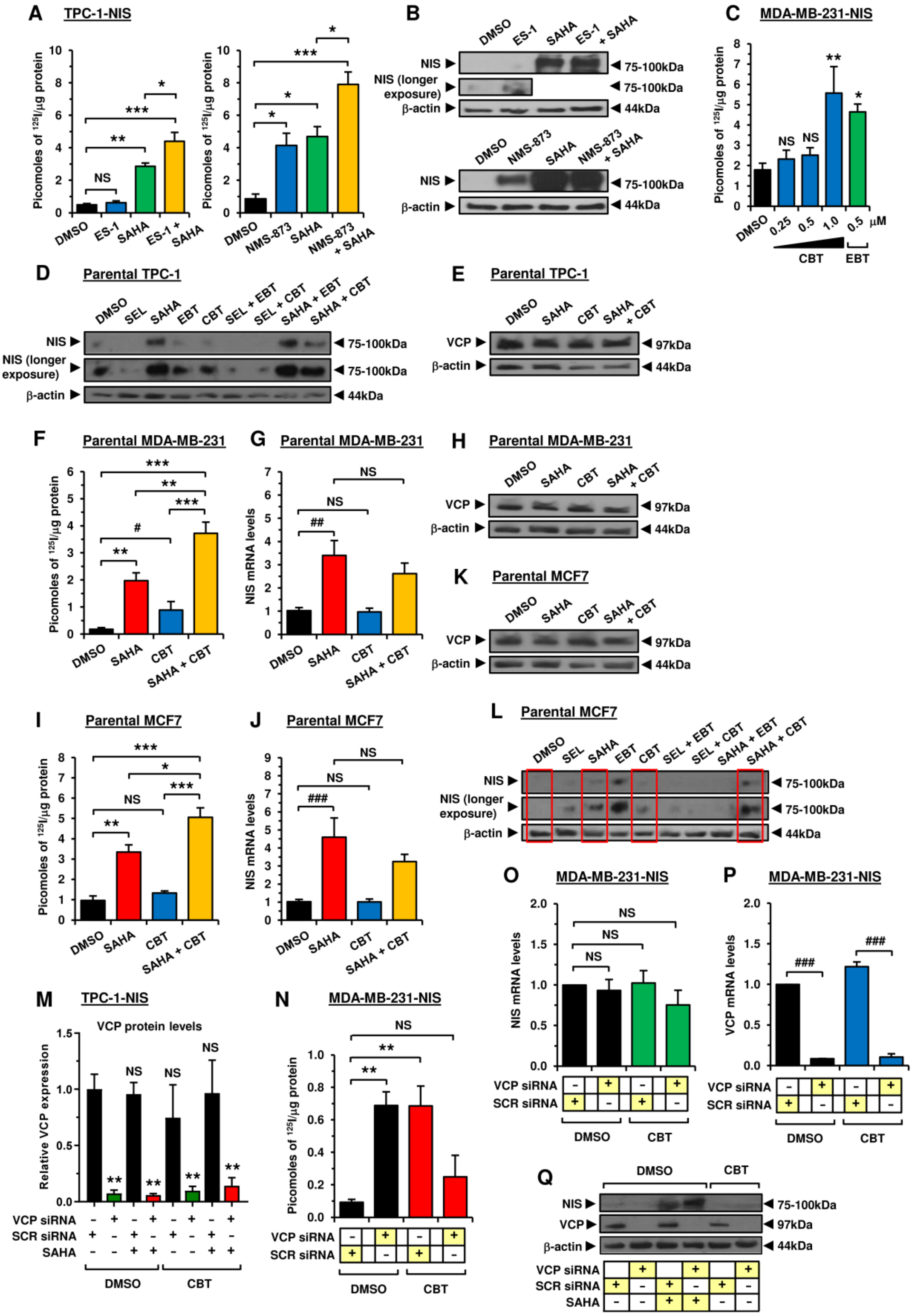

A TPC-1-NIS
Picomoles of ^{125}I/μg protein
DMSO
ES-1
SAHA
ES-1 + SAHA
NMS-873
NMS-873 + SAHA
B
NIS
NIS (longer exposure)
β-actin
75-100kDa
44kDa
C MDA-MB-231-NIS
CBT
EBT
μM
D Parental TPC-1
SEL
EBT
CBT
SEL + EBT
SEL + CBT
SAHA + EBT
SAHA + CBT
E Parental TPC-1
VCP
97kDa
F Parental MDA-MB-231
G Parental MDA-MB-231
NIS mRNA levels
H Parental MDA-MB-231
K Parental MCF7
I Parental MCF7
J Parental MCF7
L Parental MCF7
O MDA-MB-231-NIS
P MDA-MB-231-NIS
VCP mRNA levels
VCP siRNA
SCR siRNA
M TPC-1-NIS
VCP protein levels
Relative VCP expression
N MDA-MB-231-NIS
Q

Figure S2 (related to Fig. 3). Combined VCP Inhibitor and SAHA Treatment Augments Radioiodide Uptake in Thyroid and Breast Cancer Cells

(**A, B**) Radioiodide uptake (A) and relative NIS expression (B) in TPC-1-NIS cells treated with 2.5 μM eeyarestatin-1 (ES-1) or 5.0 μM NMS-873 either alone or in combination with 2.5 μM SAHA. SAHA was added 12 hr prior to ES-1 or NMS-873. (**C**) Radioiodide uptake of MDA-MB-231-NIS cells treated with carebastine (CBT) at indicated doses for 24 hr. Controls: DMSO and 0.5 μM ebastine (EBT). (**D**) Relative NIS protein expression in parental TPC-1 cells treated with 0.2 μM selumetinib (SEL), 2.5 μM SAHA, 0.5 μM EBT and 1.0 μM CBT, either alone or in combination as indicated. SEL treatment included for comparison. (**E**) Relative VCP expression in parental TPC-1 cells treated with 2.5 μM SAHA and 1.0 μM CBT either alone or in combination. (**F, G**) Radioiodide uptake (F) and relative NIS mRNA levels (G) in parental MDA-MB-231 cells treated with 2.5 μM SAHA and 1.0 μM CBT either alone or in combination. (**H**) Same as (E) but with parental MDA-MB-231 cells. (**I-K**) Same as (F-H) but with parental MCF7 cells. (**L**) Same as (D) but with parental MCF7 cells. Red boxes highlight induction of NIS protein by SAHA and SAHA+CBT compared to DMSO or CBT treatment alone. (**M**) Relative VCP protein levels in TPC-1-NIS cells following VCP-siRNA depletion and treatment with 1.0 μM CBT and/or 2.5 μM SAHA. (**N, O**) Radioiodide uptake (N) and relative NIS mRNA levels (O) in MDA-MB-231-NIS cells following VCP-siRNA depletion and treatment with 1.0 μM CBT. (**P, Q**) Relative VCP mRNA (P) and protein levels (Q), as well as NIS expression (Q), in MDA-MB-231-NIS cells following VCP-siRNA depletion and treatment with 1.0 μM CBT and/or 2.5 μM SAHA. Data presented as mean ± S.E.M., n = 3-6, one-way ANOVA followed by Tukey's (A, F, I) or Dunnett's (C, M, N, O) post hoc test (NS, not significant; *p < 0.05; **p < 0.01; ***p < 0.001) or unpaired two-tailed *t*-test ($^{\#}$p < 0.05; $^{\#\#}$p < 0.01; $^{\#\#\#}$p < 0.001).

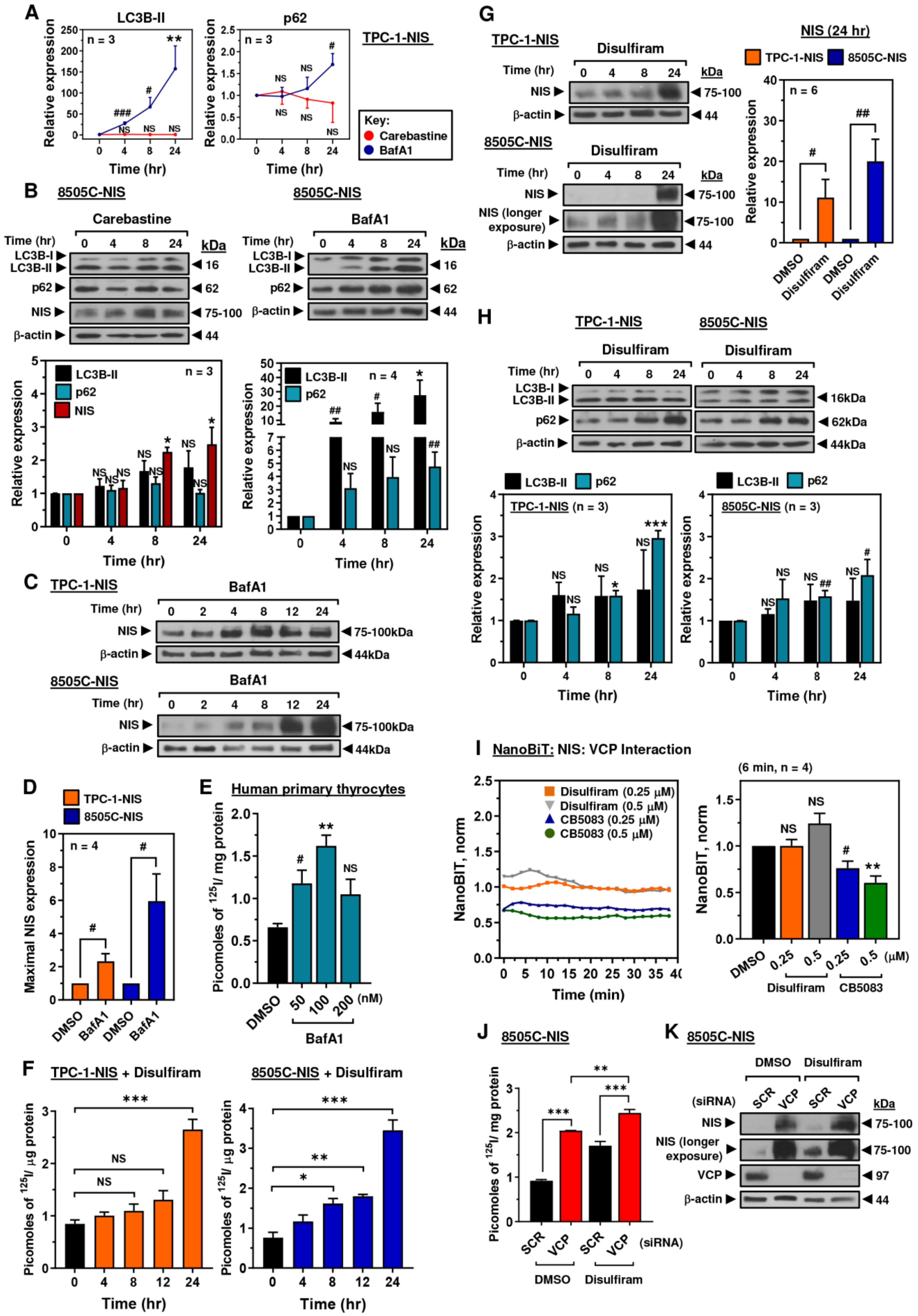
A
LC3B-II
p62
TPC-1-NIS
n = 3
Relative expression
Time (hr)
Key:
Carebastine
BafA1
B
8505C-NIS
Carebastine
BafA1
Time (hr)
kDa
LC3B-I
LC3B-II
p62
NIS
β-actin
16
62
75-100
44
Relative expression
n = 4
C
TPC-1-NIS
8505C-NIS
75-100kDa
44kDa
D
Maximal NIS expression
DMSO
BafA1
E
Human primary thyrocytes
Picomoles of 125I/ mg protein
(nM)
F
TPC-1-NIS + Disulfiram
8505C-NIS + Disulfiram
Picomoles of 125I/ µg protein
G
Disulfiram
NIS (longer exposure)
NIS (24 hr)
n = 6
H
16kDa
62kDa
I
NanoBiT: NIS: VCP Interaction
Disulfiram (0.25 µM)
Disulfiram (0.5 µM)
CB5083 (0.25 µM)
CB5083 (0.5 µM)
NanoBIT, norm
Time (min)
(6 min, n = 4)
(µM)
CB5083
J
SCR
VCP
(siRNA)
DMSO
Disulfiram
K
VCP
97

Figure S3 (related to Fig. 3L, 4A-E). Autophagy Inhibitor BafA1 Augments NIS Expression and Radioiodide Uptake in Thyroid Cells

(**A**) Scanning densitometry performed relative to β-actin on Western blot analysis of LC3B-II (left) and p62 protein levels (right) in TPC-1-NIS cells treated with 1.0 μM carebastine (CBT, red spots) or 100 nM bafilomycin A1 (bafA1, blue spots) for the indicated timecourse (hr). See also Figure 3L. (**B**) Western blot analysis of LC3B-I, LC3B-II and p62 protein levels in 8505C-NIS cells treated with 1.0 μM CBT (left) or 100 nM bafA1 (right) for the indicated timecourse (hr). Scanning densitometry (below) performed relative to β-actin. Relative NIS expression levels are also shown for CBT treated 8505-NIS cells. (**C-D**) Western blot analysis of NIS protein levels in TPC-1-NIS and 8505C-NIS cells treated with 100 nM bafA1 for the indicated timecourse (hr). Scanning densitometry performed relative to β-actin (D). (**E**) Radioiodide uptake of primary human thyrocytes treated with bafA1 at the indicated dose for 24 hr. (**F**) Radioiodide uptake of TPC-1-NIS and 8505C-NIS cells treated with 0.5 μM disulfiram for the indicated timecourse. (**G**) Relative NIS protein levels in TPC-1-NIS and 8505C-NIS cells treated with 0.5 μM disulfiram for the indicated timecourse (hr). Scanning densitometry (right) performed relative to β-actin (n = 6). (**H**) Western blot analysis of LC3B-I, LC3B-II and p62 protein levels in TPC-1-NIS and 8505C-NIS cells treated with 0.5 μM disulfiram for the indicated timecourse (hr). Scanning densitometry (below) performed relative to β-actin (n = 3). (**I**) Evaluation of VCP binding to NIS using NanoBiT in HeLa cells treated with disulfiram or VCP inhibitor CB5083 for 24 hr at the indicated dose (left, n = 4). Normalised NanoBiT assay results shown at 6 min after addition of Nano-Glo live cell assay solution (right). (**J**) Radioiodide uptake of 8505C-NIS cells following VCP-siRNA depletion and treatment with 0.5 μM disulfiram. (**K**) Relative NIS and VCP protein levels in 8505C-NIS cells following VCP-siRNA depletion and treatment with 0.5 μM disulfiram. Data presented as mean ± S.E.M., one-way ANOVA followed by Dunnett's post hoc test (NS, not significant; *p < 0.05; **p < 0.01; ***p < 0.001) or unpaired two-tailed *t*-test ($^{\#}$p < 0.05; $^{\#\#}$p < 0.01; $^{\#\#\#}$p < 0.01).

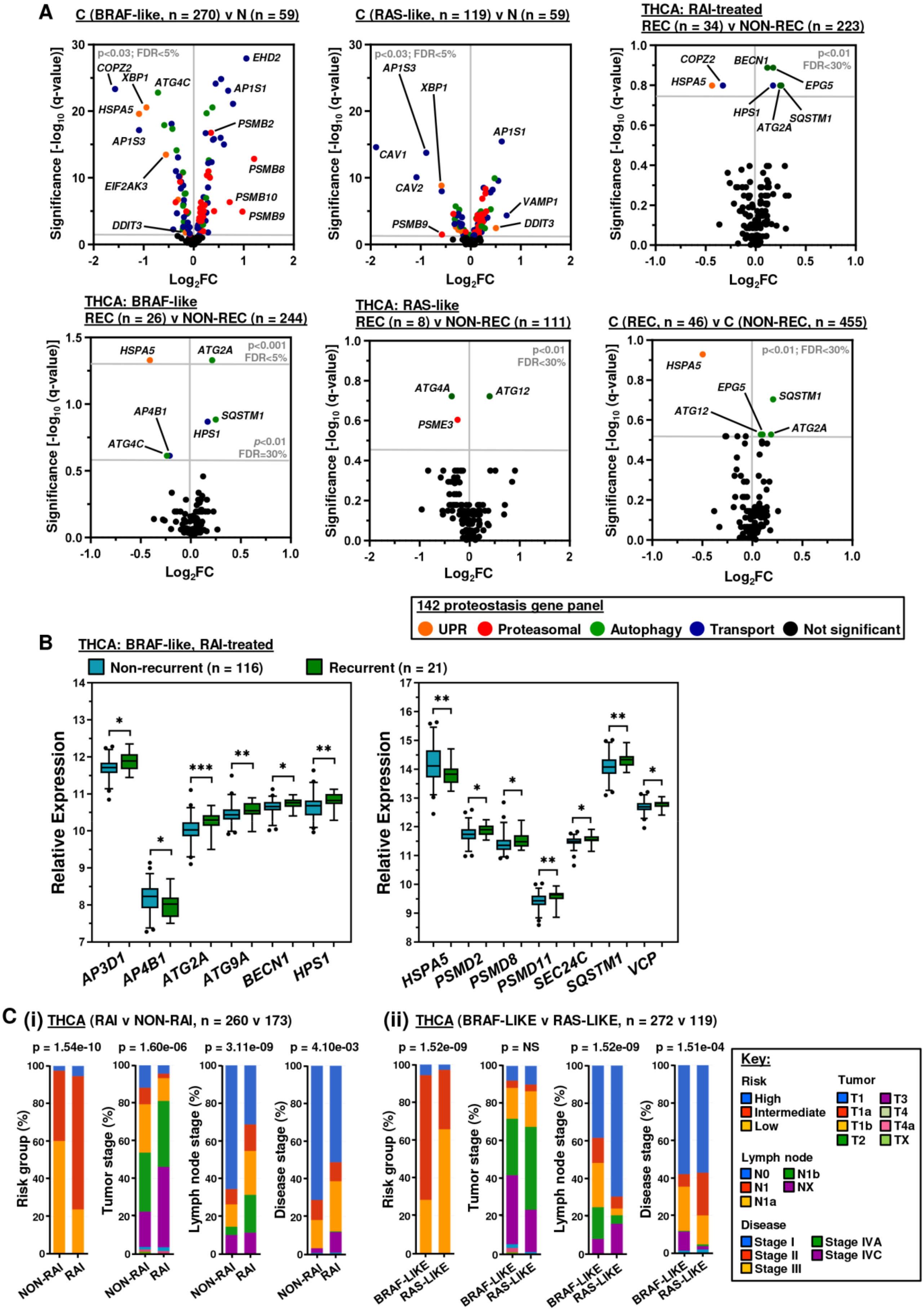
A
C (BRAF-like, n = 270) v N (n = 59)
C (RAS-like, n = 119) v N (n = 59)
THCA: RAI-treated REC (n = 34) v NON-REC (n = 223)
THCA: BRAF-like REC (n = 26) v NON-REC (n = 244)
THCA: RAS-like REC (n = 8) v NON-REC (n = 111)
C (REC, n = 46) v C (NON-REC, n = 455)
Significance [-log10 (q-value)]
Log2FC
142 proteostasis gene panel
UPR
Proteasomal
Autophagy
Transport
Not significant
B
THCA: BRAF-like, RAI-treated
Non-recurrent (n = 116)
Recurrent (n = 21)
Relative Expression
C (i) THCA (RAI v NON-RAI, n = 260 v 173)
(ii) THCA (BRAF-LIKE v RAS-LIKE, n = 272 v 119)
Risk group (%)
Tumor stage (%)
Lymph node stage (%)
Disease stage (%)
Key:
Risk
High
Intermediate
Low
Tumor
Lymph node
Disease

Figure S4 (related to Fig. 5A-C). Identification of Proteostasis Modulators Associated with Cancer Genetic Signature, Recurrence and Radioiodide Therapy

(**A**) (upper) Volcano plots comparing log_2FC with q-value (-log base 10) for 142 core proteostasis genes in the BRAF-like (left) or RAS-like (middle) THCA cohort (C) versus normal (N). (upper right) Volcano plot illustrating log_2FC with q-value (-log base 10) for 142 core proteostasis genes in the RAI-treated THCA cohort [recurrent (REC) versus non-recurrent (NON-REC)]. (lower) Volcano plots comparing log_2FC with q-value (-log base 10) for 142 core proteostasis genes in the BRAF-like (left), RAS-like (middle) or entire (right) THCA cohort [REC versus NON-REC]. Gene categories indicated by coloured spots. (**B**) Box and whisker plots showing expression (log_2) of 13 core proteostasis genes in the BRAF-like, RAI-treated THCA cohort [recurrent (REC) versus non-recurrent (NON-REC)]; P-values determined by Mann-Whitney test and adjusted using the Benjamini-Hochberg FDR correction procedure (NS, not significant; $*p < 0.05$; $**p < 0.01$; $***p < 0.001$). (**C**) Stacked bar charts showing clinical characteristics of (i) RAI (n = 260) versus non-RAI (n = 173) treated THCA patients or (ii) THCA patients stratified by BRAF-like (n = 272) or RAS-like (n = 119) tumoral genetic signatures. Clinical staging attributes include risk group, tumor staging, lymph node staging and disease staging. P-values derived using Chi-Squared test and adjusted using the Benjamini-Hochberg FDR correction procedure. NS, not significant.

A

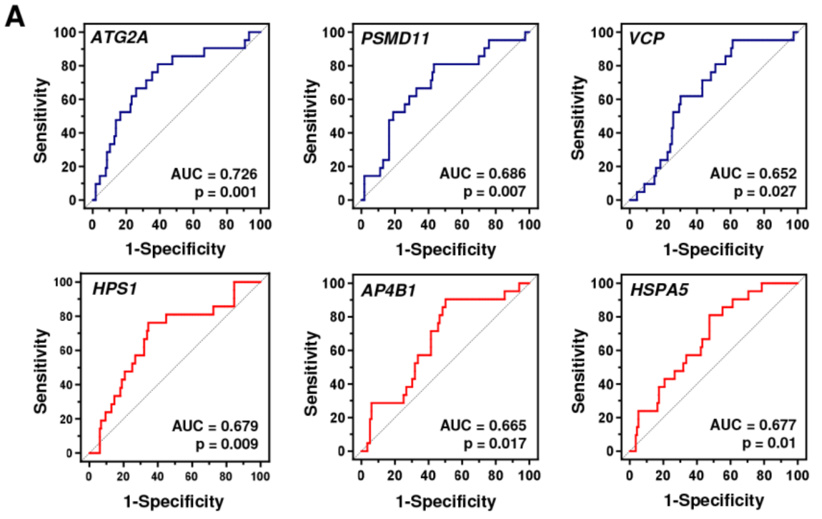

B

Gene	Sensitivity (%)	Specificity (%)	Cut-off value
AP3D1	52.38	81.03	11.89
AP4B1	90.48	50.00	8.23
ATG2A	80.95	61.21	10.10
ATG9A	76.19	59.48	10.48
BECN1	57.14	70.69	10.76
HPS1	76.19	65.52	10.75
HSPA5	80.95	52.59	14.04
PSMD2	76.19	55.17	11.76
PSMD8	57.14	69.83	11.47
PSMD11	80.95	56.90	9.48
SEC24	76.19	61.21	11.52
SQSTM1	80.95	51.72	14.15
VCP	61.90	69.83	12.77

C THCA (BRAF-like, RAI treated (n = 137): ROC analysis cut-off values

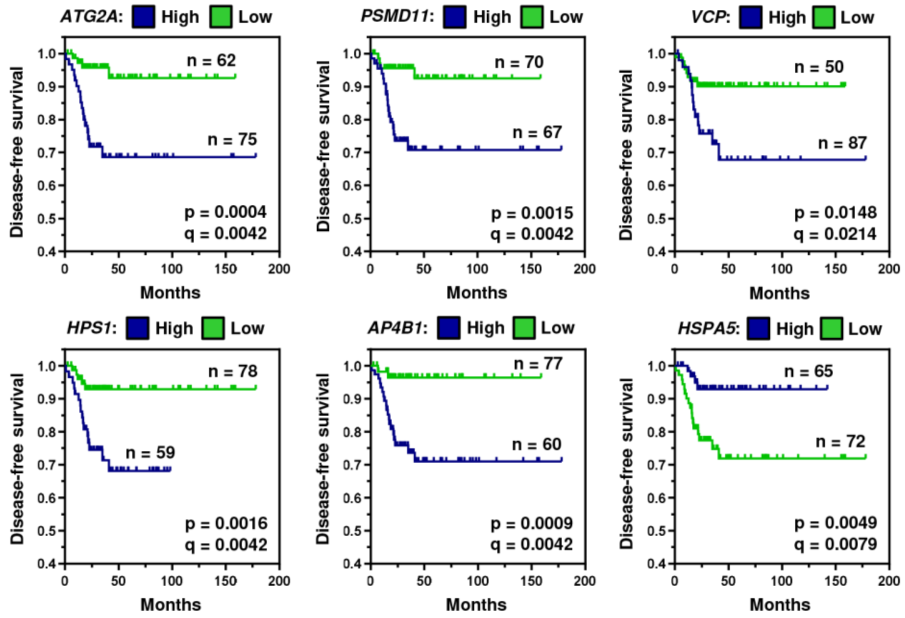

D THCA (BRAF-like, RAI-treated, n = 137): Percentile cut-off values

Gene	THCA: BRAF-like, RAI-treated (n = 137)																	
	HIGH (>66) v LOW (<33)			HIGH (>66) V LOW (<66)			HIGH (>33) V LOW (<33)			HIGH (>75) V LOW (<25)			HIGH (>50) V LOW (<50)			HIGH (>75) V LOW (<75)		
	p	q	NREC	p	q	NREC	p	q	NREC	p	q	NREC	p	q	NREC	p	q	NREC
AP3D1	0.0248	0.0465	12,4	0.0110	0.0300	12,9	0.1232	0.1719	17,4	0.0193	0.0380	11,3	0.1018	0.1103	14,7	0.0022	0.0141	11,10
AP4B1	0.0288	0.0465	2,9	0.0153	0.0300	2,19	0.3412	0.3412	12,9	0.1372	0.1372	2,6	0.0333	0.0481	6,15	0.7770	0.7770	2,19
ATG2A	0.0033	0.0216	14,3	0.0004	0.0049	14,7	0.0398	0.1719	18,3	0.0042	0.0350	11,2	0.0020	0.0174	17,4	0.0015	0.0141	11,10
ATG9A	0.0468	0.0554	11,4	0.0481	0.0625	11,10	0.1216	0.1719	17,4	0.0081	0.0350	9,1	0.0115	0.0331	16,5	0.0734	0.1061	9,12
BECN1	0.0322	0.0465	12,4	0.0115	0.0300	12,9	0.1455	0.1719	17,4	0.0526	0.0622	9,3	0.0303	0.0481	15,6	0.0376	0.0814	9,12
HPS1	0.0428	0.0554	12,4	0.0161	0.0300	12,9	0.1844	0.1997	17,4	0.0352	0.0572	10,3	0.0052	0.0224	17,4	0.0098	0.0317	10,11
HSPA5	0.0120	0.0390	2,10	0.0149	0.0300	2,19	0.1053	0.1719	11,10	0.0057	0.0350	1,9	0.0767	0.0906	7,14	0.0259	0.0673	1,20
PSMD2	0.0603	0.0653	11,4	0.0701	0.0759	11,10	0.1294	0.1719	17,4	0.0511	0.0622	9,3	0.0127	0.0331	16,5	0.0667	0.1061	9,12
PSMD8	0.0285	0.0465	11,3	0.0583	0.0689	11,10	0.0532	0.1719	18,3	0.0179	0.0380	8,1	0.1352	0.1352	14,7	0.1736	0.2052	8,13
PSMD11	0.0173	0.0451	13,4	0.0050	0.0300	13,8	0.1058	0.1719	17,4	0.0204	0.0380	11,3	0.0027	0.0174	17,4	0.0052	0.0224	11,10
SEC24C	0.1025	0.1025	10,4	0.1871	0.1871	10,11	0.1398	0.1719	17,4	0.1342	0.1372	9,4	0.0171	0.0335	16,5	0.0598	0.1061	9,12
SQSTM1	0.0108	0.0390	11,2	0.0439	0.0625	11,10	0.0209	0.1355	19,2	0.0181	0.0380	8,1	0.0180	0.0335	16,5	0.1566	0.2036	8,13
VCP	0.0018	0.0216	12,1	0.0260	0.0422	12,9	0.0037	0.0485	20,1	0.0415	0.0599	7,1	0.0679	0.0883	15,6	0.5690	0.6164	7,14
Genes (p < 0.05; q < 0.05)		9/13			8/13			1/13			7/13			9/13			4/13	

Figure S5 (related to Fig. 5D-F). A 13 Proteostasis Gene Panel is Associated with Recurrence in the BRAF-like, RAI-Treated THCA Cohort

(**A**) Representative ROC curves of 6 proteostasis genes in the BRAF-like, RAI-treated THCA cohort (n = 137). (**B**) Comparison of clinical sensitivity and specificity of 13 proteostasis genes derived by ROC analysis using optimal cut-off expression values (log_2 values) in the BRAF-like, RAI-treated THCA cohort (n = 137). (**C**) Representative Kaplan-Meier analysis of DFS for the BRAF-like, RAI-treated THCA cohort stratified on high versus low tumoral expression of indicated core proteostasis genes; log-rank test. Number (n) of patients per expression sub-group (high/low), p-values and q-values are shown. (**D**) Kaplan-Meier analysis of the BRAF-like, RAI-treated THCA cohort stratified on high versus low tumoral expression according to indicated percentile cut-off values; log-rank test. Significance indicated by p- and q-values. N_{REC} = number of recurrent cases in cohort with either high (left) or low (right) tumoral proteostasis expression. Green = FDR < 5%; Orange = greatest level of significance per percentile cut-off group; Yellow = highest percentage of stratified recurrent cases per percentile cut-off group.

A THCA (BRAF-like, RAI-treated, n = 137)

13 gene signature (continuous, multi-factor Cox)

Gene	Multivariate	
	Coefficient	p-value
AP3D1	0.000088	0.902
AP4B1	0.002892	0.584
ATG2A	-0.000932	0.447
ATG9A	0.001931	0.116
BECN1	0.002862	0.110
HSPA5	-0.000125	0.031
HPS1	0.000045	0.969
PSMD2	-0.001065	0.087
PSMD8	0.000185	0.687
PSMD11	0.002498	0.440
SEC24C	0.002052	0.201
SQSTM1	0.000036	0.653
VCP	0.000265	0.354

B THCA (BRAF-like, RAI-treated, n = 137)

Riskscore	Sensitivity (%)	Specificity (%)	HR (95% CI)	p-value
10.84	100	18.97	26.381 (0.164-4231.231)	NS
12.60	95.24	69.83	35.862 (4.81-267.405)	4.79×10^{-4}
12.73	90.48	71.55	17.794 (4.142-76.445)	1.09×10^{-4}
12.86	85.71	75.86	13.352 (3.930-45.358)	3.30×10^{-5}
13.03	80.95	80.17	11.978 (4.027-35.626)	8.0×10^{-6}
13.13	71.43	82.76	7.977 (3.090-20.589)	1.8×10^{-5}
13.16	66.67	82.76	6.980 (2.812-17.326)	2.8×10^{-5}
13.21	61.9	83.62	6.189 (2.562-14.951)	5.1×10^{-5}

C GSE27155: PTC (BRAF T1799A, n = 26) v N (n = 4)

D GSE27155: PTC (RAS mutation, n = 7) v N (n = 4)

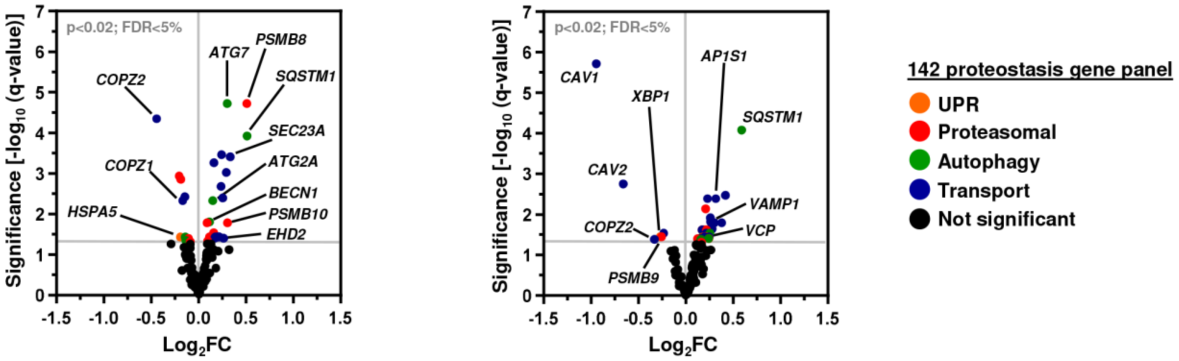

E

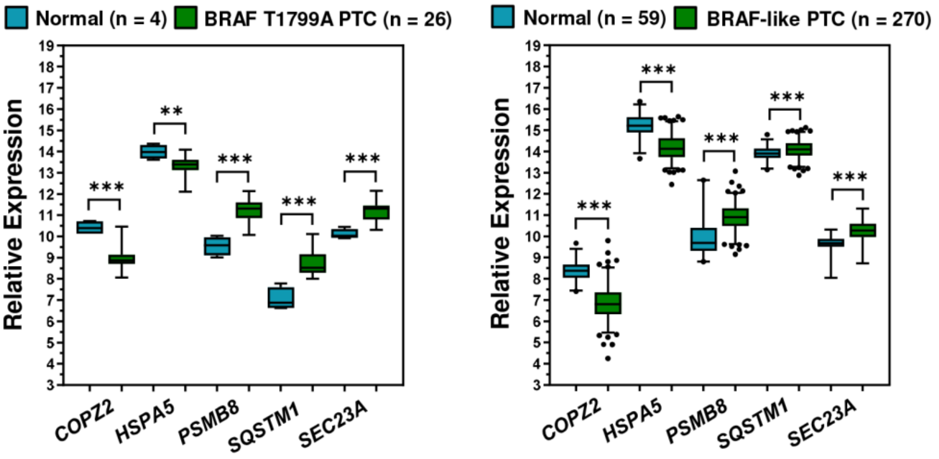

F GSE27155 v THCA

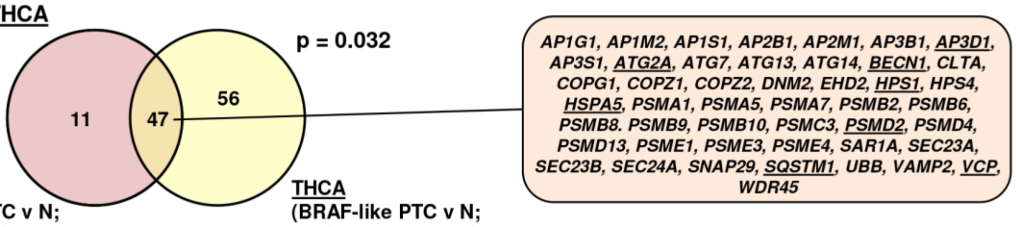

GSE27155 (BRAF T1799A PTC v N; 58/129 proteostasis genes; p < 0.05; FDR < 10%)

THCA (BRAF-like PTC v N; 102/142 proteostasis genes; p < 0.03; FDR < 5%)

Figure S6 (related to Fig. 5C, G, H, I). Profiling and Validation of the 13 Proteostasis Gene Riskscore Classifier

(**A**) Regression coefficients of 13 proteostasis genes in the BRAF-like, RAI-treated THCA cohort (multivariate Cox regression analysis). (**B**) Profile of riskscore cut-off values of the 13 gene proteostasis riskscore classifier along with estimations of sensitivity and specificity (%) for the BRAF-like, RAI-treated THCA cohort (n = 137). Hazard ratios (HR) ± 95% CI using riskscore cut-off values to stratify into high and low risk cohorts are shown (univariate Cox regression analysis). (**C, D**) Volcano plot comparing $\log_2$FC with q-value (-log base 10) for 142 core proteostasis genes in the GSE27155 dataset [BRAF T1799A PTC versus N (C) and RAS mutation PTC versus N (D)]. Gene categories indicated by coloured spots. (**E**) Representative box and whisker plots showing expression ($\log_2$) of 5 core proteostasis genes in the GSE27155 (BRAF T1799A PTC versus normal; left) and THCA (BRAF-like PTC versus normal; right) datasets; P-values determined by Mann-Whitney test and adjusted using the Benjamini-Hochberg FDR correction procedure (**p < 0.01; ***p < 0.001). (**F**) Venn diagram showing significant overlap in the 142 core proteostasis gene panel between the GSE27155 (BRAF T1799A PTC versus normal) and THCA (BRAF-like PTC versus normal) datasets. Genes in the 13 gene proteostasis riskscore classifier are underlined.

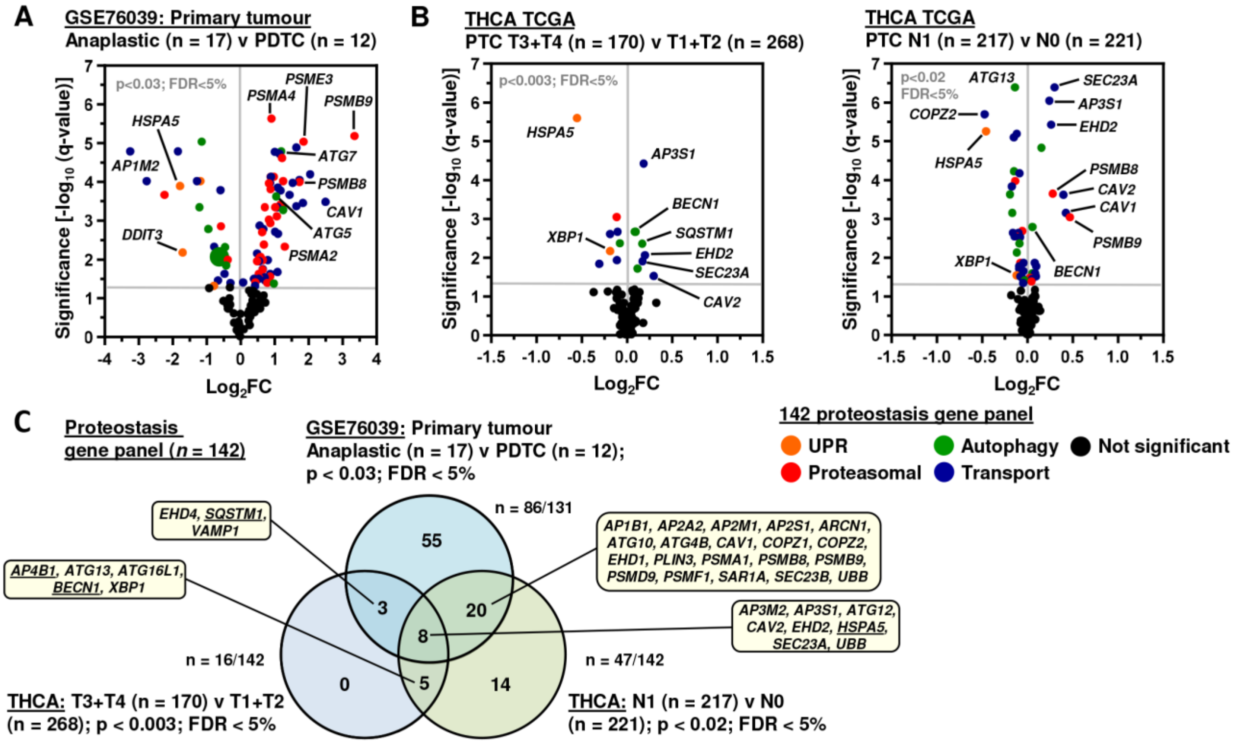

D

THCA (BRAF-like, RAI-treated, n = 137)

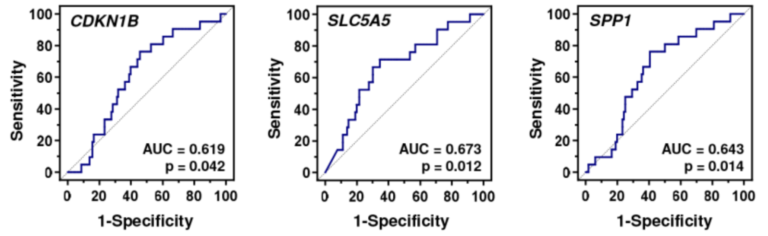

Gene	AUC	p-value	Cut-off value
CDKN1A	0.557	NS	12.89
CDKN1B	0.619	0.042	10.43
CCND1	0.527	NS	13.38
CDH1	0.538	NS	14.05
CEACAM1	0.638	0.021	5.52
SLC5A5	0.673	0.012	0.34
SPP1	0.643	0.014	9.61

E

THCA (BRAF-like, RAI-treated, n = 124)

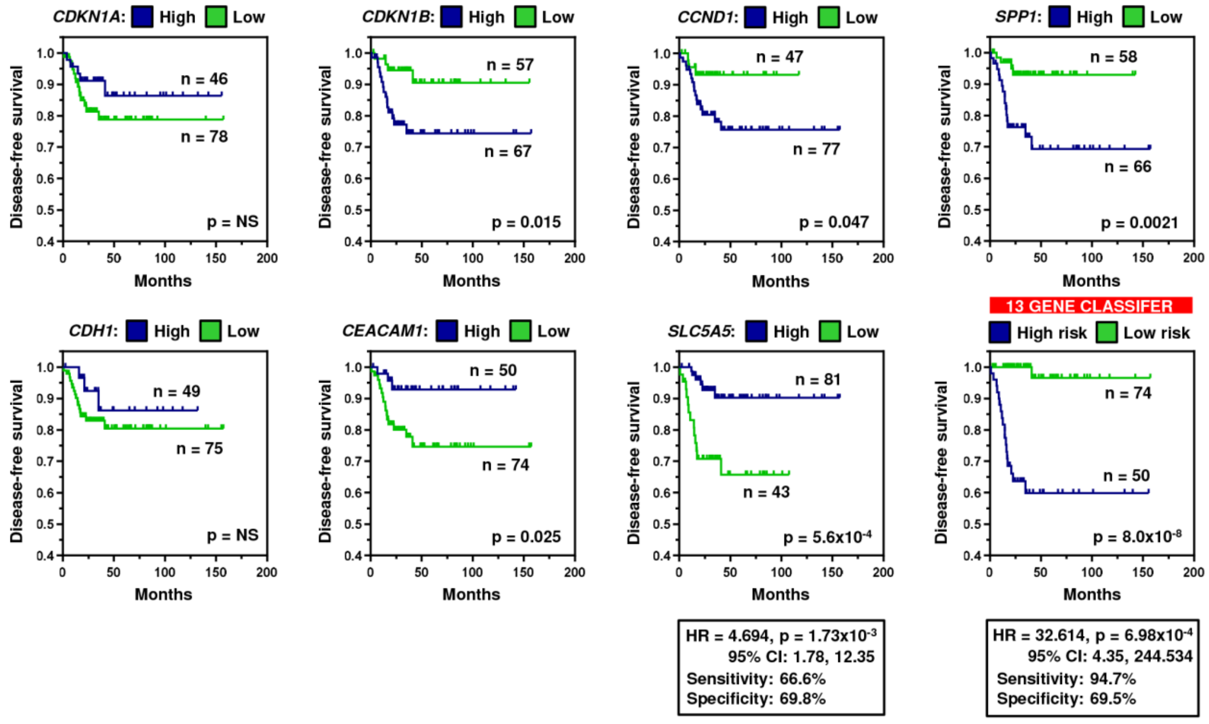

Figure S7 (related to Fig. 5). Greater Predictive Value of the 13-Gene Riskscore Classifier Compared to a Panel of Molecular Biomarkers

(**A**) Volcano plot comparing log_2FC with q-value (-log base 10) for 142 core proteostasis genes in the GSE276039 dataset [anaplastic thyroid cancer (n = 17) versus PDTC (n = 12)]. (**B**) Volcano plot illustrating log_2FC with q-value (-log base 10) for 142 core proteostasis genes in the entire THCA cohort [T3+T4 versus T1+T2 (middle); N1 versus N0 (right)]. Gene categories indicated by coloured spots. (**C**) Venn diagram showing overlap in the 142 core proteostasis gene panel between the GSE276039 (anaplastic thyroid cancer versus PDTC), THCA (T3+T4 versus T1+T2) and THCA (N0 versus N1) datasets. Genes in the 13 gene proteostasis risk score classifier are underlined. (**D**) Representative ROC curves (left) of 3 molecular biomarkers in the BRAF-like, RAI-treated THCA cohort (n = 137). Comparison of ROC analysis (right) using 7 molecular biomarkers in the BRAF-like, RAI-treated THCA cohort (n = 137). AUC, p-values and optimal cut-off expression values (log_2 values) are shown. (**E**) Representative Kaplan-Meier analysis of DFS for the BRAF-like, RAI-treated THCA cohort (n = 124) stratified on high versus low tumoral expression of 7 genes reported as biomarkers of thyroid cancer recurrence; log-rank test. Kaplan-Meier analysis using the 13-gene riskscore classifier to stratify patients is included for comparison. Number (n) of patients per expression/risk sub-group (high/low) and p-values are shown.

Table S1 (related to Fig. 1D, 1E, S1A, S1B). Top 50 Drugs Identified in the Primary High Throughput Screen Using YFP as a Biosensor of Intracellular Iodide

Drug	ΔYFP	Cell Viability (%)	Category	Known or putative target(s)
Antimycin A	5.063	89.30	Piscicide	Autophagy
Demecarium	5.008	77.22	Anticholinesterase	N/A
Ebastine	4.524	83.37	Antihistamine	VCP
Tioconazole	4.254	61.72	Antifungal	Autophagy (ATG4B inhibitor)
Bromocryptine	4.199	51.41	Dopamine agonist	Autophagy
Vanoxerine	4.145	64.00	Dopamine reuptake	N/A
Dequalinium	3.757	54.66	Antimicrobial	N/A
Articaine	3.598	84.90	Anesthetic	N/A
Astemizole	3.259	9.85	Antifungal	Lysosomotrophic, VCP
Nomegestrol	3.189	99.87	Progestogen	N/A
Eucatropine	3.087	96.65	Anticholinergic	N/A
Lithocholic acid	3.078	77.97	Detergent	Autophagy, ER stress
Tosufloxacin	2.959	76.29	Antibiotic	N/A
Clotrimazole	2.923	89.98	Antifungal	VCP
Rosiglitazone	2.854	96.47	Hypoglycemic	Autophagy, mTOR
Vatalanib	2.832	78.49	Anticancer	Tyrosine kinase inhibitor (TKI)
Pyrvinium	2.831	72.32	Anthelminthic	Autophagy
Phentolamine	2.830	108.85	α-adrenergic antagonist	N/A
Atenolol	2.808	98.52	Beta blocker	N/A
Itopride	2.760	80.40	Dopamine D2 antagonist	N/A
Formoterol	2.741	119.65	β(2) -agonist	Autophagy, proteasomal
Fenofibrate	2.606	103.58	Lipid-lowering	Autophagy
Terfenadine	2.578	83.49	Antihistamine	VCP
Clofilium	2.534	39.77	Anti-arrhythmia	N/A
Rivastigmine	2.483	122.32	Anticholinesterase	N/A
Pravastatin	2.467	101.97	Lipid-lowering	HMG-CoA reductase
Fendiline	2.412	70.95	Calcium channel blocker	N/A
Imatinib	2.358	105.36	Anticancer	Autophagy, lysosomotrophic, TKI
Mifepristone	2.341	93.41	Antiprogestogenic	Autophagy, unfolded protein response
Piperacetazine	2.321	85.25	Antipsychotic	N/A
Methyldopate	2.311	103.17	Antihypertensive	N/A
Hesperidin	2.270	68.98	Flavanone glycoside	Autophagy, ER stress
Rufloxacin	2.269	112.38	Antibiotic	N/A
Nortriptyline	2.248	74.00	Antidepressant	Autophagy, lysosomotrophic
Methotrimeprazine	2.222	91.01	Phenothiazine	N/A
Oxyphenbutazone	2.219	62.83	Anti-inflammatory	N/A
Prednisolone	2.209	92.22	Anti-inflammatory	N/A
Phenformin	2.165	102.43	Antidiabetic	Autophagy, mTOR
Clomipramine	2.151	87.88	Antidepressant	Autophagy, lysosomotrophic
Halofantrine	2.143	105.63	Antimalarial	Autophagy, lysosomotrophic
Sisomicin	2.122	147.72	Antibiotic	N/A
Oxantel	2.115	78.93	Anthelminthic	N/A
Trimethoprim	2.108	97.82	Antibiotic	N/A
Chloroquine	2.075	120.91	Antimalarial	Autophagy, lysosomotrophic, vesicular transport
Propidium	2.070	85.28	Intercalation	N/A
Meclozine	2.060	69.82	Antihistamine	Histamine H1 antagonist
Quinidine	2.059	88.15	Anti-arrhythmia	Lysosomotrophic
Ceforanide	2.049	89.90	Antibiotic	N/A
Clonixin	2.009	120.13	Anti-inflammatory	N/A
Tropisetron	2.004	78.57	Antiemetic	N/A

Drugs listed in order of ΔYFP values utilizing TPC-1-NIS cells treated with a 10 μM dose for 24 hr. TPC-1-NIS cell viability (%) after 24 hr drug treatment, drug category and known or putative proteostasis drug targets are shown.

A TPC-1-NIS-YFP cells (> 70% cell viability)

	Drug	AUC	Maximal ΔYFP	Dose (μM)		Drug	AUC	Maximal ΔYFP	Dose (μM)		Drug	AUC	Maximal ΔYFP	Dose (μM)
1	Demecarium	8.02	5.06	25	11	Alverine	3.63	2.52	12.50	21	Levocabastine	1.15	2.10	3.13
2	Pyrvinium	6.73	8.17	0.78	12	Clotrimazole	3.32	2.53	0.20	22	Amikacin	0.65	2.29	0.20
3	Guanabenz	5.56	4.12	1.56	13	Phenformin	3.19	3.97	50	23	Sertaconazole	0.38	2.44	25
4	Proparacaine	5.41	3.87	0.39	14	Sulconazole	3.09	3.83	12.5	24	(R)-(+)Atenolol	3.16	1.87	0.78
5	Niflumic acid	4.95	2.70	1.56	15	Terfenadine	3.00	2.58	6.25	25	Azathioprine	2.93	1.92	12.50
6	Moroxidine	4.86	2.61	0.39	16	Halofantrine	2.28	2.06	3.13	26	Florfenicol	2.61	1.74	0.10
7	Nortriptyline	4.48	2.52	12.50	17	Hexachlorophene	2.00	3.10	50	27	Zimelidine	2.23	1.94	6.25
8	Chlorhexidine	4.24	5.89	6.25	18	Retinoic acid	1.62	2.91	0.39	28	Amrinone	2.11	1.91	3.13
9	Ebastine	4.19	4.52	6.25	19	Disulfiram	1.55	2.78	3.13	29	Praziquantel	1.83	1.71	12.5
10	Ethacrynic acid	3.70	2.22	0.10	20	Hesperidin	1.15	2.76	0.20	30	Articaine	1.22	1.66	1.56

B TPC-1-YFP cells (> 70% cell viability)

	Drug	AUC	Maximal ΔYFP	Dose (μM)		Drug	AUC	Maximal ΔYFP	Dose (μM)
1	Prednisolone	4.95	3.27	0.78	14	Methotrimeprazine	1.38	2.82	50
2	Piperacetazine	3.81	3.02	12.5	15	Guanabenz	2.40	1.94	3.13
3	Demecarium	3.50	4.51	12.5	16	Fenofibrate	2.25	1.59	12.5
4	Pyrvinium	3.37	4.99	0.78	17	Praziquantel	2.22	1.91	0.39
5	Phenformin	3.31	3.90	50	18	Piretanide	1.41	1.86	0.1
6	Mianserine	3.30	4.34	50	19	Niflumic acid	1.26	1.87	0.39
7	Sertaconazole	3.05	3.57	25	20	Ticonazole	1.15	1.54	0.20
8	Ebastine	2.94	4.56	6.25	21	Tridihexethyl	0.92	1.92	50
9	Dexamethasone	2.24	2.03	0.78	22	Retinoic acid	0.83	1.89	0.39
10	Chlorhexidine	2.17	2.49	6.25	23	Clomiphene	0.82	1.81	12.5
11	Ethacrynic acid	1.72	2.02	0.10	24	Clomipramine	0.78	1.67	25
12	Disulfiram	1.69	2.32	0.39	25	Vanoxerine	0.73	1.64	6.25
13	Fendiline	1.58	2.41	12.5	26	Bisacodyl	0.42	1.86	50

C TPC-1-NIS-YFP v TPC-1-YFP cells (> 70% cell viability; i.e adjusted for YFP effects)

	Drug	ΔAUC		Drug	ΔAUC		Drug	ΔAUC
1	Proparacaine	5.31	11	Sulconazole	3.09	21	Hesperidin	1.14
2	Moroxidine	4.57	12	Azathioprine	2.86	22	Levocabastine	1.13
3	Demecarium	4.53	13	Florfenicol	2.61	23	Halofantrine	1.05
4	Nortriptyline	4.24	14	Terfenadine	2.53	24	Articaine	0.95
5	Niflumic acid	3.70	15	Chlorhexidine	2.07	25	Khellin	0.92
6	Alverine	3.41	16	Hexachlorophene	2.00	26	Retinoic acid	0.79
7	Pyrvinium	3.36	17	Ethacrynic acid	1.98	27	Quinidine	0.68
8	Clotrimazole	3.32	18	Zimelidine	1.81	28	Molindone	0.66
9	Guanabenz	3.16	19	Amrinone	1.77	29	Astemizole	0.60
10	(R)-(+)Atenolol	3.16	20	Ebastine	1.25	30	Amikacin	0.54

D

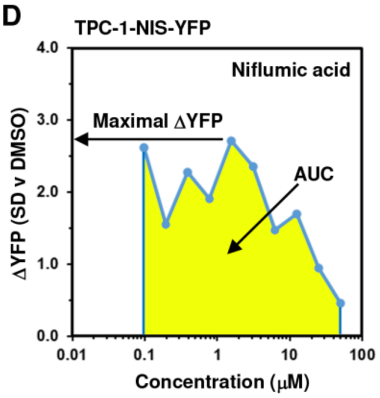

E

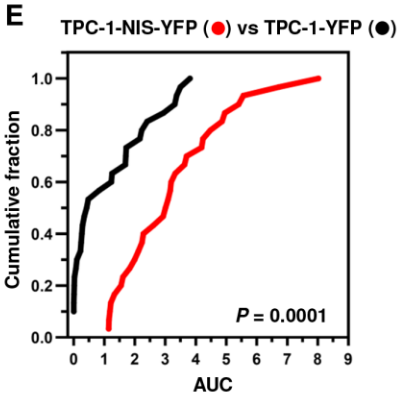

Table S2 (related to Fig. 1F, S1C). Ranking of Drug Efficacy to Enhance Intracellular Iodide Adjusting for Multiple Doses, YFP-Only Effects, and Cell Viability

(**A**) Top 30 drugs ranked on area under the curve (AUC) values derived from ΔYFP values of TPC-1-NIS-YFP cells treated with multiple drug doses (0.1-50 μM) and > 70% cell viability. Maximal ΔYFP value and associated dose (μM) for each drug are shown. Drugs with maximal ΔYFP values < 2 were ranked lower. (**B**) Same as (A) but with TPC-1-YFP cells. (**C**) Top 30 drugs ranked on ΔAUC values, i.e., difference in AUC values between drug-treated TPC-1-NIS-YFP and TPC-1-YFP cells. (**D**) Representative dose response YFP-iodide profile highlighting pharmacologic parameters used in analysis, i.e., area under the curve (AUC) and maximal ΔYFP values, in TPC-1-NIS-YFP cells treated with niflumic acid. (**E**) Comparison of drug efficacy in thyroid cells with different NIS levels. Cumulative frequency distribution plot comparing AUC values for top 30 drugs identified to increase intracellular iodide in parental TPC-1-YFP and TPC-1-NIS-YFP cells; P-value determined by the Kolmogorov-Smirnov test.

Table S3 (related to Fig. 3A, Table 1). Chemical Structure of Putative VCP inhibitors

Drug	2D Structure	3D Structure	Supporting Evidence
Carebastine	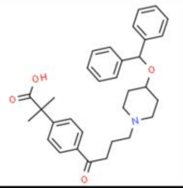	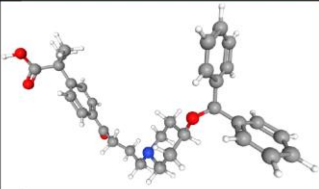	Drug Combination, RAI Uptake & VCP siRNA assays (this study)<br><br>Active carboxylic acid metabolite of ebastine
Fexofenadine	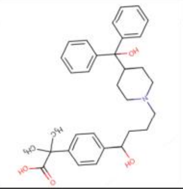	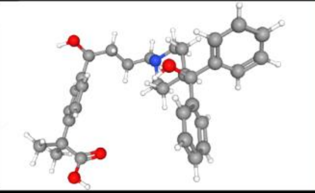	RAI Uptake assays (this study)<br><br>Active metabolite of terfenadine
Terfenadine	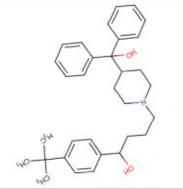	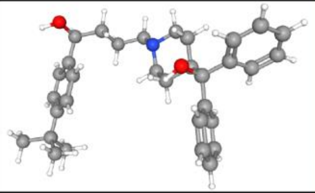	CMAP (L1000) & RAI Uptake assays (this study)<br><br>CMAP (build 02): MCF7 Breast cells (p = 3.0E-31) (Segura-Cabrera et al., 2017)
Disulfiram	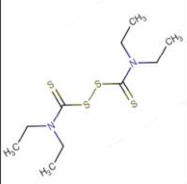	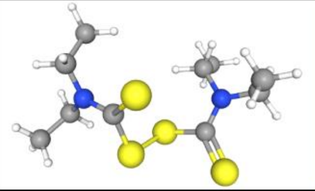	Drug Combination & RAI Uptake assays (this study)<br>Disulfiram Metabolite (DTC) Binds NPL4 to Disable the VCP-NPL4-UFD1 Pathway (Skrott et al., 2017)
Ebastine	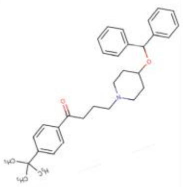	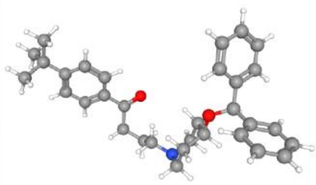	Drug Combination & RAI Uptake assays (this study)<br><br>Structure-Based Virtual Screening, Cell-Based & Biochemical assays (Segura-Cabrera et al., 2017)<br><br>RAI Uptake, VCP siRNA & Cell Surface Biotinylation (CSB) assays (Fletcher et al., 2020)
Astemizole	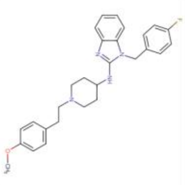	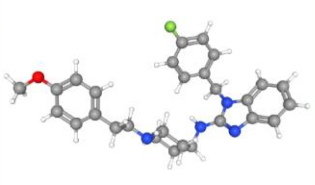	Drug Combination & RAI Uptake assays (this study)<br>CMAP (build 02): MCF7 Breast cells (p = 8.4E-39) Structure-based Virtual Screening, Cell-Based & Biochemical assays (Segura-Cabrera et al., 2017)<br>RAI uptake, VCP siRNA & CSB assays (Fletcher et al., 2020)
Clotrimazole	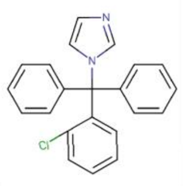	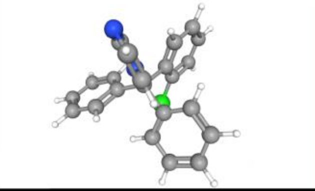	CMAP (L1000), Drug Combination & RAI Uptake assays (this study)<br>CMAP (build 02): MCF7 Breast cells (p = 4.0E-46) Structure-Based Virtual Screening Cell-Based & Biochemical assays (Segura-Cabrera et al., 2017)<br>RAI uptake, VCP siRNA & CSB assays (Fletcher et al., 2020)
NMS-873	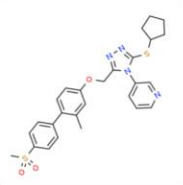	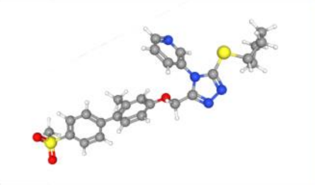	Well-established VCP inhibitor<br>Used as a control drug for VCP inhibitor studies<br><br>Drug combination & RAI uptake assays (this study)

2D and 3D images of putative VCP inhibitors are shown, as well as supporting evidence for their ability to target VCP function and enhance NIS function to increase radioiodide uptake. Source: ChemSpider; PubChem.

Table S4 (related to Fig. 5, S4, S6, S7). Panel of 142 Core Proteostasis Genes

Unfolded Protein Response (UPR)	Proteasomal			Autophagy		Transport (protein, vesicular)			
ATF4	*PSMA1*	*PSMC1*	*PSMD12*	*ATG2A*	*EPG5*	*AP1AR*	*AP3M2*	*COPG1*	*SEC23A*
ATF6	*PSMA2*	*PSMC2*	*PSMD13*	*ATG2B*	*ERN1*	*AP1B1*	*AP3S1*	*COPG2*	*SEC23B*
DDIT3 (CHOP)	*PSMA3*	*PSMC3*	*PSMD14*	*ATG3*	*GABARAPL1 (ATG8)*	*AP1G1*	*AP3S2*	*COPZ1*	*SEC24A*
EIF2AK3 (PERK)	*PSMA4*	*PSMC4*	*PSME1*	*ATG4A*	*SQSTM1 (p62)*	*AP1G2*	*AP4B1*	*COPZ2*	*SEC24B*
HSPA5 (GRP78)	*PSMA5*	*PSMC5*	*PSME2*	*ATG4B*	*ULK1 (ATG1)*	*AP1M1*	*AP4E1*	*DNM2*	*SEC24C*
XBP1	*PSMA6*	*PSMC6*	*PSME3*	*ATG4C*	*WDR45*	*AP1M2*	*AP4M1*	*DTNBP1*	*SEC24D*
	PSMA7	*PSMD1*	*PSME4*	*ATG4D*	*WDR45B*	*AP1S1*	*ARCN1*	*EHD1*	*SEC31A*
	PSMB1	*PSMD2*	*PSMF1*	*ATG5*	*WIPI1 (ATG18)*	*AP1S2*	*BLOC1S3*	*EHD2*	*SEC31B*
	PSMB2	*PSMD3*	*UBB*	*ATG7*	*WIPI2 (ATG18B)*	*AP1S3*	*CAV1*	*EHD4*	*SNAP29*
	PSMB3	*PSMD4*	*UBC*	*ATG9A*		*AP2A1*	*CAV2*	*HPS1*	*VAMP1*
	PSMB4	*PSMD5*	*VCP*	*ATG10*		*AP2A2*	*CLTA*	*HPS3*	*VAMP2*
	PSMB5	*PSMD6*		*ATG12*		*AP2B1*	*CLTB*	*HPS4*	*VAMP3*
	PSMB6	*PSMD7*		*ATG13*		*AP2M1*	*CLTC*	*HPS5*	*VPS13B*
	PSMB7	*PSMD8*		*ATG14*		*AP2S1*	*COPA*	*HPS6*	*VPS33B*
	PSMB8	*PSMD9*		*ATG16L1*		*AP3B1*	*COPB1*	*PLIN3*	
	PSMB9	*PSMD10*		*ATG16L2*		*AP3D1*	*COPB2*	*SAR1A*	
	PSMB10	*PSMD11*		*BECN1 (ATG6)*		*AP3M1*	*COPE*	*SEC13*	

Functional categories of core proteostasis genes used in study which include: the unfolded protein response (UPR, 6 genes, orange), proteasomal degradation (45 genes, red), autophagy (26 genes, green) and transport (protein/ vesicular, 65 genes, blue).

Table S5 (related to Fig. 5F). Core Proteostasis Genes are Predictive Indicators of an Increased Risk of Recurrence

Gene	Category	High vs Low Expression Groups					
		Cut-Off Value	B	Hazard Ratio	95.0% CI		p-value
					Lower	Upper	
AP3D1	Transport	11.89	1.311	3.709	1.574	8.741	0.003
AP4B1	Transport	8.23	-1.620	0.198	0.058	0.672	0.009
ATG2A	Autophagy	10.10	1.747	5.736	1.928	17.063	0.002
ATG9A	Autophagy	10.48	1.414	4.111	1.505	11.224	0.006
BECN1	Autophagy	10.76	1.065	2.901	1.222	6.892	0.016
HPS1	Transport	10.75	1.481	4.399	1.611	12.010	0.004
HSPA5	UPR	14.04	-1.437	0.238	0.08	0.707	0.010
PSMD2	Proteasomal	11.76	1.221	3.390	1.241	9.256	0.017
PSMD8	Proteasomal	11.47	0.892	2.441	1.029	5.794	0.043
PSMD11	Proteasomal	9.48	1.591	4.908	1.650	14.601	0.004
SEC24C	Transport	11.52	1.414	4.112	1.506	11.228	0.006
SQSTM1	Autophagy	14.15	1.179	3.251	1.191	8.876	0.021
VCP	Proteasomal	12.66	2.374	10.744	1.442	80.073	0.021

Univariate Cox regression analysis in the BRAF-like, RAI-treated THCA cohort stratified using optimal expression cut-off values for 13 proteostasis genes. Cut-off value- $\log_2$ expression value; B- regression coefficient.

Table S6 (related to Fig. 5H, I). Univariate and Multivariate Analysis of the RAI-Treated (BRAF-like) and Non-RAI Treated THCA Cohort

A

Clinical Variable	BRAF-like, RAI-treated (n = 124)					Non-RAI treated (n = 151)				
	n	Univariate		Multivariate		n	Univariate		Multivariate	
		p-value	HR (95% CI)	p-value	HR (95% CI)		p-value	HR (95% CI)	p-value	HR (95% CI)
**Age, years (±SD)**										
< 50	79	0.072	2.288 (0.929-5.634)	0.403	2.054 (0.380-11.105)	83	0.857	1.159 (0.234-5.744)	0.548	0.567 (0.089-3.604)
> 50	45					68				
**Gender**										
Male	39	0.994	0.996 (0.378-2.623)	0.807	0.877 (0.308-2.500)	28	0.300	2.456 (0.449-13.439)	0.216	3.116 (0.516-18.826)
Female	85					123				
**Stage**										
I + II	70	0.114	2.085 (0.838-5.185)	0.856	0.855 (0.158-4.634)	125	0.244	2.744 (0.502-15.004)	0.677	2.104 (0.063-69.879)
III + IV	54					26				
**T stage**										
T1 + T2	62	0.222	1.789 (0.704-4.544)	0.785	1.149 (0.424-3.113)	120	0.403	2.063 (0.378-11.272)	0.533	2.877 (0.104-79.966)
T3 + T4	62					31				
**Node stage**										
N0	34	0.449	1.531 (0.508-4.617)	0.109	2.587 (0.810-8.264)	110	0.642	0.600 (0.070-5.147)	0.412	0.398 (0.044-3.607)
N1	90					41				
**Risk score (13 gene classifier)**										
High	50	**$7.0x10^{-4}$**	**32.614 (4.350-244.534)**	**$7.5x10^{-4}$**	**32.781 (4.309-249.369)**	33	0.445	0.035 (0.000-190.755)	0.974	N/A
Low	74					118				

B

Clinical Variable	BRAF-like, RAI-treated (n = 124)					Non-RAI treated (n = 151)				
	n	Univariate		Multivariate[1]		n	Univariate		Multivariate[1]	
		p-value	HR (95% CI)	p-value	HR (95% CI)		p-value	HR (95% CI)	p-value	HR (95% CI)
**VCP expression**										
High	84	**0.031**	**9.166 (1.223-68.679)**	**0.038**	**8.472 (1.122-63.969)**	51	0.940	1.067 (0.195-5.830)	0.872	0.867 (0.154-4.876)
Low	40					100				

(**A**) n, number; HR, hazard ratio; CI, confidence interval. P-values in bold were less than 0.05 and considered statistically significant. Some patients in the BRAF-like, RAI-treated (n = 13) and non-RAI treated cohorts (n = 16) were not included in univariate and multivariate analysis of the THCA dataset due to missing clinical variables. (**B**) Same as (A) except VCP expression was used instead of the 13-gene risk score classifier in multivariate analysis[1].

Table S7 (related to Fig. 5H, I). Univariate and Multivariate Analysis of the RAI-Treated and Entire THCA Cohorts

A

Clinical Variable	RAI-treated (n = 226)					THCA (n = 438)				
	n	Univariate		Multivariate		n	Univariate		Multivariate	
		p-value	HR (95% CI)	p-value	HR (95% CI)		p-value	HR (95% CI)	p-value	HR (95% CI)
**Age, years (±SD)**										
< 50<br>> 50	136<br>90	**0.039**	**2.113 (1.039-4.296)**	0.352	1.771 (0.532-5.897)	250<br>188	0.074	1.732 (0.949-3.162)	0.938	1.036 (0.430-2.496)
**Gender**										
Male<br>Female	74<br>152	0.678	1.169 (0.560-2.44)	0.537	1.271 (0.593-2.725)	119<br>319	0.352	1.354 (0.715-2.564)	0.456	1.282 (0.668-2.460)
**Stage**										
I + II<br>III + IV	133<br>93	**0.028**	**2.228 (1.088-4.565)**	0.719	0.795 (0.227-2.779)	291<br>147	**0.001**	**2.854 (1.561-5.217)**	0.285	1.689 (0.646-4.415)
**T stage**										
T1 + T2<br>T3 + T4	116<br>110	**0.010**	**2.758 (1.270-5.99)**	0.053	2.274 (0.990-5.223)	268<br>170	**0.002**	**2.638 (1.421-4.898)**	0.243	1.516 (0.754-3.049)
**Node stage**										
N0<br>N1	81<br>145	0.391	1.404 (0.646-3.050)	0.349	1.471 (0.656-3.302)	221<br>217	**0.046**	**1.892 (1.010-3.542)**	0.413	1.32 (0.679-2.568)
**Risk score (13 gene classifier)**										
High<br>Low	80<br>146	**$8.7x10^{-7}$**	**11.070 (4.248-28.851)**	**$2.0x10^{-6}$**	**10.577 (3.996-28.001)**	131<br>307	**$3.8x10^{-7}$**	**5.523 (2.801-10.038)**	**$5.0x10^{-6}$**	**4.553 (2.379-8.714)**

B

Clinical Variable	RAI-treated (n = 226)					THCA (n = 438)				
	n	Univariate		Multivariate[1]		n	Univariate		Multivariate[1]	
		p-value	HR (95% CI)	p-value	HR (95% CI)		p-value	HR (95% CI)	p-value	HR (95% CI)
**VCP expression**										
High<br>Low	78<br>148	0.102	1.801 (0.890-3.645)	0.172	1.654 (0.804-3.404)	163<br>275	0.159	1.539 (0.845-2.803)	0.193	1.491 (0.817-2.722)

(**A**) n, number; HR, hazard ratio; CI, confidence interval. P-values in bold were less than 0.05 and considered statistically significant. Some patients in the RAI-treated (n = 31) and entire THCA cohorts (n = 50) were not included in univariate and multivariate analysis of the THCA dataset due to missing clinical variables. (**B**) Same as (A) except VCP expression was used instead of the 13-gene risk score classifier in multivariate analysis[1].
