# Supplementary material for: Targeting non-canonical pathways as a strategy to modulate the sodium iodide symporter
Source: Cell Chem Biol. 2022 Mar 17;29(3):502–516.e7. doi: 10.1016/j.chembiol.2021.07.016 (PMC8958605; doi:10.1016/j.chembiol.2021.07.016)
Supplement: Document S2. Article plus supplemental information [file mmc2.pdf]

Article

Cell Chemical Biology

# Targeting non-canonical pathways as a strategy to modulate the sodium iodide symporter

## Graphical abstract

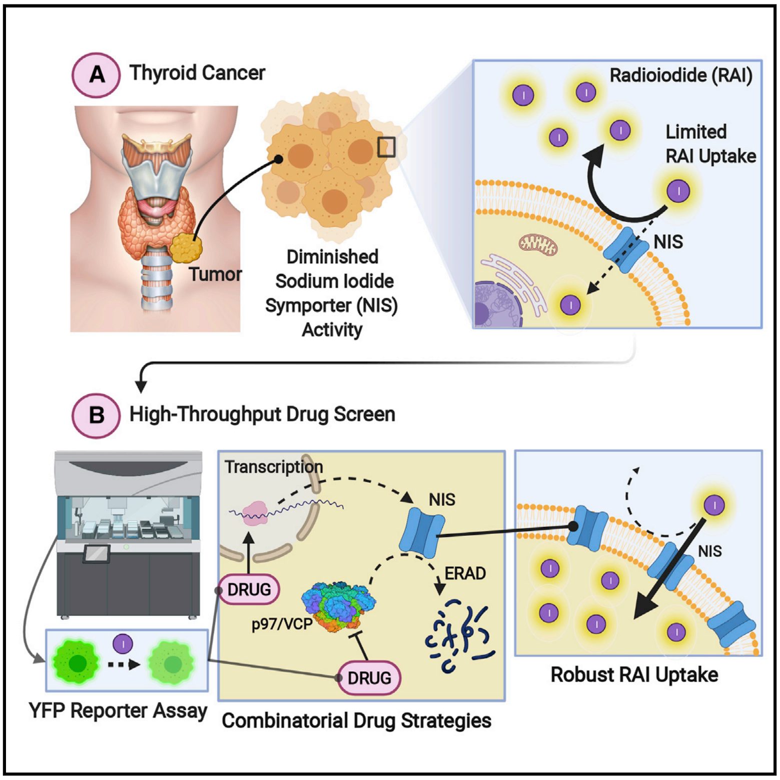


## Authors

Martin L. Read, Katie Brookes,
Caitlin E.M. Thornton, ...,
Kristien Boelaert, Vicki E. Smith,
Christopher J. McCabe

## Correspondence

mccabcjz@bham.ac.uk

## In brief

Read et al. investigate the key druggable non-canonical pathways to recover function of the sodium iodide symporter (NIS). They identify mechanisms in NIS intracellular processing that could be exploited therapeutically for patients treated with radioiodide who typically have poorer clinical outcomes.

## Highlights

- YFP biosensor identifies FDA-approved drugs that increase intracellular iodide
- Proteostasis pathways central to control of NIS symporter activity are identified
- A 13-gene risk score classifier predictive of thyroid cancer recurrence is devised
- A model for targetable steps of intracellular processing of NIS is proposed

Read et al., 2022, Cell Chemical Biology 29, 502–516
March 17, 2022 © 2021 The Author(s). Published by Elsevier Ltd.
https://doi.org/10.1016/j.chembiol.2021.07.016

CellPress

CellPress
OPEN ACCESS

Cell Chemical Biology

Article

# Targeting non-canonical pathways as a strategy to modulate the sodium iodide symporter

**Martin L. Read,[1,7] Katie Brookes,[1,7] Caitlin E.M. Thornton,[1] Alice Fletcher,[1] Hannah R. Nieto,[1] Mohammed Alshahrani,[1] Rashida Khan,[1] Patricia Borges de Souza,[2] Ling Zha,[1] Jamie R.M. Webster,[3] Luke J. Alderwick,[4] Moray J. Campbell,[5] Kristien Boelaert,[6] Vicki E. Smith,[1] and Christopher J. McCabe[1,8,*]**

[1]Institute of Metabolism and Systems Research (IMSR), and Centre of Endocrinology, Diabetes and Metabolism (CEDAM), University of Birmingham, Birmingham B15 2TT, UK

[2]Section of Endocrinology, Department of Medical Sciences, University of Ferrara, Ferrara 44124, Italy

[3]Protein Expression Facility, College of Medical and Dental Sciences, University of Birmingham, B15 2TT, UK

[4]Birmingham Drug Discovery Facility, School of Biosciences, University of Birmingham, Birmingham B15 2TT, UK

[5]Division of Pharmaceutics and Pharmacology, The Ohio State University, College of Pharmacy, Columbus, OH 43210, USA

[6]Institute of Applied Health Research, University of Birmingham, Birmingham B15 2TT, UK

[7]These authors contributed equally

[8]Lead contact

*Correspondence: mccabcjz@bham.ac.uk

https://doi.org/10.1016/j.chembiol.2021.07.016

## SUMMARY

**The sodium iodide symporter (NIS) functions to transport iodide and is critical for successful radioiodide ablation of cancer cells. Approaches to bolster NIS function and diminish recurrence post-radioiodide therapy are impeded by oncogenic pathways that suppress NIS, as well as the inherent complexity of NIS regulation. Here, we utilize NIS in high-throughput drug screening and undertake rigorous evaluation of lead compounds to identify and target key processes underpinning NIS function. We find that multiple proteostasis pathways, including proteasomal degradation and autophagy, are central to the cellular processing of NIS. Utilizing inhibitors targeting distinct molecular processes, we pinpoint combinatorial drug strategies giving robust >5-fold increases in radioiodide uptake. We also reveal significant dysregulation of core proteostasis genes in human tumors, identifying a 13-gene risk score classifier as an independent predictor of recurrence in radioiodide-treated patients. We thus propose and discuss a model for targetable steps of intracellular processing of NIS function.**

## INTRODUCTION

For ~80 years, radioiodide (RAI) has been the central post-surgical treatment for patients with differentiated thyroid cancer (DTC). However, at least 25% of DTC patients have radioiodide-refractory thyroid cancer, being unable to uptake adequate radioiodide for effective therapeutic ablation (Schlumberger et al., 2014). This is especially problematic for DTC patients with metastatic disease who have a life expectancy of 2.5–3.5 years (Lirov et al., 2017). New therapeutic strategies are urgently required to address this clear unmet medical need with >43,000 lives lost worldwide to thyroid cancer per annum, which is estimated to rise to 74,733 by 2040 (Ferlay et al., 2019).

Sodium iodide symporter (NIS) is the sole transporter responsible for specific cellular iodide uptake (Dai et al., 1996); exploitation of its function permits specific targeting of high-energy β-emitting $^{131}$I to destroy remaining thyroid cells post-surgery, and target metastases. The central mechanisms that underlie radioiodide refractoriness are decreased levels of NIS expression and/or its diminished targeting to the plasma membrane (PM) (Spitzweg et al., 2001). The regulation of NIS is principally accomplished via transcription factors, histone acetylation, post-translational modifications, hormonal signaling, and by iodide itself (Arriagada et al., 2015; Passon et al., 2012; Ravera et al., 2017; Zhang et al., 2014). These levels of control are generally disrupted in cancer by a plethora of mechanisms, including altered histone acetylation and methylation of the NIS promoter (Passon et al., 2012; Zhang et al., 2014), distorted miRNA expression (Cancer Genome Atlas Research, 2014; Riesco-Eizaguirre et al., 2015), increased oxidative stress (Azouzi et al., 2017), and changed growth factor signaling (Knauf et al., 2011; Riesco-Eizaguirre et al., 2009). Thus, in addressing repressed NIS function in thyroid cancer, this inherent multiplicity of regulation adds significantly to the complexity of potential therapeutic strategies.

Extensive pre-clinical and clinical studies have attempted to enhance NIS expression and function, focusing mainly on "redifferentiation agents," which stimulate the expression of thyroid-specific genes including NIS. An integral component is the canonical mitogen-activated protein kinase (MAPK) signaling


This is an open access article under the CC BY license (http://creativecommons.org/licenses/by/4.0/).

CellPress
OPEN ACCESS

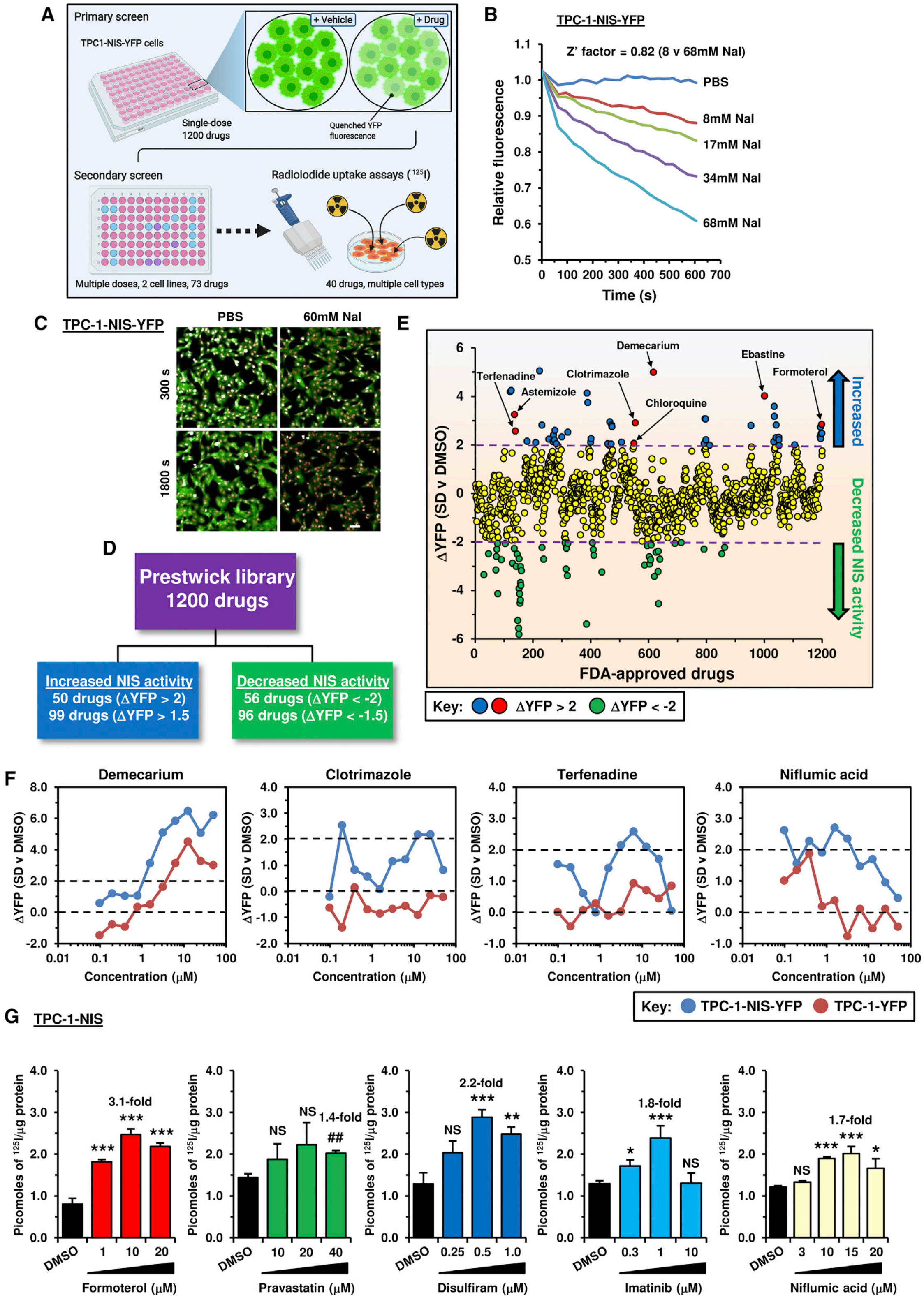


**Figure 1. YFP-based biosensor strategy to identify drugs that increase intracellular iodide**

(A) Schematic of high-throughput screening approach used to identify compounds that increase intracellular iodide. Created with BioRender.com. See also Figure S1A.

(B) Validation of YFP-iodide assay in TPC-1-NIS-YFP cells using increasing NaI (0–68 mM).

(legend continued on next page)

pathway which has a pivotal role in DTC, with ~70% of thyroid carcinomas (THCAs) driven by mutations that directly stimulate this pathway (Zaballos and Santisteban, 2017). Because constitutive activation of the MAPK pathway in thyroid cancer frequently represses NIS expression and function and is associated with decreased radioiodide uptake and poor patient prognosis, many studies have focused on MAPK inhibitors (Dunn et al., 2019; Ho et al., 2013). However, sustained inhibition of the MAPK pathway will be needed to maximize responses to radioiodide therapy and help overcome the comparatively ineffective suppression of oncogene-driven signaling by RAF and MEK inhibitors (Nagarajah et al., 2016). Other valuable approaches have addressed transcriptional and epigenetic alterations (Kitazono et al., 2001; Mancikova et al., 2014), including the use of histone deacetylase (HDAC) inhibitors (Pugliese et al., 2013; Wachter et al., 2018). However, while some drugs have enhanced NIS expression in a subset of patients, new approaches are required to address the intricacy of altered NIS regulation in thyroid neoplasia.

Here, we utilized high-throughput screening (HTS) with the aim of identifying key targetable processes underlying NIS function. HTS of NIS function has been reported, but primarily to identify pollutants that disrupt NIS activity (Buckalew et al., 2020; Hallinger et al., 2017). Our study is therefore—to our knowledge—the first to directly screen and identify FDA-approved drugs capable of enhancing radioiodide uptake on a large pharmacological scale. Importantly, our findings reveal cellular processes that modify radioiodide uptake outside of the canonical pathways of NIS processing, leading to an improved mechanistic understanding of endogenous NIS function, which is subverted in cancer, as well as facilitating the identification of key target genes to construct an independent predictive risk model of recurrent thyroid cancer.

## RESULTS

### Drug screening identifies novel compounds that alter surrogate NIS activity

We first adapted and validated yellow fluorescent protein (YFP)-iodide sensing to an HTS of 1,200 drugs (Prestwick Chemical Library; 95% FDA approved) as outlined (Figures 1A–1C and S1A). Primary screening in TPC-1-NIS-YFP cells revealed that ~8% of drugs (99/1,200; ΔYFP > 1.5) quenched YFP fluorescence (Figures 1D and 1E) with good overall cell viability (≥70% viability for >90% of drugs; Figure S1B; Table S1). For 73 leading drugs we performed secondary screening at 10 different drug doses and compared YFP fluorescence in control TPC-1-YFP cells, as well as cell viability (Figures 1F and S1C). Overall, adjusting for cell viability and YFP-only effects, we ranked the top drugs that specifically dimmed YFP fluorescence as a marker of increased cellular iodide uptake (Table S2).

### Impact of drugs on thyroid cancer and human primary cells

We tested 40 of our top drugs in radioiodide uptake assays in TPC-1 cells stably expressing NIS; in total 17 of these gave significant dose-dependent increases in $^{125}$I uptake (Figures 1G and S1D; Table 1). To investigate potential mechanisms, we initially assessed the impact of seven of these drugs on NIS protein expression in total cell lysates; three drugs (formoterol, pravastatin, and disulfiram) significantly increased NIS protein levels both in TPC-1 and 8505C cells stably expressing NIS (Figures 2A–2D). Importantly, increased NIS protein matched greater radioiodide uptake induced by these three drugs in 8505C-NIS cells (Figure 2E). Formoterol and pravastatin further induced NIS mRNA expression in TPC-1-NIS cells (Figure 2F). Thus, whereas formoterol and pravastatin act transcriptionally, disulfiram exerts posttranscriptional effects on NIS expression. In contrast, imatinib, niflumic acid, or vatalanib did not significantly alter NIS mRNA or protein expression, and their well-characterized inhibitory effects on ERK/MAPK signaling (Luo et al., 2015; Montor et al., 2018) may instead contribute toward enhancing radioiodide uptake.

TPC-1 and 8505C thyroid cancer cells have inherently dysregulated cellular signaling (Landa et al., 2019). We therefore examined whether drugs identified from our HTS would modulate endogenous NIS function in non-transformed human primary thyroid cells. Significant induction of radioiodide uptake (>1.5-fold) was apparent for 15 drugs, including chloroquine, disulfiram, formoterol, niflumic acid, and pravastatin (Figures 2G and S1E; Table 1). Of these, 13 drugs induced significant $^{125}$I uptake in thyroid cancer cells stably expressing NIS as well as in human primary thyrocytes, implicating targeting of pathways which control normal physiological NIS function (Table 1). Half-maximal effective concentration ($EC_{50}$) values were broadly comparable between TPC-1-NIS cells and human primary thyrocytes (Figure 2H).

Categorization of drugs revealed a high proportion known to modulate proteostasis (20/50 drugs), including key endoplasmic reticulum-associated protein degradation (ERAD) processes (Figure 2I; Table S1). To predict in more detail the pathways of NIS processing that might be targeted by our drug approaches, we next used the Connectivity Map hierarchical clustering from available drug data, which identified a large cluster strongly associated with genes implicated in the proteasomal pathway and unfolded protein response (Figure 3A; cluster 5). Strikingly, most drugs (Figure 3A; cluster 5) were associated with loss of function of valosin-containing protein (VCP), recently reported as a critical component of ERAD that increased NIS proteolysis, permitting less NIS to be trafficked to the PM (Fletcher et al., 2020). In keeping with this, seven drugs validated for enhancing $^{125}$I uptake in TPC-1-NIS cells (Table 1) were linked with inhibiting VCP, including ebastine and astemizole

(C) Opera Phenix live cell imaging of TPC-1-NIS-YFP cells treated with 60 mM NaI (300 and 1,800 s). Scale bar, 20 μM.
(D) Summary of drugs identified with differential YFP fluorescence (ΔYFP). Drugs associated with negligible cellular viability were not counted.
(E) Relative YFP fluorescence (ΔYFP) of TPC-1-NIS-YFP cells treated with 1,200 drugs (Prestwick Chemical Library, 10 μM, 24 h) versus DMSO. See also Figure S1B and Table S1.
(F) Representative drug dose-response YFP-iodide profiles in TPC-1-NIS-YFP and TPC-1-YFP cells. See also Figure S1C.
(G) Radioiodide uptake of TPC-1-NIS cells treated with drugs at indicated doses for 24 h. See also Figure S1D and Table 1.
Data presented as mean ± SEM, n = 3–6, one-way ANOVA followed by Dunnett's post hoc test) (NS, not significant; *p < 0.05, **p < 0.01, ***p < 0.001).

**Table 1. Comparison of drug efficacy on radioiodide uptake**

| A | Drug | Mean value | TPC-1-NIS cells n[a] | Dose (μM)[b] | RAI uptake[c] | p value[d] | Human primary thyrocytes n[a] | Dose (μM)[b] | RAI uptake[c] | p value[d] |
|---|---|---|---|---|---|---|---|---|---|---|
| 1 | carebastine | 3.45 | 3 | 1.0 | 3.8 ± 0.4 | 0.003 | 3 | 0.75 | 3.1 ± 0.6 | 0.040 |
| 2 | ebastine | 3.30 | 3 | 0.5 | 3.0 ± 0.5 | 0.020 | 3 | 0.25 | 3.6 ± 0.3 | $2.50 \times 10^{-3}$ |
| 3 | chloroquine[e] | 3.00 | 3 | 50 | 2.6 ± 0.4 | 0.012 | 3 | 50 | 3.4 ± 0.1 | $9.00 \times 10^{-4}$ |
| 4 | SAHA | 2.95 | 5 | 5 | 4.3 ± 0.2 | $5.50 \times 10^{-7}$ | 6 | 5 | 1.6 ± 0.1 | $6.90 \times 10^{-7}$ |
| 5 | astemizole | 2.80 | 3 | 0.25 | 3.5 ± 0.4 | 0.020 | 3 | 0.5–0.75 | 2.1 ± 0.1 | $7.00 \times 10^{-4}$ |
| 6 | formoterol | 2.65 | 3 | 10 | 3.1 ± 0.2 | 0.001 | 3 | 1.0 | 2.2 ± 0.1 | $5.40 \times 10^{-6}$ |
| 7 | clotrimazole | 2.60 | 3 | 0.25 | 3.3 ± 0.3 | 0.004 | 3 | 0.75 | 1.9 ± 0.2 | 0.019 |
| 8 | vatalanib | 2.50 | 4 | 1.0 | 1.6 ± 0.1 | 0.002 | 3 | 10 | 3.4 ± 0.3 | 0.002 |
| 9 | disulfiram | 2.35 | 5 | 0.5 | 2.2 ± 0.1 | 0.001 | 6 | 0.5 | 2.5 ± 0.1 | $8.90 \times 10^{-6}$ |
| 10 | imatinib | 2.20 | 4 | 1.0 | 1.8 ± 0.2 | 0.020 | 3 | 1.0 | 2.6 ± 0.1 | 0.023 |
| 11 | fexofenadine | 2.05 | N/A | N/A | N/A | N/A | 3 | 2.0 | 4.1 ± 1.0 | 0.047 |
| 12 | niflumic acid | 1.75 | 4 | 15 | 1.7 ± 0.1 | $4.00 \times 10^{-4}$ | 4 | 20 | 1.8 ± 0.1 | $2.40 \times 10^{-5}$ |
| 13 | terfenadine | 1.75 | N/A | N/A | N/A | N/A | 3 | 1.0 | 3.5 ± 0.6 | 0.027 |
| 14 | pravastatin | 1.60 | 3 | 40 | 1.4 ± 0.1 | 0.003 | 3 | 40 | 1.8 ± 0.2 | 0.020 |
| 15 | phenformin | 1.60 | 3 | 250 | 1.9 ± 0.1 | 0.001 | 3 | 50 | 1.3 ± 0.1 | NS |
| 16 | zimelidine | 1.50 | 3 | 1.0 | 1.1 ± 0.01 | 0.003 | 3 | 1.0 | 1.9 ± 0.1 | 0.004 |

| B | Drug combinations | | | | | | | | | | | |
|---|---|---|---|---|---|---|---|---|---|---|---|---|
| Cell types | NFA CQ | SAHA CQ | DSF CQ | NFA SAHA | DSF SAHA | IMAT SAHA | EBT SAHA | CBT SAHA | AST SAHA | CLOT SAHA | EBT SEL | CBT SEL |
| Human thyrocytes | 7.3 | 4.3 | 3.5 | 2.4 | 1.1 | 1.5 | | | | | | |
| TPC-1-NIS | 0.9 | 8.3 | 1.8 | 2.2 | 3.3 | 3.0 | 6.3 | 5.2 | 7.9 | 6.8 | | |
| 8505C-NIS | 1.0 | 5.6 | 2.3 | 3.0 | 3.9 | 4.8 | | | | | | |
| Parental TPC-1 | | 3.1 | | | | 4.2 | 5.2 | 6.7 | | | 4.5 | 4.4 |
| Parental 8505C | | | | | | | 3.5 | 5.9 | | | 2.5 | 2.4 |
| Parental MDA-MB-231 | | | | | | | 10.5 | 20.4 | | | 7.1 | 4.1 |
| Mean value | 3.1 | 5.3 | 2.5 | 2.5 | 2.8 | 3.4 | 6.4 | 9.6 | 7.9 | 6.8 | 4.7 | 3.6 |

A, Drugs ranked on mean radioiodide uptake in TPC-1-NIS cells and human primary thyrocytes at maximal biological effect.
B, Mean radioiodide uptake in multiple cell types using drug combinations as indicated. Drugs included: niflumic acid (NFA), chloroquine (CQ), disulfiram (DSF), imatinib (IMAT), ebastine (EBT), carebastine (CBT), astemizole (AST), clotrimazole (CLOT), selumetinib (SEL), and suberoylanilide hydroxamic acid (SAHA).
N/A, not applicable; NS, not significant.
[a]Number of biological replicates performed for each drug treatment. Each biological replicate typically consisted of three to four technical replicates.
[b]Drug dose to achieve maximal radioiodide uptake. Astemizole data is compiled from treating human primary thyrocytes with two different doses (0.5 and 0.75 μM).
[c]Mean fold increase in $^{125}I$ uptake ± SEM for each drug relative to 0.1% DMSO as vehicle control, except for chloroquine (0.5% PBS), formoterol (0.2% DMSO), pravastatin (0.4% DMSO), and phenformin (0.25% ETOH).
[d]p values determined using the unpaired two-tailed t test.
[e]Cells treated for 24 h before assaying $^{125}I$ uptake, except for chloroquine in which cells were treated for 8 h.

(Table S3). In addition, we established using NanoBiT that increasing doses of the VCP inhibitor CB5083 impacted directly on the stringency of the NIS: VCP interaction (Figure 3B).

## Mechanistic interaction between VCP inhibitors and SAHA increases radioiodide uptake

Targeting HDAC activity along with VCP to maximize radioiodide uptake has not been investigated. Suberoylanilide hydroxamic acid (SAHA) is a well-characterized FDA-approved HDAC inhibitor, induces robust NIS mRNA expression in thyroid cells (Cheng et al., 2016; Puppin et al., 2005; Wachter et al., 2018), and was shown to improve radioiodide uptake in one of three patients enrolled in a phase 1 trial (Kelly et al., 2005). To better understand the mechanistic impact of putative VCP inhibitors, we next addressed the hypothesis that SAHA treatment of thyroid cancer cells would induce NIS mRNA expression, and that increased NIS protein might then "benefit" from inhibition of VCP via repressed ERAD to enhance radioiodide uptake. As expected, SAHA induced large dose-dependent increases in NIS protein in stable TPC-1 and 8505C cells, accompanied by marked increases in radioiodide uptake (Figures S1F–S1I). Importantly, co-treatment with SAHA and the VCP inhibitors ebastine (EBT), astemizole (AST), or clotrimazole (CLOT) (Segura-Cabrera et al., 2017) led to significant increases in radioiodide uptake compared with SAHA alone (Figures 3C and 3D). This was supported by findings for the established VCP inhibitors

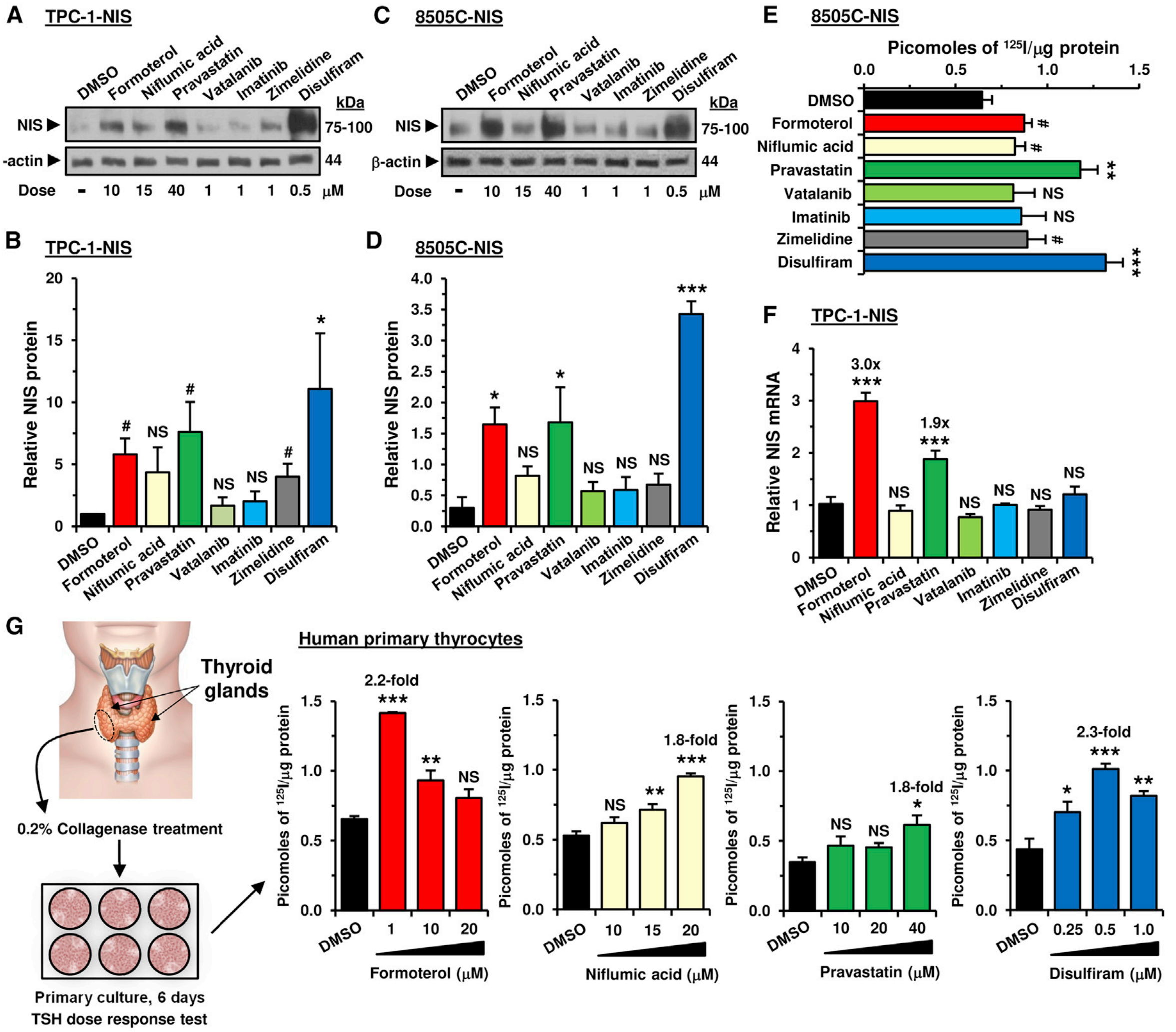


H

| Drug | EC50 (μM) | | Category | Targets |
|---|---|---|---|---|
| | TPC-1-NIS | Human Thyrocytes | | |
| Astemizole | 0.07 | N/A | Antihistamine | VCP |
| Clotrimazole | 0.16 | N/A | Antifungal | VCP |
| Vatalanib | 0.20 | 1.33 | Anticancer | Tyrosine kinase |
| Disulfiram | 0.24 | 0.25 | Antialcoholism | Autophagy, Proteasomal |
| Ebastine | 0.25 | N/A | Antihistamine | VCP |
| Imatinib | 0.28 | 0.21 | Anticancer | Tyrosine kinase |
| Zimelidine | 0.81 | 0.51 | Antidepressant | Serotonin reuptake |
| Formoterol | 0.84 | N/A | β(2) -agonist | Autophagy, Proteasomal |
| SAHA | 2.25 | 2.68 | Anticancer | HDAC |
| Niflumic acid | 6.24 | 11.17 | Anti-inflammatory | Iodide efflux |
| Pravastatin | 12.12 | 20.04 | Lipid-lowering | HMG-CoA reductase |
| Chloroquine | N/A | 10.14 | Antimalarial | Autophagy, Lysosomes, Vesicular Transport |
| Phenformin | 66.79 | N/A | Antidiabetic | Autophagy, mTOR |

I

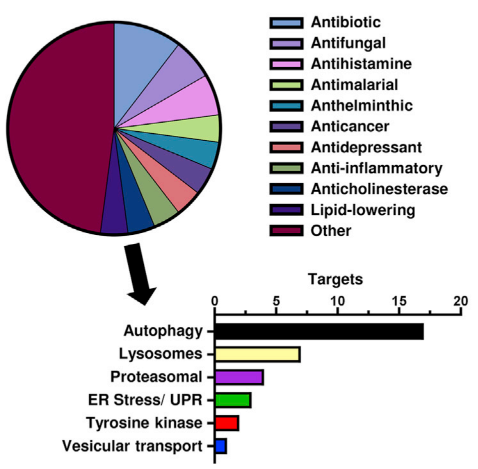


**Figure 2. Targeting multiple processes that regulate NIS to enhance radioiodide uptake**

(A) Western blot analysis of NIS expression levels in TPC-1-NIS cells treated with the indicated drugs for 24 h. Doses (μM) are indicated.

(B) Quantification of NIS protein levels in (A).

(C and D) Same as (A) and (B) but NIS expression levels in 8505C-NIS cells.

(E) Radioiodide uptake of 8505C-NIS cells treated with the indicated drugs for 24 h.

(F) Relative NIS mRNA expression levels in TPC-1-NIS cells treated with the indicated drug for 24 h.

(legend continued on next page)

eeyarestatin-1 (ES-1) and NMS-873, which reflected increases in NIS protein mediated via SAHA but also by the VCP inhibitors themselves (Figures S2A and S2B).

The ability of carebastine (CBT), the active metabolite of EBT (Fujii et al., 1997), to modulate NIS function has not been reported. Here, we found a dose-dependent effect of CBT on $^{125}$I uptake in TPC-1 stable cells (Figure 3E), with similar efficacy to its parent compound. To test whether the impact of CBT was simply a thyroid-specific phenomenon, we utilized breast cancer MDA-MB-231 cells lentivirally expressing NIS. CBT was similarly potent in stimulating radioiodide uptake, confirming that it targets a generic mechanism of NIS processing (Figure S2C).

Critically, CBT worked additively with SAHA to induce $^{125}$I uptake in parental TPC-1 cells which have low NIS expression typical of DTC cells (Figure 3F). Significantly greater radioiodide uptake was accompanied by the induction of NIS mRNA and protein by SAHA but not CBT (Figures 3G and S2D). Protein levels of VCP, a highly abundant cellular protein (Meyer and Weihl, 2014), were unaltered by either treatment (Figure S2E). In support, CBT worked additively with SAHA to induce radioiodide uptake in parental breast cancer MDA-MB-231 and MCF7 cells without affecting NIS mRNA or VCP protein (Figures S2F–S2K). Importantly, both SAHA and SAHA + CBT induced NIS protein in parental MCF7 cells (Figure S2L).

To determine to what extent the action of CBT and SAHA were dependent upon VCP, we next depleted VCP in TPC-1-NIS cells. Both VCP siRNA and SAHA increased $^{125}$I uptake, with a maximal effect when both were combined (Figure 3H). However, CBT was unable to induce $^{125}$I uptake when VCP was depleted, whereas SAHA retained its induction of NIS activity (Figures 3H and 3I), without any significant change in VCP protein levels (Figures 3I and S2M). In contrast, NIS mRNA levels were not altered by VCP siRNA or CBT but were altered by SAHA (Figure 3J). In control experiments, CBT did not affect siRNA ablation of VCP mRNA (Figure 3K), and VCP depletion similarly abrogated the CBT effect on NIS function in breast MDA-MB-231-NIS cells (Figures S2N–S2Q). Furthermore, in contrast to the well-established autophagy inhibitor bafA1, CBT did not affect LC3B-II or p62 levels (Figures 3L, S3A, and S3B), suggesting that it acts independently of autophagy.

Collectively, these data support the hypothesis that the additive effects on radioiodide uptake arise from SAHA's induction of NIS expression, and VCP inhibitor blockage of VCP-dependent proteasomal pathways, which control the subsequent protein processing of NIS.

## Autophagy inhibitors enhance radioiodide uptake in multiple cell types

We next sought to investigate the mechanism(s) underlying the significant impact of chloroquine and disulfiram on radioiodide uptake. Both drugs have been associated with modulating autophagy in cancer settings (Chude and Amaravadi, 2017; Zhang et al., 2019). First, we demonstrated that the autophagy inhibitor bafA1 enhanced NIS expression and $^{125}$I uptake in thyroid cells (Figures S3C–S3E), as well as increasing expression of autophagy markers (Figures 3L, S3A and S3B). Similarly, chloroquine induced LC3B-II, accompanied by a time-dependent increase in NIS protein in total cell lysates (Figure 4A), with radioiodide uptake peaking at 8 h post-treatment (Figures 4B and 4C). In contrast, maximal $^{125}$I uptake and NIS protein levels occurred later with disulfiram at 24 h (Figures S3F and S3G). Disulfiram also did not impact LC3B-II (Figure S3H), but increased p62 expression, which may be indicative of its ability to inhibit the VCP/proteasomal machinery (Lovborg et al., 2006; Skrott et al., 2017). Disulfiram also failed to impact the stringency of the NIS:VCP interaction using NanoBiT (Figure S3I) and retained the ability to enhance NIS function in VCP-ablated 8505C-NIS cells (Figures S3J and S3K), confirming that its effect on NIS is via targeting of VCP-independent proteasomal pathways.

We next progressed to determine whether combining drugs with distinct modes of action on NIS processing, such as chloroquine and disulfiram with SAHA, might give a robust increase in radioiodide uptake. Importantly, chloroquine doubled the impact of SAHA on $^{125}$I uptake in TPC-1-NIS cells (~8.3-fold; Figure 4D), without additional upregulation of NIS mRNA expression (Figure 4E). Virtually identical data were apparent in 8505C cells (Figure 4F). As a control, we inhibited BRAF$^{V600E}$ activity via vemurafenib, which approximately doubled radioiodide uptake in BRAF$^{V600E}$-mutant 8505C cells (Figure 4G). Chloroquine did not enhance the action of vemurafenib further, whereas co-treatment of chloroquine and SAHA resulted in a much more potent 5.6-fold effect. Again, the predominant mechanistic impact was induction of NIS protein expression (Figure 4H).

The impact of SAHA alone was relatively less effective in human primary thyrocytes (~1.6-fold increase) than in TPC-1 cells (Figure 4I), which likely reflects reduced epigenetic repression of NIS mRNA in non-transformed thyrocytes compared with transformed thyroid cells (Passon et al., 2012; Zhang et al., 2014). An important finding from our combination strategies, however, was that chloroquine and SAHA retained a robust and additive 4.3-fold effect on radioiodide uptake in primary cells, which was greater than chloroquine alone (Figure 4J). By comparison, there was no additive effect from combining disulfiram with SAHA or chloroquine (Figure 4K). The highest fold induction of radioiodide uptake from combinatorial drug screening in non-transformed primary thyroid cells came from chloroquine plus niflumic acid (7.3-fold; Figure 4L).

Full drug combination data (Table 1) revealed that the best overall strategies that enhanced radioiodide uptake across at least four different cell types were chloroquine + SAHA (5.3-fold), CBT + SAHA (9.6-fold), and EBT + SAHA (6.4-fold). The majority of drugs acted in cooperation with SAHA (7/8, 87.5%; $p < 0.05$

(G) Schematic (left) depicting the harvesting and culturing of human thyrocytes. Source: GoGraph. Radioiodide uptake of primary human thyrocytes cells treated with drugs at indicated doses for 24 h. See also Figure S1E and Table 1.

(H) Comparison of half-maximal effective concentration ($EC_{50}$) values in TPC-1-NIS cells and primary human thyrocytes.

(I) Categorization of the top 50 drugs (pie chart) identified in the primary high-throughput drug screen. The graph (below) summarizes known or putative drug targets. See also Table S1.

Data presented as mean ± SEM, n = 3–6, one-way ANOVA followed by Dunnett's post hoc test (NS, not significant; *$p < 0.05$, **$p < 0.01$, ***$p < 0.001$) or unpaired two-tailed t test ($^{\#}p < 0.05$).

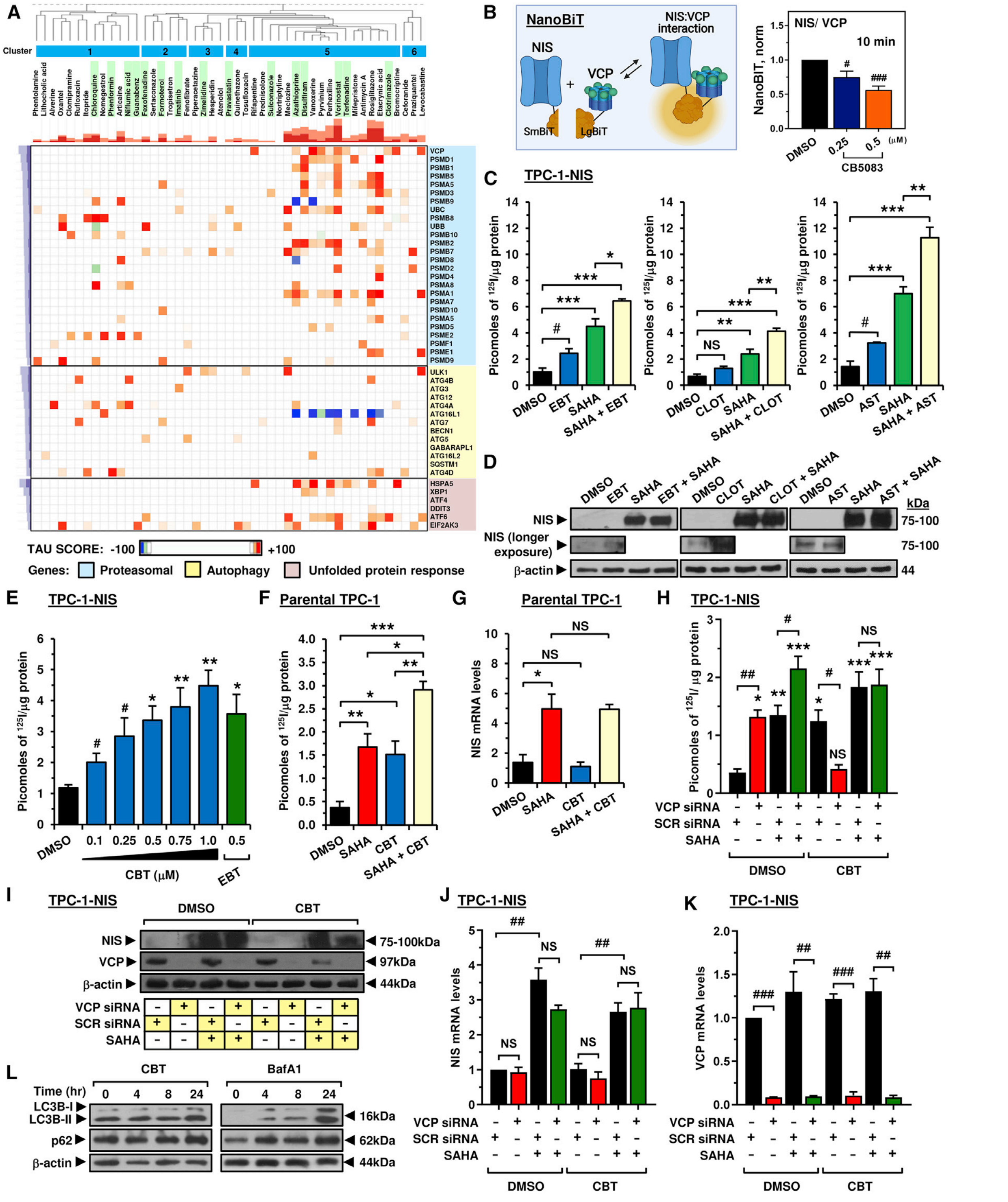


**Figure 3. Carebastine inhibits VCP to enhance NIS function and radioiodide uptake**

(A) Connectivity Map (L1000 assay) hierarchical cluster analysis comparing the transcriptional signatures of 47 drug treatments with 45 genetic perturbations. Blue, major clusters; green, validated drugs.

(B) NanoBiT evaluation of protein:protein interaction between VCP and NIS (left). Normalized NanoBiT assay results at 10 min after addition of Nano-Glo live cell assay solution to HeLa cells treated with VCP inhibitor CB5083 for 24 h (right, n = 4).

(C and D) Radioiodide uptake (C) and relative NIS protein levels (D) in TPC-1-NIS cells treated with VCP inhibitors (ebastine [EBT], clotrimazole [CLOT], and astemizole [AST]), either alone or in combination with SAHA.

(E) Radioiodide uptake of TPC-1-NIS cells treated with carebastine (CBT) at indicated doses versus EBT. See also Figure S2C.

(legend continued on next page)

CellPress
OPEN ACCESS

versus SAHA alone), with most sharing a common target in modulating NIS processing (i.e., ERAD/ubiquitin-proteasome and autophagy-lysosome pathways). Control sodium perchlorate treatments clearly demonstrated that radioiodide uptake was NIS specific in drug-treated TPC-1-NIS cells (Figure 4M) and primary thyrocytes (Figure 4N).

### Clinically relevant proteostasis drug targets are associated with recurrence in RAI-treated patients

Having identified drug strategies that robustly enhance $^{125}$I uptake in thyroid cells, we next appraised TCGA (i.e., THCA, n = 501) and other GEO datasets to investigate the clinical relevance of core proteostasis target genes linked to recurrence in papillary thyroid cancer (PTC) patients associated with RAI treatment. First, we investigated 142 core proteostasis genes divided into four functional categories (Table S4), as many of our candidate drugs target different arms of the protein homeostasis network, including proteasomal degradation and autophagy. Of significance, expression of the majority of core proteostasis genes (102/142 genes) were dysregulated in PTC compared with normal tissue (Figure 5A). A greater proportion of proteasomal genes were significantly upregulated (29/45 genes; $p < 0.05$; Figure 5A), whereas unfolded protein response genes were generally downregulated (5/6 genes, $p < 0.01$).

Genetic drivers in PTC have well-characterized signaling consequences that have been categorized into BRAF-like and RAS-like according to distinct gene signatures (Cancer Genome Atlas Research, 2014). Subsequent hierarchical cluster and expression analyses of the 142 core proteostasis genes in PTC with BRAF- or RAS-like signatures highlighted distinctive patterns (Figure 5B), as well as specific markers associated with recurrence and/or RAI treatment (Figure S4A). Of most importance was the identification of 13 core proteostasis genes significantly dysregulated in RAI-treated patients with recurrent BRAF-like PTC compared with non-recurrent controls (Figures 5C and S4B). Disease classification also showed that a BRAF-like signature and RAI treatment are both highly indicative of significantly more advanced PTC (Figure S4C).

All 13 core proteostasis genes were associated with a significant reduction in disease-free survival following stratification of RAI-treated PTC into subgroups of high versus low tumoral expression with optimal cutoffs determined by receiver operating characteristic analysis (Figures 5D and S5A–S5C). Stratification of PTC using percentile cutoff values further validated the influence of each proteostasis gene on recurrence, especially with higher VCP expression (Figures 5E and S5D). Univariate Cox regression analysis (Figure S5B) indicated that all 13 proteostasis genes significantly correlated with an increased risk of recurrence in RAI-treated BRAF-like PTC ($p < 0.05$; Figure 5F; Table S5) and were therefore all considered to construct the prognosis risk model.

Subsequently risk scores were calculated using multivariate Cox regression coefficient and fragments per kilobase transcript per million mapped reads expression values (Figure S6A). Importantly, a higher area under the curve (AUC) of 0.842 (Figure 5G) indicated a greater prediction effect for the 13-gene-based risk score compared with individual genes (AUC = 0.652–0.726; Figure S5A). In agreement, stratification into risk groups demonstrated that patients at high risk had a significantly worse prognosis (hazard ratio = 35.862; 95% confidence interval: 4.81–267.405; Figures 5H and S6B) at significance levels several orders of magnitude greater than individual genes (Figure S5C). By comparison, there was no difference in the prognosis of non-RAI-treated patients stratified into risk groups (Figure 5I).

After controlling for age, gender, disease stage, tumor stage, and node status, multivariate analysis showed that the 13-gene risk score remained the sole independent predictive factor for the entire group of THCA patients, as well as for the RAI-treated, BRAF-like and RAI-treated cohorts (Tables S6 and S7). The performance of the 13-gene risk score classifier was also superior to VCP, which did not retain its predictive value in other THCA cohorts (Table S7). Consistent with TCGA, there was extensive dysregulation of proteostasis genes in PTC with BRAF or RAS mutations in the independent GEO dataset GSE27155 (Figures S6C–S6F), as well as the primary anaplastic GSE76039 dataset (Figure S7A). Critically, the 13 proteostasis gene set lacked any strong association with aggressive tumor types with only heat shock 70 kDa protein 5 (*HSPA5*) common in 3 datasets (Figures S7A–S7C), further supporting that its predictive value would be defined predominately by molecular influences affecting RAI therapy. Subsequent validation also showed a greater predictive value in RAI-treated PTC against seven biomarkers of thyroid cancer recurrence (Cheng et al., 2011; Khoo et al., 2002) (Figures S7D and S7E).

Collectively, we have identified core proteostasis genes that are significantly dysregulated in PTC and associated with poorer survival characteristics, especially in patients treated with radioiodide. In support of this, our drug screening data particularly highlight mechanisms of proteostasis as being critical to NIS function. We therefore hypothesize that dysregulation of specific proteostasis genes in PTC leads to abrogation of NIS localization at the PM, as demonstrated recently for VCP and ADP-ribosylation factor 4 (Fletcher et al., 2020). Based on our collective drug screening, experimental manipulations, and clinical gene expression analyses, we therefore propose a model for the key targetable steps of intracellular processing of NIS expression and function in PTC (Figure 6).

## DISCUSSION

New approaches to better understand and identify key targetable processes that govern NIS function are urgently needed

(F) Same as (E) but parental TPC-1 cells treated with CBT and/or SAHA. See also Figures S2F and S2I.
(G) Relative NIS mRNA levels in parental TPC-1 cells as described in (F).
(H) Radioiodide uptake of TPC-1-NIS cells following VCP siRNA depletion and treatment with CBT and/or SAHA.
(I–K) Relative NIS protein (I), NIS mRNA (J), and VCP mRNA levels (K) in TPC-1-NIS cells as described in (H). See also Figure S2M.
(L) Western blot analysis of LC3B-I, LC3B-II, and p62 protein levels in TPC-1-NIS cells treated with CBT (left) or bafA1 (right). See also Figures S3A and S3B.
Data presented as mean ± SEM, n = 3, one-way ANOVA followed by Tukey's (C, F, and G) or Dunnett's (E and H) post hoc test (NS, not significant; $^{*}p < 0.05$, $^{**}p < 0.01$, $^{***}p < 0.001$) or unpaired two-tailed t test ($^{\#}p < 0.05$, $^{\#\#}p < 0.01$, $^{\#\#\#}p < 0.001$).

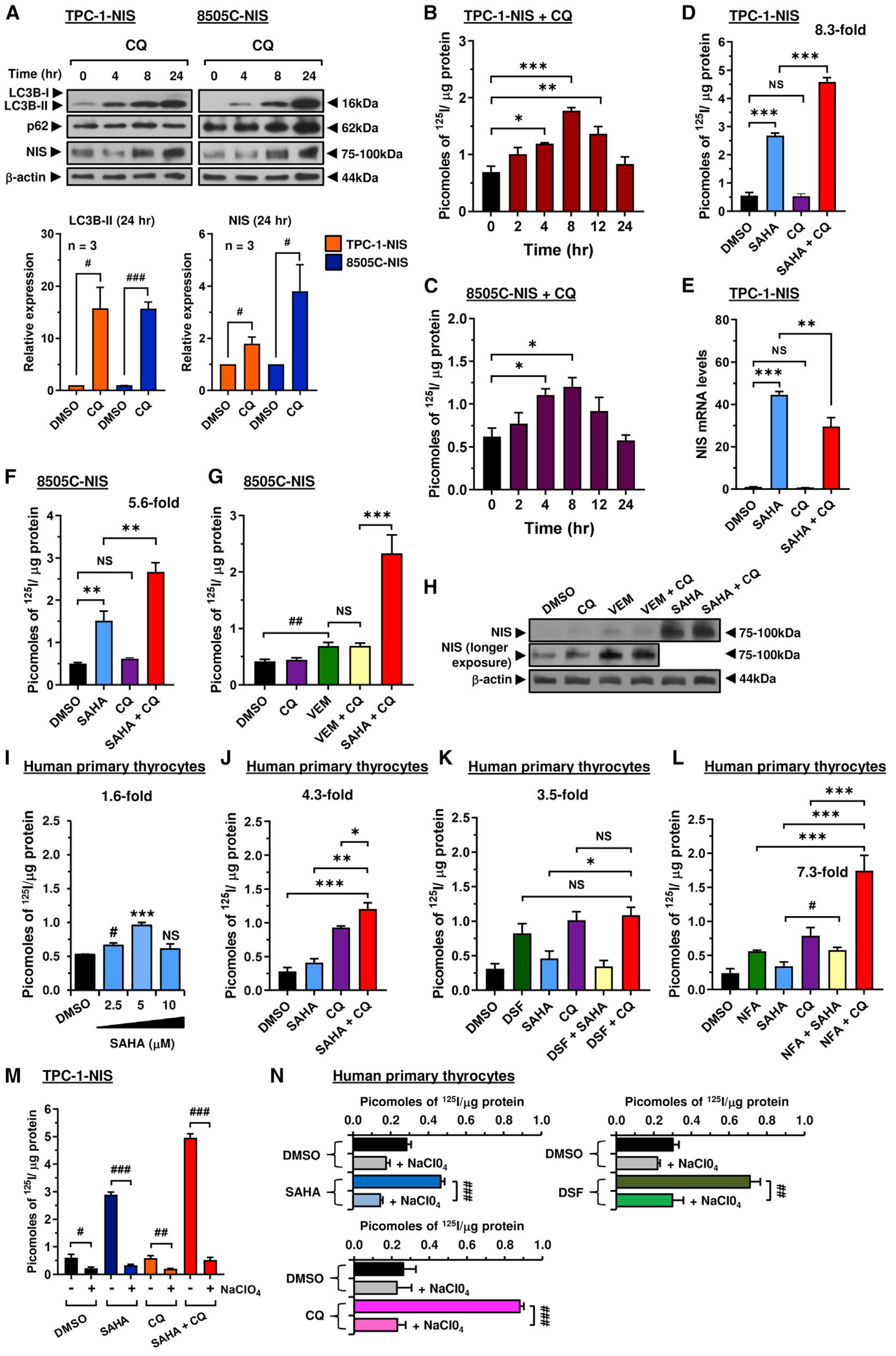


**Figure 4. Drug combinations targeting non-canonical pathways augment NIS function**

(A) Western blot analysis of LC3B-I, LC3B-II, p62, and NIS protein levels in TPC-1-NIS or 8505C-NIS cells treated with chloroquine (CQ).

(B and C) Radioiodide uptake in TPC-1-NIS (B) or 8505C-NIS (C) cells treated with CQ at different time points.

(legend continued on next page)

to improve the efficacy of radioiodide therapy and diminish recurrence of thyroid cancer. HTS of NIS function has been reported before, but primarily for toxicology purposes. One previous study used HTS of the NIS promoter to detect drugs that increased NIS expression (Oh et al., 2018). However, our study directly "drugged" NIS function. Of particular significance, we illustrate that pathways entirely separate to the canonical MAPK regulation of NIS exist, which can be drugged to enhance radioiodide uptake.

A significant advantage of HTS was that it enabled the objective evaluation of drugs targeting multiple cellular processes that regulate NIS. For example, we identified that formoterol and pravastatin directly increase NIS mRNA expression, while carebastine, a second-generation antihistamine, inhibited VCP to enhance radioiodide uptake. The importance of VCP and other critical components of proteostasis pathways was further highlighted by the abundance of putative proteasomal inhibitors identified by HTS that were subsequently validated to enhance radioiodide uptake. For instance, disulfiram—a prominent drug in our study—inhibits the proteasome (Lovborg et al., 2006) and has been associated with the accumulation of ubiquitylated proteins in esophageal squamous cell carcinoma (Jivan et al., 2018). We thus propose that VCP and related proteasomal pathways represent key targetable processes to modulate NIS function in thyroid cancer. In agreement, the appraisal of TCGA and GEO datasets revealed profound dysregulation of core proteostasis genes, including those involved in proteasomal degradation. Together, these findings support our hypothesis that increased expression of VCP and multiple components of the proteasome in PTC enhance the degradation of NIS, leading to reduced radioiodide uptake.

Autophagy has been associated with NIS function (Chai et al., 2019), although the precise mechanism of how this might regulate NIS remains obscure. In thyroid cancers, markers of increased autophagy correlate with higher levels of plasma membranous NIS, and better clinical outcome (Plantinga et al., 2016). Our data clearly show that well-established inhibitors of autophagy, such as chloroquine and bafilomycin A, induce significant increases in iodide uptake in transformed and untransformed thyroid cells, accompanied by increased classical autophagy markers, suggesting that autophagy is critical to the central processing of NIS. VCP has been reported to be essential for autophagosome maturation (Tresse et al., 2010), and hence it is possible that autophagosome maturation or processes downstream of it, rather than increased autophagy per se, are the more pertinent pathways to NIS druggability.

An alternative pathway might involve endosomes recycling NIS away from the PM, a process described previously by ourselves and others (Fletcher et al., 2020; Smith et al., 2009, 2013). Endosomes are known to fuse with autophagosomes as part of lysosomal degradation. Autophagy selectively degrades targets and contributes to intracellular homeostasis (Kawabata and Yoshimori, 2016). Drugs that interfere with autophagosome initiation, maturation, or fusion with lysosomes, such as chloroquine, might all potentially result in NIS-bearing endosomes not being degraded, and hence subsequently increased intracellular NIS protein levels. The ability of autophagy inhibition to modulate trafficking of NIS-containing endosomes and the possibility of NIS recycling to the PM to expedite iodide uptake warrants further investigation.

New drug strategies, such as combining BRAF or MEK inhibitors with pan-PI3K inhibitors, are beginning to show pre-clinical promise in thyroid cancer (Nagarajah et al., 2016). However, we propose that drug combinations targeting pathways outside those canonical signaling pathways may be capable of augmenting NIS expression, processing, and trafficking to the PM to boost the efficacy of radioiodide therapy. We identified at least three drug combinations (i.e., ebastine + SAHA, carebastine + SAHA, or chloroquine + SAHA) which target non-canonical pathways and represent realistic drug strategies in thyroid cancer with favorable side effect profiles. Effective drug doses for ebastine, carebastine, and SAHA to promote radioiodide uptake were comparable with mean plasma concentrations that can be safely achieved in patients (Dickson et al., 2011; Yamaguchi et al., 1994). The maximal effective concentration of chloroquine in cells was relatively high at 50 μM, with an $EC_{50}$ of 10.14 μM in human primary thyrocytes. Given that the concentration of chloroquine in tissues can be many times greater than in plasma due to accumulation in cell structures (Adelusi and Salako, 1982), it will be important to determine if sufficient distribution of chloroquine in thyroid tissue can be achieved using standard doses. Further studies will be needed to investigate the *in vivo* efficacy of these drugs in combination to determine the precise timing and order of drug administration to maximize recovery of radioiodide uptake. In agreement with our findings with carebastine in MDA-MB-231 and MCF7 cells, these drug strategies also have potential for broader application to boost NIS function in non-thyroidal settings, including the radioiodide treatment for breast cancer in which NIS expression is typically induced in ~70%–80% of cases (Tazebay et al., 2000).

Despite extensive studies, there are no molecular biomarkers currently in clinical use for predicting recurrence. Here, an important clinical observation was the striking correlation with recurrence in PTC and the dysregulation of core proteostasis genes, including those involved in proteasomal degradation and autophagy. Via bioinformatic analyses we identified the most clinically relevant core proteostasis genes associated with a poor radioiodide response in recurrent thyroid cancer and

(D and E) Radioiodide uptake (D) and relative NIS mRNA levels (E) in TPC-1-NIS cells treated with CQ alone or in combination with SAHA.
(F) Same as (D) but radioiodide uptake in 8505C-NIS cells.
(G and H) Radioiodide uptake (G) and relative NIS protein levels (H) in 8505C-NIS cells treated with vemurafenib (VEM) + CQ versus SAHA + CQ.
(I–L) Radioiodide uptake in human primary thyrocytes treated with drugs alone (disulfiram [DSF], niflumic acid [NFA], SAHA, and CQ) or in combination. (M) Radioiodide uptake in TPC-1-NIS cells treated with drugs as indicated for 24 h, and then incubated with 100 μM sodium perchlorate ($NaClO_4$) for 1 h before addition of $^{125}I$.
(N) Same as (M) but radioiodide uptake in human primary thyrocytes.
Data presented as mean ± SEM, n = 3–5, one-way ANOVA followed by Dunnett's (B, C, and I) or Tukey's (D–G, J–L) post hoc test (NS, not significant; *$p < 0.05$, **$p < 0.01$, ***$p < 0.001$) or unpaired two-tailed t test ($^{\#}p < 0.05$, $^{\#\#}p < 0.01$, $^{\#\#\#}p < 0.001$).

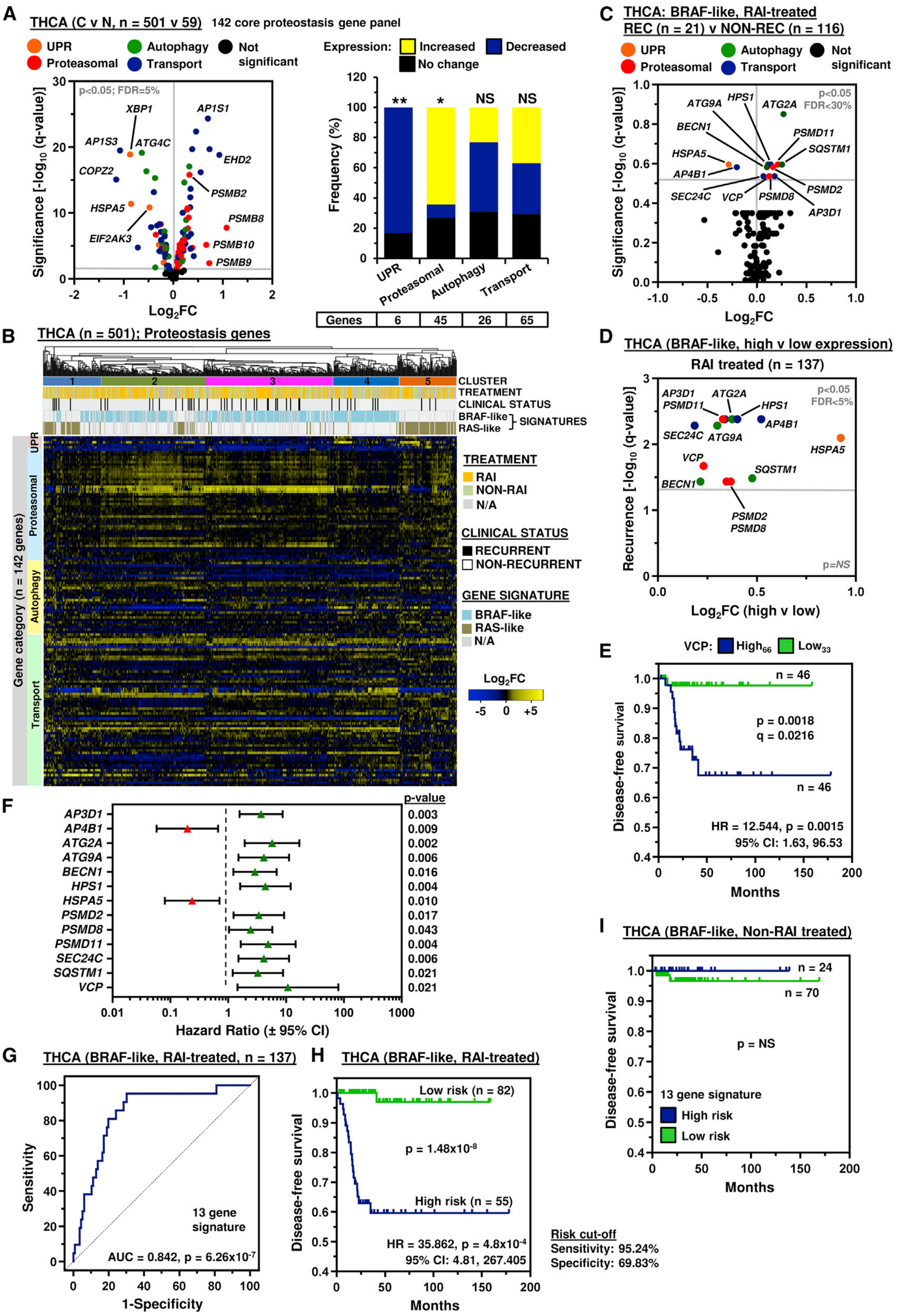


**Figure 5. A 13 proteostasis gene risk score classifier is predictive of thyroid cancer recurrence**

(A) Volcano plot comparing $\log_2$FC with q value (–log base 10) for 142 core proteostasis genes in the THCA cohort (C versus N). Gene categories indicated by colored spots. (Right) Stacked bar chart indicates frequency of altered expression; Fisher's exact test (NS, not significant; *p < 0.05, **p < 0.01).

(legend continued on next page)

constructed a 13-gene predictive risk model. Critically, the 13-gene risk score classifier yielded higher specificity and sensitivity than single gene biomarkers, as well as being the sole independent predictive factor for recurrence. Based on these observations, we envisage that the 13 proteostasis gene classifier represents a promising biomarker for predicting thyroid cancer recurrence, especially in more aggressive thyroid cancers requiring radioiodide. Further validation will now be required using larger independent clinical datasets, which are not yet available in public data repositories.

Overall, we report a large-scale screen aimed at enhancing NIS activity and identifying cellular processes that impact radioiodide uptake. Our findings demonstrate that cellular processing of NIS involves VCP and suggest that the proteasome is an integral regulatory pathway of NIS activity given the marked dysregulation of proteostasis genes in PTC. Notably, combinatorial drug strategies targeting proteostasis networks and HDAC revealed that significant induction of NIS function is routinely possible *in vitro*, with translatable potential to address the lack of therapeutic options for patients treated with radioiodide who typically have poorer clinical outcomes.

## SIGNIFICANCE

**The sodium iodide symporter (NIS) is the sole conduit for cellular iodide uptake. Dysregulation of NIS function is common in thyroid cancer, significantly impacting therapeutic radioiodide ablation of tumor and metastatic cells. Here, we summarize our findings on the use of NIS as a drug target to both understand the full gamut of mechanisms that underpin NIS function and to transform the efficacy of radioiodide treatment. Our study yielded critical insights into cellular processes central to NIS regulation, including proteasomal degradation and autophagy. Dose-dependent increases in drug effects were apparent across multiple cancer cell models, as well as human primary thyrocytes, implying that proteostasis pathways are central to the innate control of NIS function. Exploiting these mechanistic insights, several drugs—which target pathways entirely separate to the canonical MAPK regulation of NIS—gave robust increases in radioiodide uptake when combined. There are currently no molecular biomarkers in clinical use for predicting thyroid cancer recurrence. We also show significant perturbation of proteostasis genes in thyroid cancer and identify a 13-proteostasis gene risk score classifier as an independent predictor of recurrence in radioiodide-treated patients. Collectively, we propose a model for the targetable steps of intracellular processing of NIS function, with translatable potential to address the current lack of clinical options for those with aggressive thyroid cancer. This work will be of relevance to a broad readership in academia, medicine, and the pharmaceutical industry given the potential application of our findings across a wide-ranging disease spectrum.**

## STAR★METHODS

Detailed methods are provided in the online version of this paper and include the following:

- KEY RESOURCES TABLE
- RESOURCE AVAILABILITY
  - Lead contact
  - Materials availability
  - Data and code availability
- EXPERIMENTAL MODEL AND SUBJECT DETAILS
  - Cell lines
  - Human primary thyroid tissue
- METHOD DETAILS
  - Drug screening strategy
  - Inhibitors/ drugs used
  - Cell viability assay
  - Determination of ΔYFP values
  - Radioiodide uptake assay
  - Immunoblots
  - siRNA transfections
  - qPCR
  - Plasmid transfections
  - NanoBiT cell-based assays
  - TCGA and GEO datasets
  - Connectivity map analysis
- QUANTIFICATION AND STATISTICAL ANALYSIS

## SUPPLEMENTAL INFORMATION

Supplemental information can be found online at https://doi.org/10.1016/j.chembiol.2021.07.016.

## ACKNOWLEDGMENTS

This work was supported by the MRC (MR/P000509/1), the Wellcome Trust (RG_05–052), Get A-Head, and The University of Birmingham. We acknowledge the contribution of the Human Biomaterials Resource Center (University of Birmingham) and thank Evotec (Hamburg, Germany) for help with the Opera Phenix system. We also kindly acknowledge Prof Alan Verkman and Dr Peter Haggie (UCSF) for providing the YFP constructs, and Dr Caroline Gorvin (University of Birmingham) for providing NanoBiT plasmids.

(B) Hierarchical cluster analysis of the THCA cohort (n = 501) based on $log_2$FC (C versus N) of 142 core proteostasis genes. See also Figure S4A.
(C) Volcano plot comparing $log_2$FC for 142 proteostasis genes in the BRAF-like, RAI-treated THCA cohort (recurrent [REC] versus non-recurrent [NON-REC]). See also Figure S4B.
(D) Volcano plot illustrating $log_2$FC compared with q value (–log base 10) for recurrence in the BRAF-like, RAI-treated THCA cohort (high versus low tumoral expression) for 13 proteostasis genes.
(E) Disease-free survival of the BRAF-like, RAI-treated THCA cohort between high (>66th percentile) and low (<33rd percentile) tumoral VCP expression; log rank test. See also Figure S5D.
(F) Hazard ratio (HR) ± 95% confidence interval (CI) for the BRAF-like, RAI-treated THCA cohort stratified using optimal expression cutoff values for 13 proteostasis genes (univariate Cox regression analysis). See also Table S5.
(G–I) Receiver operating characteristic analysis (G) and Kaplan-Meier curve of the 13-gene risk score signature in the BRAF-like, RAI-treated (H), or non-RAI-treated (I) THCA cohort. See also Figure S6B.

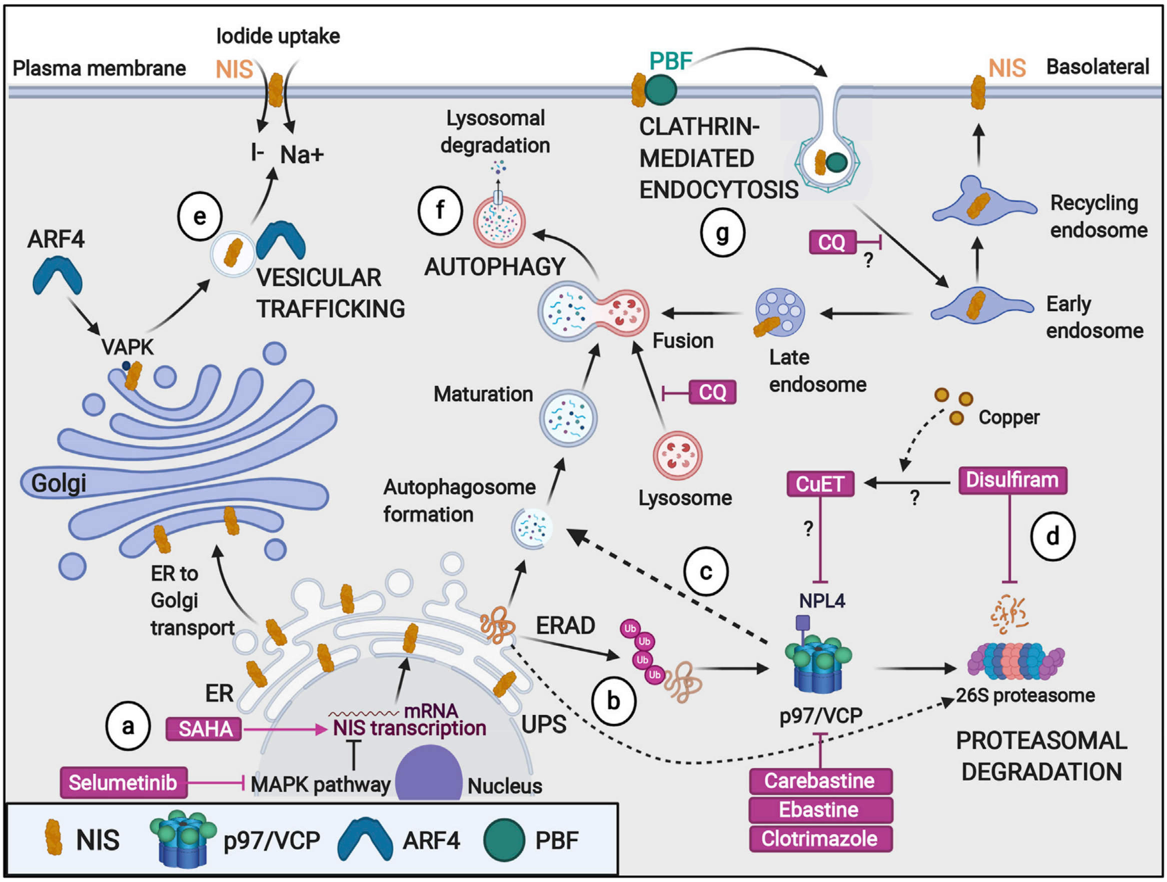


**Figure 6. Putative model of key targetable processes to enhance NIS function and radioiodide therapy**

NIS maintains a fine balance between protein synthesis, folding, assembly, trafficking, and degradation.

(A) The HDAC inhibitor SAHA and MEK inhibitor selumetinib stimulate transcriptional expression of NIS. Surveillance pathways target misfolded NIS protein for ER-associated degradation (ERAD), as well as performing homeostatic regulation of correctly folded NIS protein production (UPR).

(B and C) p97/VCP is a critical component of (B) ubiquitin-proteasome and (C) autophagy-lysosome pathways.

(D) Disulfiram inhibits proteasomal degradation and autophagy.

(E) Vesicular trafficking (e.g., ADP-ribosylation factor 4 [ARF4]) promotes transport of NIS to the PM where it is active.

(F) Late endosomes merge with autophagosomes prior to fusing with lysosomes for degradation of protein cargoes. Chloroquine inhibits the fusion of autophagosomes and lysosomes, and components of the proteasome.

(G) NIS is internalized away from the PM (e.g., PBF) in a clathrin-dependent process, which diminishes radioiodide uptake. Created with BioRender.com.

## AUTHOR CONTRIBUTIONS

Conceptualization, M.L.R., K. Brookes, V.E.S., and C.J.M.; methodology, M.L.R., K. Brookes, C.E.M.T., A.F., J.R.M.W., and L.J.A.; investigation, M.L.R., K. Brookes, C.E.M.T., A.F., H.R.N., M.A., R.K., P.B.d.S., and L.Z.; resources, J.R.M.W. and L.J.A.; formal analysis, M.L.R., K. Brookes, A.F., and M.J.C.; visualization, M.L.R. and K. Brookes; writing – original draft, M.L.R., K. Brookes, and C.J.M.; writing – review & editing, M.L.R. and C.J.M.; funding acquisition, K. Boelaert, V.E.S., and C.J.M.; supervision, V.E.S. and C.J.M.; project administration, C.J.M.

## DECLARATION OF INTERESTS

The authors declare no competing interests.

Received: February 25, 2021
Revised: May 17, 2021
Accepted: July 21, 2021
Published: September 13, 2021

## REFERENCES

Adelusi, S.A., and Salako, L.A. (1982). Kinetics of the distribution and elimination of chloroquine in the rat. Gen. Pharmacol. *13*, 433–437.

Arriagada, A.A., Albornoz, E., Cecilia Opazo, M., Becerra, A., Vidal, G., Fardella, C., Michea, L., Carrasco, N., Simon, F., Elorza, A., et al. (2015). Excess iodide induces an acute inhibition of the sodium/iodide symporter in thyroid rat cells by increasing reactive oxygen species. Endocrinology *156*, 1540–1551.

Azouzi, N., Cailloux, J., Cazarin, J.M., Knauf, J.A., Cracchiolo, J., Al Ghuzla, n.A., Hartl, D., Polak, M., Carré, A., El Mzibri, M., et al. (2017). NADPH oxidase NOX4 is a critical, mediator of BRAF$^{V600E}$-induced downregulation of the sodium/iodide symporter in papillary thyroid carcinomas. Antioxid Redox Signal *26*, 864–877.

Barrett, T., Wilhite, S.E., Ledoux, P., Evangelista, C., Kim, I.F., Tomashevsky, M., Marshall, K.A., Phillippy, K.H., Sherman, P.M., Holko, M., et al. (2013). NCBI GEO: archive for functional genomics data sets–update. Nucleic Acids Res. *41*, D991–D995.

CellPress
OPEN ACCESS

Buckalew, A.R., Wang, J., Murr, A.S., Deisenroth, C., Stewart, W.M., Stoker, T.E., and Laws, S.C. (2020). Evaluation of potential sodium-iodide symporter (NIS) inhibitors using a secondary Fischer rat thyroid follicular cell (FRTL-5) radioactive iodide uptake (RAIU) assay. Arch. Toxicol. *94*, 873–885.

Cancer Genome Atlas Research. (2014). Integrated genomic characterization of papillary thyroid carcinoma. Cell *159*, 676–690.

Cerami, E., Gao, J., Dogrusoz, U., Gross, B.E., Sumer, S.O., Aksoy, B.A., Jacobsen, A., Byrne, C.J., Heuer, M.L., Larsson, E., et al. (2012). The cBio cancer genomics portal: an open platform for exploring multidimensional cancer genomics data. Cancer Discov. *2*, 401–404.

Chai, W., Ye, F., Zeng, L., Li, Y., and Yang, L. (2019). HMGB1-mediated autophagy regulates sodium/iodide symporter protein degradation in thyroid cancer cells. J. Exp. Clin. Cancer Res. *38*, 325.

Cheng, S., Serra, S., Mercado, M., Ezzat, S., and Asa, S.L. (2011). A high-throughput proteomic approach provides distinct signatures for thyroid cancer behavior. Clin. Cancer Res. *17*, 2385–2394.

Cheng, W., Liu, R., Zhu, G., Wang, H., and Xing, M. (2016). Robust thyroid gene expression and radioiodine uptake induced by simultaneous suppression of BRAF V600E and histone deacetylase in thyroid cancer cells. J. Clin. Endocrinol. Metab. *101*, 962–971.

Chude, C.I., and Amaravadi, R.K. (2017). Targeting autophagy in cancer: update on clinical trials and novel inhibitors. Int. J. Mol. Sci. *18*, 1279.

Dai, G., Levy, O., and Carrasco, N. (1996). Cloning and characterization of the thyroid iodide transporter. Nature *379*, 458–460.

Dickson, M.A., Rathkopf, D.E., Carvajal, R.D., Grant, S., Roberts, J.D., Reid, J.M., Ames, M.M., McGovern, R.M., Lefkowitz, R.A., Gonen, M., et al. (2011). A phase I pharmacokinetic study of pulse-dose vorinostat with flavopiridol in solid tumors. Invest. New Drugs *29*, 1004–1012.

Dunn, L.A., Sherman, E.J., Baxi, S.S., Tchekmedyian, V., Grewal, R.K., Larson, S.M., Pentlow, K.S., Haque, S., Tuttle, R.M., Sabra, M.M., et al. (2019). Vemurafenib redifferentiation of BRAF mutant, RAI-refractory thyroid cancers. J. Clin. Endocrinol. Metab. *104*, 1417–1428.

Ferlay, J., Colombet, M., Soerjomataram, I., Mathers, C., Parkin, D.M., Pineros, M., Znaor, A., and Bray, F. (2019). Estimating the global cancer incidence and mortality in 2018: GLOBOCAN sources and methods. Int. J. Cancer *144*, 1941–1953.

Fletcher, A., Read, M.L., Thornton, C.E.M., Larner, D.P., Poole, V.L., Brookes, K., Nieto, H.R., Alshahrani, M., Thompson, R.J., Lavery, G.G., et al. (2020). Targeting novel sodium iodide symporter interactors ADP-ribosylation factor 4 and valosin-containing protein enhances radioiodine uptake. Cancer Res. *80*, 102–115.

Fujii, T., Matsumoto, S., Hatoyama, T., and Miyazaki, H. (1997). Studies on the first-pass metabolism of ebastine in rats. Arzneimittelforschung *47*, 949–953.

Gao, J., Aksoy, B.A., Dogrusoz, U., Dresdner, G., Gross, B., Sumer, S.O., Sun, Y., Jacobsen, A., Sinha, R., Larsson, E., et al. (2013). Integrative analysis of complex cancer genomics and clinical profiles using the cBioPortal. Sci. Signal *6*, 11. https://doi.org/10.1126/scisignal.2004088.

Giordano, T.J., Kuick, R., Thomas, D.G., Misek, D.E., Vinco, M., Sanders, D., Zhu, Z., Ciampi, R., Roh, M., Shedden, K., et al. (2005). Molecular classification of papillary thyroid carcinoma: distinct BRAF, RAS, and RET/PTC mutation-specific gene expression profiles discovered by DNA microarray analysis. Oncogene *24*, 6646–6656.

Grossman, R.L., Heath, A.P., Ferretti, V., Varmus, H.E., Lowy, D.R., Kibbe, W.A., and Staudt, L.M. (2016). Toward a shared vision for cancer genomic data. N. Engl. J. Med. *375*, 1109–1112.

Hallinger, D.R., Murr, A.S., Buckalew, A.R., Simmons, S.O., Stoker, T.E., and Laws, S.C. (2017). Development of a screening approach to detect thyroid disrupting chemicals that inhibit the human sodium iodide symporter (NIS). Toxicol. In Vitro *40*, 66–78.

Ho, A.L., Grewal, R.K., Leboeuf, R., Sherman, E.J., Pfister, D.G., Deandreis, D., Pentlow, K.S., Zanzonico, P.B., Haque, S., Gavane, S., et al. (2013). Selumetinib-enhanced radioiodine uptake in advanced thyroid cancer. N. Engl. J. Med. *368*, 623–632.

Jivan, R., Peres, J., Damelin, L.H., Wadee, R., Veale, R.B., Prince, S., and Mavri-Damelin, D. (2018). Disulfiram with or without metformin inhibits oesophageal squamous cell carcinoma in vivo. Cancer Lett. *417*, 1–10.

Kawabata, T., and Yoshimori, T. (2016). Beyond starvation: an update on the autophagic machinery and its functions. J. Mol. Cell Cardiol *95*, 2–10.

Kelly, W.K., O'Connor, O.A., Krug, L.M., Chiao, J.H., Heaney, M., Curley, T., MacGregore-Cortelli, B., Tong, W., Secrist, J.P., Schwartz, L., et al. (2005). Phase I study of an oral histone deacetylase inhibitor, suberoylanilide hydroxamic acid, in patients with advanced cancer. J. Clin. Oncol. *23*, 3923–3931.

Khoo, M.L., Freeman, J.L., Witterick, I.J., Irish, J.C., Rotstein, L.E., Gullane, P.J., and Asa, S.L. (2002). Underexpression of p27/Kip in thyroid papillary microcarcinomas with gross metastatic disease. Arch. Otolaryngol. Head Neck Surg. *128*, 253–257.

Kitazono, M., Robey, R., Zhan, Z., Sarlis, N.J., Skarulis, M.C., Aikou, T., Bates, S., and Fojo, T. (2001). Low concentrations of the histone deacetylase inhibitor, depsipeptide (FR901228), increase expression of the Na(+)/I(–) symporter and iodine accumulation in poorly differentiated thyroid carcinoma cells. J. Clin. Endocrinol. Metab. *86*, 3430–3435.

Knauf, J.A., Sartor, M.A., Medvedovic, M., Lundsmith, E., Ryder, M., Salzano, M., Nikiforov, Y.E., Giordano, T.J., Ghossein, R.A., and Fagin, J.A. (2011). Progression of BRAF-induced thyroid cancer is associated with epithelial-mesenchymal transition requiring concomitant MAP kinase and TGFbeta signaling. Oncogene *30*, 3153–3162.

Lamb, J., Crawford, E.D., Peck, D., Modell, J.W., Blat, I.C., Wrobel, M.J., Lerner, J., Brunet, J.P., Subramanian, A., Ross, K.N., et al. (2006). The Connectivity Map: using gene-expression signatures to connect small molecules, genes, and disease. Science *313*, 1929–1935.

Landa, I., Ibrahimpasic, T., Boucai, L., Sinha, R., Knauf, J.A., Shah, R.H., Dogan, S., Ricarte-Filho, J.C., Krishnamoorthy, G.P., Xu, B., et al. (2016). Genomic and transcriptomic hallmarks of poorly differentiated and anaplastic thyroid cancers. J. Clin. Invest. *126*, 1052–1066.

Landa, I., Pozdeyev, N., Korch, C., Marlow, L.A., Smallridge, R.C., Copland, J.A., Henderson, Y.C., Lai, S.Y., Clayman, G.L., Onoda, N., et al. (2019). Comprehensive genetic characterization of human thyroid cancer cell lines: a validated panel for preclinical studies. Clin. Cancer Res. *25*, 3141–3151.

Lirov, R., Worden, F.P., and Cohen, M.S. (2017). The treatment of advanced thyroid cancer in the age of novel targeted therapies. Drugs *77*, 733–745.

Lovborg, H., Oberg, F., Rickardson, L., Gullbo, J., Nygren, P., and Larsson, R. (2006). Inhibition of proteasome activity, nuclear factor-kappaB translocation and cell survival by the antialcoholism drug disulfiram. Int. J. Cancer *118*, 1577–1580.

Luo, S., Huang, G., Wang, Z., Wan, Z., Chen, H., Liao, D., Chen, C., Li, H., Li, B., Chen, L., et al. (2015). Niflumic acid exhibits anti-tumor activity in nasopharyngeal carcinoma cells through affecting the expression of ERK1/2 and the activity of MMP2 and MMP9. Int. J. Clin. Exp. Pathol. *8*, 9990–10001.

Mancikova, V., Buj, R., Castelblanco, E., Inglada-Perez, L., Diez, A., de Cubas, A.A., Curras-Freixes, M., Maravall, F.X., Mauricio, D., Matias-Guiu, X., et al. (2014). DNA methylation profiling of well-differentiated thyroid cancer uncovers markers of recurrence free survival. Int. J. Cancer *135*, 598–610.

Mangat, C.S., Bharat, A., Gehrke, S.S., and Brown, E.D. (2014). Rank ordering plate data facilitates data visualization and normalization in high-throughput screening. J. Biomol. Screen *19*, 1314–1320.

Meyer, H., and Weihl, C.C. (2014). The VCP/p97 system at a glance: connecting cellular function to disease pathogenesis. J. Cell Sci. *127*, 3877–3883.

Montor, W.R., Salas, A., and Melo, F.H.M. (2018). Receptor tyrosine kinases and downstream pathways as druggable targets for cancer treatment: the current arsenal of inhibitors. Mol. Cancer *17*, 55.

Nagarajah, J., Le, M., Knauf, J.A., Ferrandino, G., Montero-Conde, C., Pillarsetty, N., Bolaender, A., Irwin, C., Krishnamoorthy, G.P., Saqcena, M., et al. (2016). Sustained ERK inhibition maximizes responses of BrafV600E thyroid cancers to radioiodine. J. Clin. Invest. *126*, 4119–4124.

Oh, J.M., Kalimuthu, S., Gangadaran, P., Baek, S.H., Zhu, L., Lee, H.W., Rajendran, R.L., Hong, C.M., Jeong, S.Y., Lee, S.W., et al. (2018). Reverting iodine avidity of radioactive-iodine refractory thyroid cancer with a new

tyrosine kinase inhibitor (K905-0266) excavated by high-throughput NIS (sodium iodide symporter) enhancer screening platform using dual reporter gene system. Oncotarget *9*, 7075–7087.

Passon, N., Puppin, C., Lavarone, E., Bregant, E., Franzoni, A., Hershman, J.M., Fenton, M.S., D'Agostino, M., Durante, C., Russo, D., et al. (2012). Cyclic AMP-response element modulator inhibits the promoter activity of the sodium iodide symporter gene in thyroid cancer cells. Thyroid *22*, 487–493.

Plantinga, T.S., Tesselaar, M.H., Morreau, H., Corssmit, E.P., Willemsen, B.K., Kusters, B., van Engen-van Grunsven, A.C., Smit, J.W., and Netea-Maier, R.T. (2016). Autophagy activity is associated with membranous sodium iodide symporter expression and clinical response to radioiodine therapy in non-medullary thyroid cancer. Autophagy *12*, 1195–1205.

Pugliese, M., Fortunati, N., Germano, A., Asioli, S., Marano, F., Palestini, N., Frairia, R., Boccuzzi, G., and Catalano, M.G. (2013). Histone deacetylase inhibition affects sodium iodide symporter expression and induces 131I cytotoxicity in anaplastic thyroid cancer cells. Thyroid *23*, 838–846.

Puppin, C., D'Aurizio, F., D'Elia, A.V., Cesaratto, L., Tell, G., Russo, D., Filetti, S., Ferretti, E., Tosi, E., Mattei, T., et al. (2005). Effects of histone acetylation on sodium iodide symporter promoter and expression of thyroid-specific transcription factors. Endocrinology *146*, 3967–3974.

Ravera, S., Reyna-Neyra, A., Ferrandino, G., Amzel, L.M., and Carrasco, N. (2017). The sodium/iodide symporter (NIS): molecular physiology and preclinical and clinical applications. Annu. Rev. Physiol. *79*, 261–289.

Riesco-Eizaguirre, G., Rodriguez, I., De la Vieja, A., Costamagna, E., Carrasco, N., Nistal, M., and Santisteban, P. (2009). The BRAFV600E oncogene induces transforming growth factor beta secretion leading to sodium iodide symporter repression and increased malignancy in thyroid cancer. Cancer Res. *69*, 8317–8325.

Riesco-Eizaguirre, G., Wert-Lamas, L., Perales-Paton, J., Sastre-Perona, A., Fernandez, L.P., and Santisteban, P. (2015). The miR-146b-3p/PAX8/NIS regulatory circuit modulates the differentiation phenotype and function of thyroid cells during carcinogenesis. Cancer Res. *75*, 4119–4130.

Schlumberger, M., Brose, M., Elisei, R., Leboulleux, S., Luster, M., Pitoia, F., and Pacini, F. (2014). Definition and management of radioactive iodine-refractory differentiated thyroid cancer. Lancet Diabetes Endocrinol. *2*, 356–358.

Segura-Cabrera, A., Tripathi, R., Zhang, X., Gui, L., Chou, T.F., and Komurov, K. (2017). A structure- and chemical genomics-based approach for repositioning of drugs against VCP/p97 ATPase. Sci. Rep. *7*, 44912.

Skrott, Z., Mistrik, M., Andersen, K.K., Friis, S., Majera, D., Gursky, J., Ozdian, T., Bartkova, J., Turi, Z., Moudry, P., et al. (2017). Alcohol-abuse drug disulfiram targets cancer via p97 segregase adaptor NPL4. Nature *552*, 194–199.

Smith, V.E., Read, M.L., Turnell, A.S., Watkins, R.J., Watkinson, J.C., lewy, g.d., Fong, J.C., James, S.R., Eggo, M.C., Boelaert, K., et al. (2009). A novel mechanism of sodium iodide symporter repression in differentiated thyroid cancer. J. Cell Sci. *122*, 3393–3402.

Smith, V.E., Sharma, N., Read, M.L., Ryan, G., Martin, A., Boelaert, K., Franklyn, J.A., and McCabe, C.J. (2013). Manipulation of PBF/PTTG1IP phosphorylation status; a new therapeutic strategy for improving radioiodine uptake in thyroid and other tumours. J. Clin. Endocrinol. Metab. *98*, 2876–2886.

Spitzweg, C., Harrington, K.J., Pinke, L.A., Vile, R.G., and Morris, J.C. (2001). Clinical review 132: the sodium iodide symporter and its potential role in cancer therapy. J. Clin. Endocrinol. Metab. *86*, 3327–3335.

Subramanian, A., Narayan, R., Corsello, S.M., Peck, D.D., Natoli, T.E., Lu, X., Gould, J., Davis, J.F., Tubelli, A.A., Asiedu, J.K., et al. (2017). A next generation connectivity Map: L1000 platform and the first 1,000,000 profiles. Cell *171*, 1437–1452 e1417.

Tazebay, U.H., Wapnir, I.L., Levy, O., Dohan, O., Zuckier, L.S., Zhao, Q.H., Deng, H.F., Amenta, P.S., Fineberg, S., Pestell, R.G., et al. (2000). The mammary gland iodide transporter is expressed during lactation and in breast cancer. Nat. Med. *6*, 871–878.

Tresse, E., Salomons, F.A., Vesa, J., Bott, L.C., Kimonis, V., Yao, T.P., Dantuma, N.P., and Taylor, J.P. (2010). VCP/p97 is essential for maturation of ubiquitin-containing autophagosomes and this function is impaired by mutations that cause IBMPFD. Autophagy *6*, 217–227.

Wachter, S., Damanakis, A.I., Elxnat, M., Roth, S., Wunderlich, A., Verburg, F.A., Fellinger, S.A., Bartsch, D.K., and Di Fazio, P. (2018). Epigenetic modifications in thyroid cancer cells restore NIS and radio-iodine uptake and promote cell death. J. Clin. Med. *7*, 61.

Yamaguchi, T., Hashizume, T., Matsuda, M., Sakashita, M., Fujii, T., Sekine, Y., Nakashima, M., and Uematsu, T. (1994). Pharmacokinetics of the H1-receptor antagonist ebastine and its active metabolite carebastine in healthy subjects. Arzneimittelforschung *44*, 59–64.

Zaballos, M.A., and Santisteban, P. (2017). Key signaling pathways in thyroid cancer. J. Endocrinol. *235*, R43–R61.

Zhang, X., Hu, P., Ding, S.Y., Sun, T., Liu, L., Han, S., DeLeo, A.B., Sadagopan, A., Guo, W., and Wang, X. (2019). Induction of autophagy-dependent apoptosis in cancer cells through activation of ER stress: an uncovered anticancer mechanism by anti-alcoholism drug disulfiram. Am. J. Cancer Res. *9*, 1266–1281.

Zhang, Z., Liu, D., Murugan, A.K., Liu, Z., and Xing, M. (2014). Histone deacetylation of NIS promoter underlies BRAF V600E-promoted NIS silencing in thyroid cancer. Endocr. Relat. Cancer *21*, 161–173.

## STAR★METHODS

### KEY RESOURCES TABLE

| REAGENT or RESOURCE | SOURCE | IDENTIFIER |
|---|---|---|
| Antibodies | | |
| Rabbit polyclonal anti-NIS | Proteintech | Cat# 24324-1-AP, RRID: AB_2879495 |
| Rabbit polyclonal anti-VCP | Proteintech | Cat# 10736-1-AP, RRID: AB_2214635 |
| Rabbit polyclonal anti-p62/SQSTM1 | Proteintech | Cat# 18420-1-AP, RRID: AB_10694431 |
| Rabbit polyclonal anti-LC3B | Cell Signalling Technology | Cat# 2775, RRID: AB_915950 |
| Mouse monoclonal anti-HA | BioLegend | Cat# 901501, RRID: AB_2565006 |
| Mouse monoclonal anti-β-actin | Sigma-Aldrich | Cat# A1978, RRID: AB_476692 |
| Bacterial and Virus Strains | | |
| Subcloning Efficiency™ DH5α Competent Cells | ThermoFisher | Cat# 18265017 |
| Biological Samples | | |
| Human thyroid tissue | Queen Elizabeth Hospital, Birmingham | N/A |
| Chemicals, Peptides, and Recombinant Proteins | | |
| Astemizole | Sigma-Aldrich | Cat# 2861 |
| Carebastine | Sigma-Aldrich | Cat# 24186 |
| CB-5083 | Cayman Chemical | Cat# 19311 |
| Chloroquine Diphosphate | Sigma-Aldrich | Cat# C6628 |
| Clotrimazole | Sigma-Aldrich | Cat# C6019 |
| DBeQ | Sigma-Aldrich | Cat# 506190 |
| Dimethyl Sulfoxide | Sigma-Aldrich | Cat# D2650 |
| Disulfiram | Sigma-Aldrich | Cat# 86720 |
| Dynasore | Sigma-Aldrich | Cat# D9673 |
| Ebastine | Sigma-Aldrich | Cat# E9531 |
| Eeyarestatin-1 | Cayman Chemicals | Cat# 10012609 |
| Fexofenadine HCL | Sigma-Aldrich | Cat# F9427 |
| Formoterol Hemifumarate | Stratech Scientific | Cat# B1359 |
| Imatinib Mesylate | Cambridge Bioscience | Cat# 13139 |
| Niflumic Acid | Alfa Aesar | Cat# J60489 |
| NMS-873 | SelleckChem | Cat# S7285 |
| Phenformin HCL | Stratech Scientific | Cat# B1373 |
| Phosphate Buffered Saline | ThermoFisher | Cat# 14190094 |
| Pravastatin Sodium Salt | Cambridge Bioscience | Cat# 10010343 |
| SAHA | Stratech Scientific | Cat# S1047 |
| Selumetinib | SelleckChem | Cat# S1008 |
| Sodium Iodide | Sigma-Aldrich | Cat# 383112 |
| Sodium Perchlorate | Sigma-Aldrich | Cat# 410241 |
| Sulconazole Nitrate | Sigma-Aldrich | Cat# 1623681 |
| Terfenadine | Sigma-Aldrich | Cat# T9652 |
| TRI Reagent | Sigma-Aldrich | Cat# 93289 |
| Vatalanib 2HCL | Stratech Scientific | Cat# A1778 |
| Vemurafenib | SelleckChem | Cat# S1267 |
| Zimelidine 2HCL | Sigma-Aldrich | Cat# Z101 |
| Critical Commercial Assays | | |
| Lipofectamine RNAiMAX | ThermoFisher Scientific | Cat# 13778075 |
| RNeasy Micro Kit | Qiagen | Cat# 74004 |
| Reverse Transcription System | Promega | Cat# A3500 |

(*Continued on next page*)

| Continued | | |
|---|---|---|
| REAGENT or RESOURCE | SOURCE | IDENTIFIER |
| EndoFree Plasmid Maxi Kit | Qiagen | Cat# 12362 |
| TaqMan® Gene Expression Master Mix | Applied Biosystems | Cat# 10525395 |
| Nano-Glo Live Cell Assay Solution | Promega | Cat# N2011 |
| NIS TaqMan® Gene Expression Assay | ThermoFisher Scientific | Cat# Hs00166567_m1 |
| PPIA TaqMan® Gene Expression Assay | ThermoFisher Scientific | Cat# Hs04194521_s1 |
| VCP TaqMan® Gene Expression Assay | ThermoFisher Scientific | Cat# HS00997642_m1 |
| Deposited data | | |
| The Connectivity Map (CMAP) | Subramanian et al., 2017 | https://clue.io/touchstone |
| Data source file | This paper | Mendeley Data: https://doi.org/10.17632/vf3xnyxyp9.1 |
| GSE27155 | Giordano et al., 2005 | https://www.ncbi.nlm.nih.gov/geo/geo2r/?acc=GSE27155 |
| GSE76039 | Landa et al., 2016 | https://www.ncbi.nlm.nih.gov/geo/geo2r/?acc=GSE76039 |
| TCGA GDAC Firehose standard data – Thyroid Carcinoma (THCA) | Broad Institute of MIT and Harvard | Firehose stddata__2016_01_28 run. https://doi.org/10.7908/C11G0KM9 |
| TCGA THCA | Grossman et al., 2016 | https://portal.gdc.cancer.gov/projects/TCGA-THCA |
| Thyroid carcinoma (TCGA, Firehose Legacy) | Cerami et al., 2012; Gao et al., 2013 | https://www.cbioportal.org/study/summary?id=thca_tcga |
| Experimental Models: Cell Lines | | |
| TPC-1 | Rebecca Schweppe's lab | N/A |
| TPC-1-YFP | This paper | N/A |
| TPC-1-NIS-YFP | This paper | N/A |
| 8505C | DSMZ | Cat# ACC-219, RRID:CVCL_1054 |
| 8505C-YFP | This paper | N/A |
| 8505C-NIS-YFP | This paper | N/A |
| MDA-MB-231 | ECACC | Cat# 92020424, RRID:CVCL_0062 |
| MCF7 | ECACC | Cat# 86012803, RRID:CVCL_0031 |
| MDA-MB-231-NIS | Fletcher et al., 2020 | N/A |
| HeLa | ECACC | Cat# 93021013, RRID:CVCL_0030 |
| Oligonucleotides | | |
| MISSION VCP siRNA | Sigma-Aldrich | Cat# NM_007126 |
| LgBiT (Forward)5'-GCCAAGCTTAC CATGGTCTTCACACTCGAA-3' | Sigma-Aldrich | Custom synthesis |
| LgBiT (Reverse)5'-GGCGGTACCAC TGTTGATGGTTA CTCG-3' | Sigma-Aldrich | Custom synthesis |
| VCP (Forward)5'-GCGGTACCGCTT CTGGAGCCGATTCA-3' | Sigma-Aldrich | Custom synthesis |
| VCP (Reverse)5'-GCCGCCTCTAG ATTAGCCATACAGGTCATC-3' | Sigma-Aldrich | Custom synthesis |
| NIS-SmBiT (Forward)5'-GCCGGTACC ACCATGGAGGCCGTGGAGACC-3' | Sigma-Aldrich | Custom synthesis |
| NIS-SmBiT (Reverse)5'-GCCTCTAGA TTACAGAATCTCCTCGAACAGCCGG TAGCCGGTCACGAGGTTTGTCTCC TGCTG-3' | Sigma-Aldrich | Custom synthesis |
| Recombinant DNA | | |
| pcDNA3.1-EYFP-H148Q/I152L/F46L | Alan Verkman's lab | N/A |
| pcDNA3.1-NIS | Smith et al., 2009 | N/A |

(Continued on next page)

| Continued | | |
|---|---|---|
| REAGENT or RESOURCE | SOURCE | IDENTIFIER |
| Halo-tagSmBiT (negative control) | Caroline Gorvin's lab | N/A |
| LgBiT-PRKAR2A (positive control) | Caroline Gorvin's lab | N/A |
| SmBiT-PRKACA (positive control) | Caroline Gorvin's lab | N/A |
| pcDNA3.1-LgBiT-VCP | This paper | N/A |
| pcDNA3.1-NIS-SmBiT | This paper | N/A |
| Software and Algorithms | | |
| GraphPad Prism Version 9 | Graphpad Software Inc | https://www.graphpad.com/scientificsoftware/prism/ |
| ImageJ | National Institutes of Health | https://imagej.nih.gov/ij/ |
| IBM SPSS Statistics Version 27 | IBM | https://www.ibm.com/uk-en/products/spss-statistics |
| BioRender | BioRender | https://biorender.com/ |
| EC50 Calculator | AAT Bioquest | https://www.aatbio.com/tools/ec50-calculator |
| GEO2R | National Center for Biotechnology Information | https://www.ncbi.nlm.nih.gov/geo/geo2r/ |
| DynaVenn | Chair for Clinical Bioinformatics at Saarland University | https://ccb-compute.cs.uni-saarland.de/dynavenn |
| Other | | |
| Prestwick Chemical Library | Birmingham Drug Discovery Facility | https://www.birmingham.ac.uk/facilities/bddf/compound-collections/index.aspx |
| Opera Phenix High-Content Screening System | Perkin Elmer | Cat# HH14000000 |
| Microlab STAR Liquid Handling System | Hamilton | https://www.hamiltoncompany.com/automated-liquid-handling/platforms/microlab-star |
| PHERAstar FS microplate reader | BMG Labtech | https://www.bmglabtech.com/pherastar-fsx/ |
| POLARstar Omega microplate reader | BMG Labtech | https://www.bmglabtech.com/polarstar-omega/ |
| Multi Crystal LB 2111 Gamma Counter | Berthold | https://www.berthold.com/en/ |
| 7500 Real-Time PCR system | ThermoFisher Scientific | Cat# 4351105 |

## RESOURCE AVAILABILITY

### Lead contact
Further information and requests for resources and reagents should be directed to the lead contact, Christopher McCabe (mccabcjz@bham.ac.uk).

### Materials availability
There are restrictions to the availability of cell lines (TPC-1, 8505C) and recombinant DNA (pcDNA3.1-EYFP-H148Q/I152L/F46L) due to material transfer agreement terms.

### Data and code availability
The datasets generated in this study have been deposited at Mendeley Data and are publicly available as of the date of publication. DOI is listed in the key resources table. This paper also analyzes existing, publicly available data. Identifiers for these datasets are listed in the key resources table. This paper does not report original code. Any additional information required to reanalyze the data reported in this paper is available from the lead contact upon request.

## EXPERIMENTAL MODEL AND SUBJECT DETAILS

### Cell lines

#### *Sources of cell lines*

TPC-1 cells were kindly provided by Dr Rebecca Schweppe (University of Colorado, Denver, USA). Other cell lines were obtained from DSMZ (Brunswick, Germany) and ECACC (Salisbury, UK). Human thyroid carcinoma cells TPC-1 and 8505C, and human breast cells MDA-MB-231 and MCF7 were cultured in RPMI-1640 (Life Technologies), and HeLa cells were cultured in DMEM Medium (Life Technologies), supplemented with penicillin (105 U/L), streptomycin (100 mg/L) and 10% FBS (Life Technologies). All cell lines were maintained at low passage, authenticated by short tandem repeat analysis (NorthGene), and tested for mycoplasma contamination (EZ-PCR; Geneflow).

#### *Generation of reporter cell lines*

Thyroidal reporter cell lines were made using EYFP-H148Q/I152L/F46L subcloned into pcDNA3.1 (Invitrogen, Hygromycin B-resistant; provided by Peter Haggie and Alan Verkman, UCSF) or full-length human NIS cDNA subcloned into pcDNA3.1 (Invitrogen, Geneticin-resistant; described in Smith et al., 2009). Stable TPC-1-YFP and 8505C-YFP cells were generated by transfection of parental cells with EYFP-H148Q/I152L/F46L/pcDNA3.1. Then Hygromycin B (Invitrogen, 150 μg/ml) was added to the media as a selection marker after 48 hours of transfection. Hygromycin B-resistant monoclonal colonies (i.e., 1 cell per well) were expanded following FACS single cell sorting (University of Birmingham Flow Cytometry Facility), and colonies with YFP fluorescence resolved by flow cytometry. Stable TPC-1-NIS-YFP and 8505C-NIS-YFP cells were generated by transfection of TPC-1-YFP or 8505C-YFP cells with NIS/pcDNA3.1. Geneticin (TPC-1-YFP cells, 300 μg/ml; 8505C-YFP cells, 500 μg/ml) was added to the media as a selection marker after 48 hours of transfection. Geneticin-resistant monoclonal colonies were expanded, and qPCR used to confirm NIS mRNA expression. Modified characteristics of stable cell lines were further verified by radioiodide ($^{125}$I) uptake assays and Opera Phenix$^{TM}$ live cell imaging (EvoTec, Germany). Generation of stable NIS-expressing Breast MDA-MB-231 cell lines by lentiviral transduction has been described in detail previously (Fletcher et al., 2020).

### Human primary thyroid tissue

The collection of normal human thyroid tissue was approved by the Local Research Ethics Committee (Birmingham Clinical Research Office, Birmingham, UK), and subjects gave informed written consent. No age/gender information was available as human subjects were anonymised and tissue collected as excess to surgery as part of our ethics agreement. To prepare primary thyrocyte cultures, thyroid tissue was homogenised and incubated overnight at RT with end-over-end-rotation in 0.2% collagenase (Worthington Biochemical Corporation) in HBSS (Sigma-Aldrich). Digested thyroid tissue was pelleted at 300 g for 10 minutes, which was washed 8x in HBSS by centrifugation at 300 g for 3 minutes. The pellet was re-suspended in Coon's F-12 modified liquid medium (with 2.5 g/l $NaHCO_3$, without L-glutamine) (Biochrom; Cambridge, UK) supplemented with 4% FBS, 1% L-glutamine (200 mM), penicillin (105 U/L), streptomycin (100 mg/L), thyroid-stimulating hormone (TSH) (0.3 U/L) (Sigma-Aldrich) and insulin (300 μg/L) (Sigma-Aldrich). Cells were then plated and maintained at 37°C and 5% $CO_2$ within a humidified environment. After at least 48 hr, FBS was omitted and cells cultured for approximately 6 days.

As a marker of thyroid function, the TSH-responsiveness of each batch of primary thyrocytes was verified. In brief, 3 days after cell plating, Coon's F-12 medium was replaced with FBS-free and TSH-free Coon's F-12 medium. After a further 3 days, medium was changed again to FBS-free Coon's F-12 medium containing TSH at 0 U/L, 0.3 U/L, 1 U/L or 10 U/L. On day 11, radioiodide uptake assays were performed to measure NIS function. Thyroid samples that responded to 0.3 U/L TSH were used for downstream analyses.

## METHOD DETAILS

### Drug screening strategy

High-throughput drug screening was performed via an automated biochemical screening platform using the Microlab STAR Liquid Handling Robotic System (Hamilton) integrated with a PHERAstar FS microplate reader (BMG Labtech; 510 nm-550 nm optic module). In brief, TPC-1 cells expressing NIS and halide-sensitive YFP were seeded into 96-well tissue culture plates at a density of 15,000 cells/well (black-walled, clear-bottom), using plates pre-loaded with test compounds. ~1200 FDA-approved compounds (Prestwick Chemical Library) were used for the primary high throughput screen at a single dose (10 μM; 1.25% DMSO). All plates contained positive (34 mM NaI) and negative (PBS) controls. The assay was performed using the PHERAstar FS microplate reader (BMG Labtech) at 37°C with orbital averaging, 20 flashes/well and focal height kept constant throughout. The initial YFP fluorescence intensity was recorded prior to injection of sodium iodide (NaI) at a final concentration of 4 mM. YFP fluorescence measurements were taken by the PHERAstar FS microplate reader every 3 minutes over 4 cycles and ΔYFP values calculated. A secondary multi-dose screen was performed using the same protocol to validate 73 drugs at 10 different doses (0.1 – 50 μM) in two YFP-expressing cell lines (i.e., TPC-1-NIS-YFP and TPC-1-YFP).

The Z' factor was used to validate the suitability of the YFP-iodide assay for high throughput drug screening. For this, the YFP fluorescence of TPC-1-NIS-YFP cells was quenched with different NaI concentrations (0-68 mM) and Z' factor values calculated using the following equation: $Z'\text{factor} = 1 - \left[\frac{3(SD_P + SD_n)}{(Mean_p - Mean_n)}\right]$

[SD = standard deviation; mean = mean YFP fluorescence; p = positive control (e.g., 34 or 68 mM NaI); n = negative control (e.g., 8 mM NaI or PBS). Z'factor > 0.5 indicated that the assay was suitable for a full-scale high throughput drug screen].

We used ΔYFP, area under the curve (AUC), EC50 and radioiodide ($^{125}I$) uptake values to rank the efficacy of drugs to increase intracellular iodide levels. AUC values were determined ($log_{10}$ scale; GraphPad Prism) for positive peaks only. AUC values for drug-treated TPC-1-YFP cells were subtracted from corresponding values in TPC-1-NIS-YFP cells to determine ΔAUC values. EC50 values were calculated using EC50 calculator [AAT Bioquest, Inc. (2020, April 08)].

## Inhibitors/ drugs used

Drugs used to treat cells in radioiodide uptake assays are listed in the key resources table. Chloroquine diphosphate was resuspended in PBS without calcium/magnesium (Thermo Fisher); Phenformin HCL in 100% ethanol and all other drugs in dimethyl sulfoxide (DMSO; Sigma-Aldrich), before being diluted in RPMI-1640 medium (Life Technologies) and added to cells at 1:100 dilution. Drugs reported to enhance NIS function and used as positive controls were DBeQ (Sigma-Aldrich), Dynasore (Sigma-Aldrich), Eeyarestatin-1 (Cayman Chemicals), NMS-873 (SelleckChem), Selumetinib (SelleckChem) and Vemurafenib (SelleckChem).

## Cell viability assay

Cells seeded in 96 well plates were incubated with alamarBlue (0.02%; resazurin) solution for 1 hour and absorbance measured using the POLARstar Omega plate reader (BMG Labtech; 572-583 nm optic module) according to manufacturer's protocols.

## Determination of ΔYFP values

ΔYFP values signify changes in intracellular iodide (i.e., positive ΔYFP values denote increased intracellular iodide) and were derived relative to standard deviation (SD) of YFP fluorescence of DMSO-treated TPC-1-NIS-YFP cells (n = 120 wells). Candidate drugs were considered top hits in the primary high throughput screen at ΔYFP values > 1.5-2. Raw YFP fluorescence measurements were normalised using an interquartile mean well-based method ($IQM_w$) (Mangat et al., 2014), according to the following equations:

$$\%YFP = \left[\frac{YFP_0 - YFP_4}{YFP_0}\right] * 100 \qquad \text{(Equation 1)}$$

(%YFP = Percentage change in YFP fluorescence; $YFP_0$ = Raw YFP fluorescence value at time 0 prior to NaI injection; $YFP_4$= Raw YFP fluorescence value after 4 cycles).

$$S_{IQM} = \frac{\%YFP}{\mu_{IQ}} \qquad \text{(Equation 2)}$$

[$S_{IQM}$ = Sample value after IQM normalization on a per plate basis; $\mu_{IQ}$ = TRIMMEAN (values for all wells, 0.5), i.e., interquartile mean of the plate].

$$S_{IQMW} = \frac{S_{IQM}}{\mu_{IQW}} \qquad \text{(Equation 3)}$$

[$S_{IQMW}$ = Sample value after IQM normalization for each well position; $\mu_{IQW}$ = TRIMMEAN (values for each well position, 0.5), i.e., mean (μ) of the interquartile values (IQ) of each well position (w) across the entire screen].

$$\Delta YFP = \frac{S_{IQMW}(DRUG_x) - S_{IQMW}(DMSO_x)}{SD_{SIQMW}(DMSO_n)} \qquad \text{(Equation 4)}$$

[ΔYFP = Fold-change in YFP fluorescence for drug treatment wells relative to SD of YFP fluorescence in DMSO control wells; $S_{IQMW}$ ($DRUG_x$) = Sample value after IQM normalization for each drug treatment well; $S_{IQMW}$ ($DMSO_x$) = Sample value after IQM normalization for plate matched DMSO control wells; $SD_{SIQMW}$ (DMSOn) = SD of sample values after IQM normalization for DMSO control wells across the entire screen, n = 120].

## Radioiodide uptake assay

Cells were seeded in 24-well plates with at least three biological replicates per condition, and then treated with drugs and/or transfected with siRNA. Cells were then incubated for 1 hour with 0.05 μCi iodine-125 ($^{125}I$) (Hartmann Analytic; Folkestone, UK) at a final concentration of $10^{-7}$ moles/l NaI. Following incubation, cells were washed twice in HBSS (Sigma-Aldrich) to remove unincorporated $^{125}I$ and lysed in 100 μl 2% SDS. Radioiodide uptake was determined using the Berthold Gamma Counter (Berthold) to measure gamma radiation (counts per minute). Protein concentrations were determined using the Pierce™ BCA colorimetric assay (Thermo-Fisher Scientific). $^{125}I$ uptake relative to protein concentration was calculated in picomoles of $^{125}I$/ μg protein, according to the following equation:

$$\text{Picomoles of 125I/ } \mu g \text{ protein} = \frac{5000 * \left(\frac{Counts}{Total\ Protein}\right)}{12000}$$

In control experiments, cells were treated with 100 μM sodium perchlorate for 1 hour at 37°C and 5% $CO_2$ prior to the assay to inhibit NIS-mediated radioiodide uptake.

### Immunoblots

For western blotting, primary antibodies were used against LC3B (1:1000; Cell Signalling Technology, cat# 2775), NIS (1:1000; Proteintech, cat# 24324-1-AP), p62/SQSTM1 (1:4000; Proteintech, cat# 18420-1-AP), VCP (1:1000; Proteintech, cat# 10736-1-AP), HA (1: 1000; BioLegend, cat# 901501), and β-actin (1:2000; Sigma-Aldrich, cat# A1978). An HRP-conjugated secondary antibody (Agilent Technologies) against either mouse or rabbit IgG was used at 1:10000 dilution.

Cells were washed with phosphate buffered saline (PBS) and then lysed in high salt buffer (50 mM Tris, pH 7.4, 400 mM NaCl, 1% NP-40 1% v/v Igepal CA-630) supplemented with protease and phosphatase inhibitors (Sigma-Aldrich). Protein concentrations were determined using the Pierce™ BCA colorimetric assay (ThermoFisher Scientific). Equal amounts of protein were resolved on SDS-PAGE and subsequently transferred onto polyvinylidene difluoride (PVDF) transfer membrane (ThermoFisher Scientific).

The membranes were blocked in 5% w/v skimmed milk powder (Marvel) in Tris-buffered saline containing tween (TBS-T, 20 mM Tris pH 7.6, 137 mM NaCl, Sigma-Aldrich), and then incubated with primary antibodies in 5% skimmed milk in TBS-T overnight at 4°C with gently shaking. After washing, membranes were incubated for 1 hour with horseradish peroxidase (HRP)-conjugated polyclonal goat anti-rabbit or rabbit anti-mouse secondary antibodies (Agilent Technologies) in 5% skimmed milk in TBS-T. The membrane was then washed three times in TBS-T prior to incubation with the ECL plus Western blotting substrate (ThermoFisher Scientific). Membranes were exposed to x-ray film (Scientific Laboratory Supplies) for antigen detection. Protein expression was quantified by scanning densitometry using ImageJ (National Institutes of Health).

### siRNA transfections

A pool of three siRNAs (MISSION; Sigma-Aldrich) targeted to VCP were used which had been designed using an algorithm from Rosetta Inpharmatics LLC (Washington, USA) and targeted the VCP gene at nucleotides 768 (Hs01_00118726), 1908 (Hs02_00343009) and 2548 (Hs01_00118728). In brief, MISSION VCP siRNA or a negative control scrambled siRNA (ThermoFisher Scientific) was diluted in Opti-MEM® I reduced serum medium (Life Technologies) at a final concentration of 100 nM. Lipofectamine RNAiMAX (ThermoFisher Scientific) at RT was briefly vortexed and added to the diluted siRNA at 6 μl/ml Opti-MEM® I reduced serum medium prior to vortexing and incubating at RT for 25 minutes to facilitate siRNA-lipid complex formation. Medium from cells was replaced with the transfection mix at 1 ml/well in 6-well plates and 250 μl/well in 24-well plates and gently mixed. 6 hours later, transfection mix was replaced with complete medium. Downstream analyses were performed at least 48 hours post-transfection.

### qPCR

Cells were lysed directly in 12-well plates using TRI reagent (Sigma-Aldrich) and RNA was isolated using the RNeasy Micro Kit (Qiagen) following manufacturers protocol. cDNA was synthesized from RNA using the Reverse Transcription System (Promega) following manufacturers protocols. qPCR was performed using TaqMan® Gene Expression Master Mix (Applied Biosystems) on an Applied Biosystems 7500 Real-Time PCR system (Thermo Fisher Scientific). Real-time qPCR expression assays used are listed in the key resources table. RNA expression was determined using the $2^{-\Delta\Delta Ct}$ method.

### Plasmid transfections

To construct plasmids for NanoBiT assays VCP cDNA was cloned into pcDNA3.1 with an added N-terminal LgBiT tag, whereas a SmBiT tag was added to the C-Terminal of NIS cDNA. Control plasmids included Halo-tagSmBiT (negative control vector) and LgBiT-PRKAR2A/SmBiT-PRKACA (positive control vectors), kindly provided by Dr Caroline Gorvin (University of Birmingham). Plasmid DNA was transfected into cells using TransIT®-LT1 (Mirus Bio) at a 3:1 ratio (lipid: DNA) following the manufacturer's protocol.

### NanoBiT cell-based assays

HeLa cells were seeded in 6-well plates at a density of $3.5 \times 10^5$ cells per well and transfected with 1 μg plasmid DNA. 24 hours post-transfection, cells were detached using trypsin, resuspended in phenol-red-free DMEM (Life Technologies), re-plated into white 96-well plates and incubated for five hours at 37°C to ensure sufficient attachment. Cells were then treated with disulfiram (Sigma-Aldrich) or CB-5083 (Cayman Chemical) for 24 hours. 25 μl Nano-Glo live cell assay solution (Promega) was added to each well and luminescence measured at 120 second intervals for 40 minutes using the PHERAstar FS microplate reader (BMG Labtech) preheated to 37°C.

### TCGA and GEO datasets

Normalized gene expression data and clinical information for papillary thyroid cancer (PTC) were downloaded from TCGA via cBioPortal (cbioportal.org/), FireBrowse (firebrowse.org) and NCI Genomic Data Commons (GDC; portal.gdc.cancer.gov/) (Cerami et al., 2012; Gao et al., 2013; Grossman et al., 2016). Gene expression values were transformed as $X=\log_2(X+1)$ where X represents the normalized fragments per kilobase transcript per million mapped reads (FPKM) values. In total, RNA-seq data for 59 normal thyroid and 501 PTC TCGA samples were analyzed (Broad GDAC Firehose, https://doi.org/10.7908/C11G0KM9).

Differential gene expression analysis was also performed using the GEO2R interactive web tool in GEO (Barrett et al., 2013) to investigate proteostasis genes in thyroid cancer. Thyroid cancer GEO datasets used were GSE27155 (Giordano et al., 2005) and GSE76039 (Landa et al., 2016). Significance of gene overlap between datasets was determined using the DynaVenn algorithm (see key resources table).

### Connectivity map analysis

The Connectivity Map (CMAP) resource (https://clue.io/touchstone) was used to probe the transcriptional relationship between top drug hits from the primary high-throughput screen (47 available in CMAP) and loss of function of a panel of 45 proteostasis genes (Lamb et al., 2006; Subramanian et al., 2017). Hierarchical cluster analysis was used to identify patterns of drugs associated with genes. This study used the current CMAP (L1000) dataset which relies on perturbational data generated with the L1000 assay. The previous Affymetrix-based CMAP dataset is referred to as (build 02).

## QUANTIFICATION AND STATISTICAL ANALYSIS

Details regarding statistical tests are reported in the legends to Figures 1, 2, 3, 4, and 5, Table 1 and the supplemental information (Figures S1–S7, and Tables S2, S5, S6, and S7). Statistical analyses were performed using IBM SPSS Statistics (Version 27), GraphPad Prism (Version 9) and Microsoft Excel. All results were obtained from triplicate biological experiments unless otherwise indicated. For comparison between two groups, data were subjected to the Student's *t*-test, and for multiple comparisons one-way ANOVA was used with either Dunnett's or Tukey's post-hoc test. $p < 0.05$ was considered significant. All p-values reported from statistical tests were two-sided.

Kolmogorov-Smirnov, Kruskal-Wallis, and Spearman's correlation tests were performed on non-parametric TCGA data. P-values were adjusted using the Benjamini-Hochberg FDR correction procedure to correct for multiple comparisons. Dunn's multiple comparison post-hoc testing was used after Kruskal-Wallis tests to determine significance between datasets in groups of 3 or more. Fisher's exact test was used to determine the significance of nonrandom associations between two categorical variables.

Cell Chemical Biology, Volume *29*

# Supplemental information

# Targeting non-canonical pathways as a strategy to modulate the sodium iodide symporter

**Martin L. Read, Katie Brookes, Caitlin E.M. Thornton, Alice Fletcher, Hannah R. Nieto, Mohammed Alshahrani, Rashida Khan, Patricia Borges de Souza, Ling Zha, Jamie R.M. Webster, Luke J. Alderwick, Moray J. Campbell, Kristien Boelaert, Vicki E. Smith, and Christopher J. McCabe**

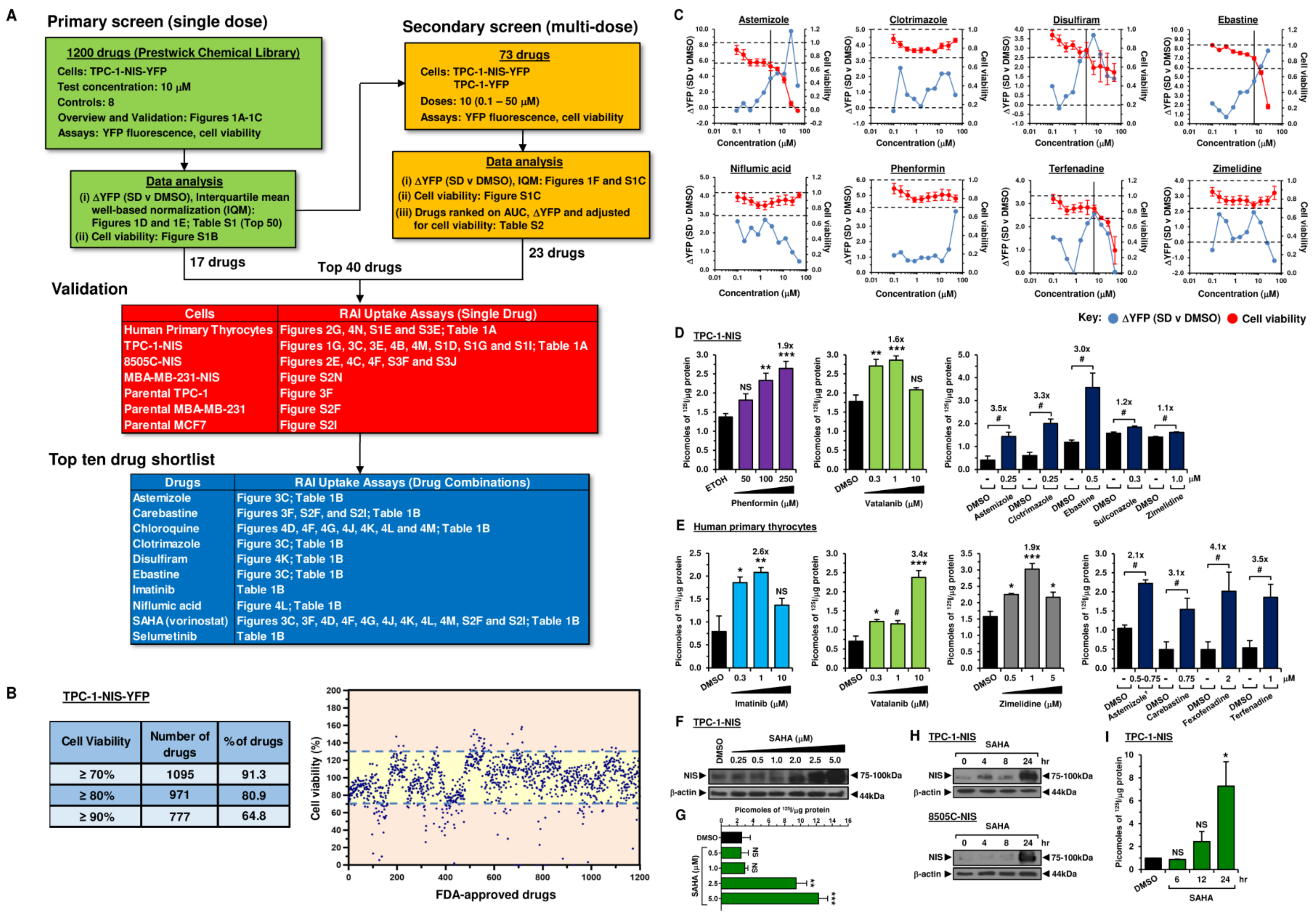
A
Primary screen (single dose)
1200 drugs (Prestwick Chemical Library)
Cells: TPC-1-NIS-YFP
Test concentration: 10 μM
Controls: 8
Overview and Validation: Figures 1A-1C
Assays: YFP fluorescence, cell viability
Data analysis
(i) ΔYFP (SD v DMSO), Interquartile mean well-based normalization (IQM): Figures 1D and 1E; Table S1 (Top 50)
(ii) Cell viability: Figure S1B
Secondary screen (multi-dose)
73 drugs
Cells: TPC-1-NIS-YFP
TPC-1-YFP
Doses: 10 (0.1 – 50 μM)
Assays: YFP fluorescence, cell viability
Data analysis
(i) ΔYFP (SD v DMSO), IQM: Figures 1F and S1C
(ii) Cell viability: Figure S1C
(iii) Drugs ranked on AUC, ΔYFP and adjusted for cell viability: Table S2
17 drugs
Top 40 drugs
23 drugs
Validation
Cells | RAI Uptake Assays (Single Drug)
Human Primary Thyrocytes | Figures 2G, 4N, S1E and S3E; Table 1A
TPC-1-NIS | Figures 1G, 3C, 3E, 4B, 4M, S1D, S1G and S1I; Table 1A
8505C-NIS | Figures 2E, 4C, 4F, S3F and S3J
MBA-MB-231-NIS | Figure S2N
Parental TPC-1 | Figure 3F
Parental MBA-MB-231 | Figure S2F
Parental MCF7 | Figure S2I
Top ten drug shortlist
Drugs | RAI Uptake Assays (Drug Combinations)
Astemizole | Figure 3C; Table 1B
Carebastine | Figures 3F, S2F, and S2I; Table 1B
Chloroquine | Figures 4D, 4F, 4G, 4J, 4K, 4L and 4M; Table 1B
Clotrimazole | Figure 3C; Table 1B
Disulfiram | Figure 4K; Table 1B
Ebastine | Figure 3C; Table 1B
Imatinib | Table 1B
Niflumic acid | Figure 4L; Table 1B
SAHA (vorinostat) | Figures 3C, 3F, 4D, 4F, 4G, 4J, 4K, 4L, 4M, S2F and S2I; Table 1B
Selumetinib | Table 1B
B
TPC-1-NIS-YFP
Cell Viability | Number of drugs | % of drugs
≥ 70% | 1095 | 91.3
≥ 80% | 971 | 80.9
≥ 90% | 777 | 64.8
Cell viability (%)
FDA-approved drugs
C
Astemizole
Clotrimazole
Disulfiram
Ebastine
Niflumic acid
Phenformin
Terfenadine
Zimelidine
ΔYFP (SD v DMSO)
Cell viability
Concentration (μM)
Key: ΔYFP (SD v DMSO) Cell viability
D TPC-1-NIS
Picomoles of 125I/μg protein
ETOH 50 100 250
Phenformin (μM)
NS ** 1.9x ***
DMSO 0.3 1 10
Vatalanib (μM)
** 1.6x *** NS
3.5x # 3.3x # 3.0x # 1.2x # 1.1x #
DMSO Astemizole DMSO Clotrimazole DMSO Ebastine DMSO Sulconazole DMSO Zimelidine
- 0.25 - 0.25 - 0.5 - 0.3 - 1.0 μM
E Human primary thyrocytes
DMSO 0.3 1 10
Imatinib (μM)
* 2.6x ** NS
Vatalanib (μM)
* # 3.4x ***
DMSO 0.5 1 5
Zimelidine (μM)
* 1.9x *** *
DMSO Astemizole DMSO Carebastine DMSO Fexofenadine DMSO Terfenadine
- 0.5-0.75 - 0.75 - 2 - 1 μM
2.1x # 3.1x # 4.1x # 3.5x #
F TPC-1-NIS
SAHA (μM)
DMSO 0.25 0.5 1.0 2.0 2.5 5.0
NIS 75-100kDa
β-actin 44kDa
G
Picomoles of 125I/μg protein
DMSO 0.5 1.0 2.5 5.0
SAHA (μM)
NS NS ** ***
H TPC-1-NIS
SAHA
0 4 8 24 hr
NIS 75-100kDa
β-actin 44kDa
8505C-NIS
SAHA
0 4 8 24 hr
NIS 75-100kDa
β-actin 44kDa
I TPC-1-NIS
Picomoles of 125I/μg protein
DMSO 6 12 24 hr
SAHA
NS NS *

**Figure S1 (related to Fig. 1, 2G, 3C, 3D). Identification of FDA-Approved Drugs that Enhance NIS Function**

(**A**) High throughput screen (HTS) based on a newly adapted YFP biosensor assay was used in the primary screen (green boxes) to determine the efficacy of 1200 drugs (Prestwick Chemical Library, single dose) to increase intracellular iodide in TPC1-NIS-YFP cells. A secondary multi-dose screen (orange boxes) was undertaken to validate 73 of these drugs at 10 different doses in two YFP-expressing cell lines (i.e., TPC-1-NIS-YFP and TPC1-YFP). From this, a total of 40 drugs were validated in radioiodide uptake assays (red box) in multiple cell types as listed. Finally, a shortlist of 10 drugs was generated (blue box) and different drug combinations evaluated in radioiodide uptake assays. (**B**) TPC-1-NIS-YFP cell viability following treatment with a 10 μM dose of 1200 drugs (Prestwick Chemical Library) for 24 hr. Each circle represents a mean value from 2 biological replicates. Table (left) summarizes number of drugs associated with TPC-1-NIS-YFP cellular viability equal or greater than 70, 80 or 90%. Cellular viability determined using the alamarBlue (resazurin) assay. (**C**) YFP-based biosensing and cell viability of TPC-1-NIS-YFP cells treated for 24 hr at 10 different drug doses (0.1 – 50 μM). 8 out of 73 representative drug profiles are shown. Blue circles represent mean ΔYFP values from 2 biological replicates. Cell viability (red circles) was determined using the alamarBlue (resazurin) assay (Mean ± S.E.M., n = 4). Pharmacologic parameters (e.g., AUC) were determined up to maximum drug dose associated with cell viability > 70% (indicated by vertical black line). (**D, E**) Radioiodide uptake of TPC-1-NIS cells (D) and primary human thyrocytes (E) treated with the indicated drug for 24 hr. Data for astemizole[†] is compiled from human primary thyrocytes treated with two different doses (0.5 and 0.75 μM). (**F**) Western blot analysis of NIS expression in TPC-1-NIS cells treated with SAHA at indicated doses for 24 hr. (**G**) Radioiodide uptake of TPC-1-NIS cells treated with SAHA at indicated doses for 24 hr.(**H**) Western blot analyses of NIS protein levels in TPC-1-NIS (upper) and 8505C-NIS cells (lower) at indicated times post-treatment (hr) with 2.5 μM SAHA. (**I**) Same as (D) but radioiodide uptake in TPC-1-NIS cells at indicated times post-treatment (hr) with 2.5 μM SAHA. Data presented as mean ± S.E.M., n = 3, one-way ANOVA followed by Dunnett's post hoc test (NS, not significant; $*p < 0.05$; $**p < 0.01$; $***p < 0.001$) or unpaired two-tailed *t*-test ($^{\#}p < 0.05$).

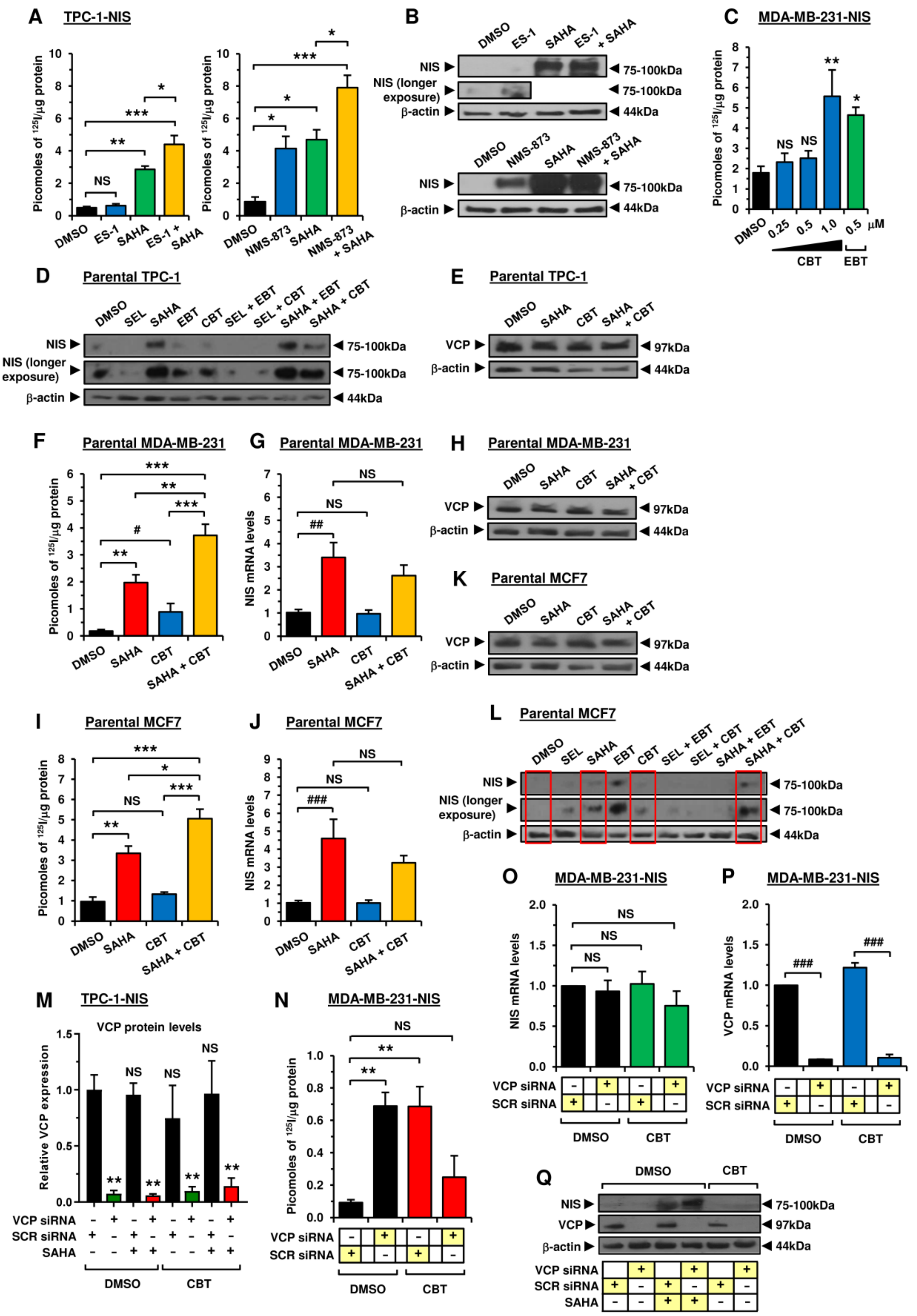
A
TPC-1-NIS
Picomoles of 125I/µg protein
DMSO
ES-1
SAHA
ES-1 + SAHA
NMS-873
NMS-873 + SAHA
NS
B
NIS
NIS (longer exposure)
β-actin
75-100kDa
44kDa
C
MDA-MB-231-NIS
µM
CBT
EBT
D
Parental TPC-1
SEL
EBT
CBT
SEL + EBT
SEL + CBT
SAHA + EBT
SAHA + CBT
E
VCP
97kDa
F
Parental MDA-MB-231
G
NIS mRNA levels
H
K
Parental MCF7
I
J
L
M
VCP protein levels
Relative VCP expression
VCP siRNA
SCR siRNA
N
O
P
VCP mRNA levels
Q

**Figure S2 (related to Fig. 3). Combined VCP Inhibitor and SAHA Treatment Augments Radioiodide Uptake in Thyroid and Breast Cancer Cells**

(**A, B**) Radioiodide uptake (A) and relative NIS expression (B) in TPC-1-NIS cells treated with 2.5 μM eeyarestatin-1 (ES-1) or 5.0 μM NMS-873 either alone or in combination with 2.5 μM SAHA. SAHA was added 12 hr prior to ES-1 or NMS-873. (**C**) Radioiodide uptake of MDA-MB-231-NIS cells treated with carebastine (CBT) at indicated doses for 24 hr. Controls: DMSO and 0.5 μM ebastine (EBT). (**D**) Relative NIS protein expression in parental TPC-1 cells treated with 0.2 μM selumetinib (SEL), 2.5 μM SAHA, 0.5 μM EBT and 1.0 μM CBT, either alone or in combination as indicated. SEL treatment included for comparison. (**E**) Relative VCP expression in parental TPC-1 cells treated with 2.5 μM SAHA and 1.0 μM CBT either alone or in combination. (**F, G**) Radioiodide uptake (F) and relative NIS mRNA levels (G) in parental MDA-MB-231 cells treated with 2.5 μM SAHA and 1.0 μM CBT either alone or in combination. (**H**) Same as (E) but with parental MDA-MB-231 cells. (**I-K**) Same as (F-H) but with parental MCF7 cells. (**L**) Same as (D) but with parental MCF7 cells. Red boxes highlight induction of NIS protein by SAHA and SAHA+CBT compared to DMSO or CBT treatment alone. (**M**) Relative VCP protein levels in TPC-1-NIS cells following VCP-siRNA depletion and treatment with 1.0 μM CBT and/or 2.5 μM SAHA. (**N, O**) Radioiodide uptake (N) and relative NIS mRNA levels (O) in MDA-MB-231-NIS cells following VCP-siRNA depletion and treatment with 1.0 μM CBT. (**P, Q**) Relative VCP mRNA (P) and protein levels (Q), as well as NIS expression (Q), in MDA-MB-231-NIS cells following VCP-siRNA depletion and treatment with 1.0 μM CBT and/or 2.5 μM SAHA. Data presented as mean ± S.E.M., n = 3-6, one-way ANOVA followed by Tukey's (A, F, I) or Dunnett's (C, M, N, O) post hoc test (NS, not significant; $^{*}p < 0.05$; $^{**}p < 0.01$; $^{***}p < 0.001$) or unpaired two-tailed *t*-test ($^{\#}p < 0.05$; $^{\#\#}p < 0.01$; $^{\#\#\#}p < 0.001$).

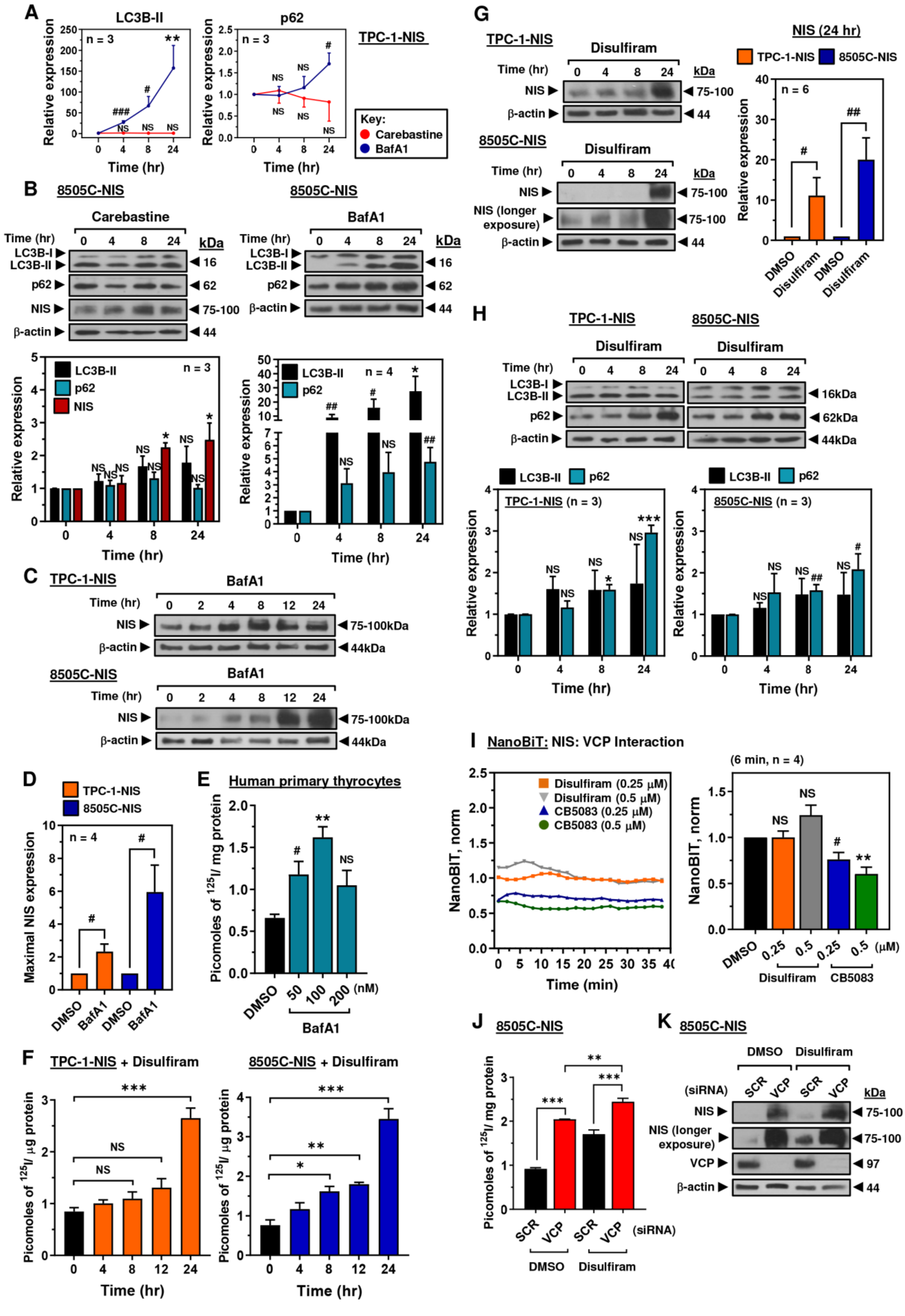

A
LC3B-II
p62
TPC-1-NIS
n = 3
Relative expression
Time (hr)
Key:
Carebastine
BafA1
B
8505C-NIS
Carebastine
BafA1
Time (hr)
LC3B-I
LC3B-II
p62
NIS
β-actin
kDa
16
62
75-100
44
LC3B-II
p62
NIS
n = 3
n = 4
C
TPC-1-NIS
8505C-NIS
BafA1
75-100kDa
44kDa
D
TPC-1-NIS
8505C-NIS
n = 4
Maximal NIS expression
DMSO
BafA1
E
Human primary thyrocytes
Picomoles of 125I/ mg protein
(nM)
F
TPC-1-NIS + Disulfiram
8505C-NIS + Disulfiram
Picomoles of 125I/ µg protein
G
TPC-1-NIS
8505C-NIS
Disulfiram
NIS (longer exposure)
NIS (24 hr)
n = 6
DMSO
Disulfiram
H
TPC-1-NIS (n = 3)
8505C-NIS (n = 3)
16kDa
62kDa
44kDa
I
NanoBiT: NIS: VCP Interaction
Disulfiram (0.25 µM)
Disulfiram (0.5 µM)
CB5083 (0.25 µM)
CB5083 (0.5 µM)
NanoBIT, norm
Time (min)
(6 min, n = 4)
(µM)
CB5083
J
8505C-NIS
SCR
VCP
(siRNA)
DMSO
Disulfiram
K
8505C-NIS
VCP
97

**Figure S3 (related to Fig. 3L, 4A-E). Autophagy Inhibitor BafA1 Augments NIS Expression and Radioiodide Uptake in Thyroid Cells**

(**A**) Scanning densitometry performed relative to β-actin on Western blot analysis of LC3B-II (left) and p62 protein levels (right) in TPC-1-NIS cells treated with 1.0 μM carebastine (CBT, red spots) or 100 nM bafilomycin A1 (bafA1, blue spots) for the indicated timecourse (hr). See also Figure 3L. (**B**) Western blot analysis of LC3B-I, LC3B-II and p62 protein levels in 8505C-NIS cells treated with 1.0 μM CBT (left) or 100 nM bafA1 (right) for the indicated timecourse (hr). Scanning densitometry (below) performed relative to β-actin. Relative NIS expression levels are also shown for CBT treated 8505-NIS cells. (**C-D**) Western blot analysis of NIS protein levels in TPC-1-NIS and 8505C-NIS cells treated with 100 nM bafA1 for the indicated timecourse (hr). Scanning densitometry performed relative to β-actin (D). (**E**) Radioiodide uptake of primary human thyrocytes treated with bafA1 at the indicated dose for 24 hr. (**F**) Radioiodide uptake of TPC-1-NIS and 8505C-NIS cells treated with 0.5 μM disulfiram for the indicated timecourse. (**G**) Relative NIS protein levels in TPC-1-NIS and 8505C-NIS cells treated with 0.5 μM disulfiram for the indicated timecourse (hr). Scanning densitometry (right) performed relative to β-actin (n = 6). (**H**) Western blot analysis of LC3B-I, LC3B-II and p62 protein levels in TPC-1-NIS and 8505C-NIS cells treated with 0.5 μM disulfiram for the indicated timecourse (hr). Scanning densitometry (below) performed relative to β-actin (n = 3). (**I**) Evaluation of VCP binding to NIS using NanoBiT in HeLa cells treated with disulfiram or VCP inhibitor CB5083 for 24 hr at the indicated dose (left, n = 4). Normalised NanoBiT assay results shown at 6 min after addition of Nano-Glo live cell assay solution (right). (**J**) Radioiodide uptake of 8505C-NIS cells following VCP-siRNA depletion and treatment with 0.5 μM disulfiram. (**K**) Relative NIS and VCP protein levels in 8505C-NIS cells following VCP-siRNA depletion and treatment with 0.5 μM disulfiram. Data presented as mean ± S.E.M., one-way ANOVA followed by Dunnett's post hoc test (NS, not significant; *p < 0.05; **p < 0.01; ***p < 0.001) or unpaired two-tailed *t*-test ($^{\#}$p < 0.05; $^{\#\#}$p < 0.01; $^{\#\#\#}$p < 0.01).

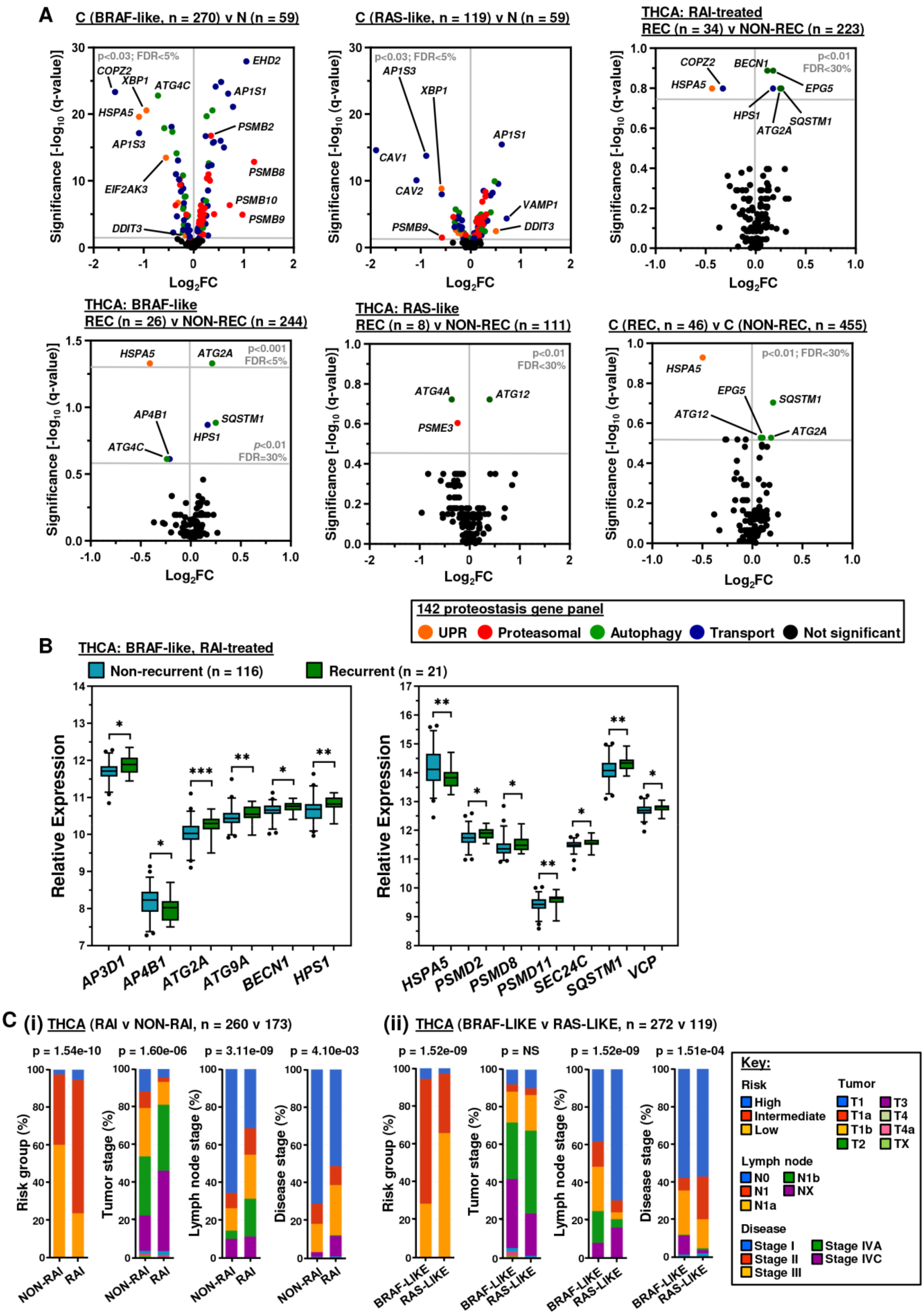

A
C (BRAF-like, n = 270) v N (n = 59)
C (RAS-like, n = 119) v N (n = 59)
THCA: RAI-treated
REC (n = 34) v NON-REC (n = 223)
p<0.03; FDR<5%
p<0.01
FDR<30%
Significance [-log10 (q-value)]
Log2FC
COPZ2
XBP1
ATG4C
HSPA5
AP1S3
EIF2AK3
DDIT3
EHD2
AP1S1
PSMB2
PSMB8
PSMB10
PSMB9
CAV1
CAV2
VAMP1
BECN1
EPG5
HPS1
SQSTM1
ATG2A
THCA: BRAF-like
REC (n = 26) v NON-REC (n = 244)
THCA: RAS-like
REC (n = 8) v NON-REC (n = 111)
C (REC, n = 46) v C (NON-REC, n = 455)
p<0.001
FDR<5%
p<0.01
FDR=30%
AP4B1
ATG4A
ATG12
PSME3
p<0.01; FDR<30%
142 proteostasis gene panel
UPR
Proteasomal
Autophagy
Transport
Not significant
B
THCA: BRAF-like, RAI-treated
Non-recurrent (n = 116)
Recurrent (n = 21)
Relative Expression
AP3D1
AP4B1
ATG2A
ATG9A
BECN1
HPS1
HSPA5
PSMD2
PSMD8
PSMD11
SEC24C
SQSTM1
VCP
C (i) THCA (RAI v NON-RAI, n = 260 v 173)
(ii) THCA (BRAF-LIKE v RAS-LIKE, n = 272 v 119)
p = 1.54e-10
p = 1.60e-06
p = 3.11e-09
p = 4.10e-03
p = 1.52e-09
p = NS
p = 1.52e-09
p = 1.51e-04
Risk group (%)
Tumor stage (%)
Lymph node stage (%)
Disease stage (%)
NON-RAI
RAI
BRAF-LIKE
RAS-LIKE
Key:
Risk
High
Intermediate
Low
Tumor
T1
T1a
T1b
T2
T3
T4
T4a
TX
Lymph node
N0
N1
N1a
N1b
NX
Disease
Stage I
Stage II
Stage III
Stage IVA
Stage IVC

**Figure S4 (related to Fig. 5A-C). Identification of Proteostasis Modulators Associated with Cancer Genetic Signature, Recurrence and Radioiodide Therapy**

(**A**) (upper) Volcano plots comparing $log_2$FC with q-value (-log base 10) for 142 core proteostasis genes in the BRAF-like (left) or RAS-like (middle) THCA cohort (C) versus normal (N). (upper right) Volcano plot illustrating $log_2$FC with q-value (-log base 10) for 142 core proteostasis genes in the RAI-treated THCA cohort [recurrent (REC) versus non-recurrent (NON-REC)]. (lower) Volcano plots comparing $log_2$FC with q-value (-log base 10) for 142 core proteostasis genes in the BRAF-like (left), RAS-like (middle) or entire (right) THCA cohort [REC versus NON-REC]. Gene categories indicated by coloured spots. (**B**) Box and whisker plots showing expression ($log_2$) of 13 core proteostasis genes in the BRAF-like, RAI-treated THCA cohort [recurrent (REC) versus non-recurrent (NON-REC)]; P-values determined by Mann-Whitney test and adjusted using the Benjamini-Hochberg FDR correction procedure (NS, not significant; *p < 0.05; **p < 0.01; ***p < 0.001). (**C**) Stacked bar charts showing clinical characteristics of (i) RAI (n = 260) versus non-RAI (n = 173) treated THCA patients or (ii) THCA patients stratified by BRAF-like (n = 272) or RAS-like (n = 119) tumoral genetic signatures. Clinical staging attributes include risk group, tumor staging, lymph node staging and disease staging. P-values derived using Chi-Squared test and adjusted using the Benjamini-Hochberg FDR correction procedure. NS, not significant.

**A**

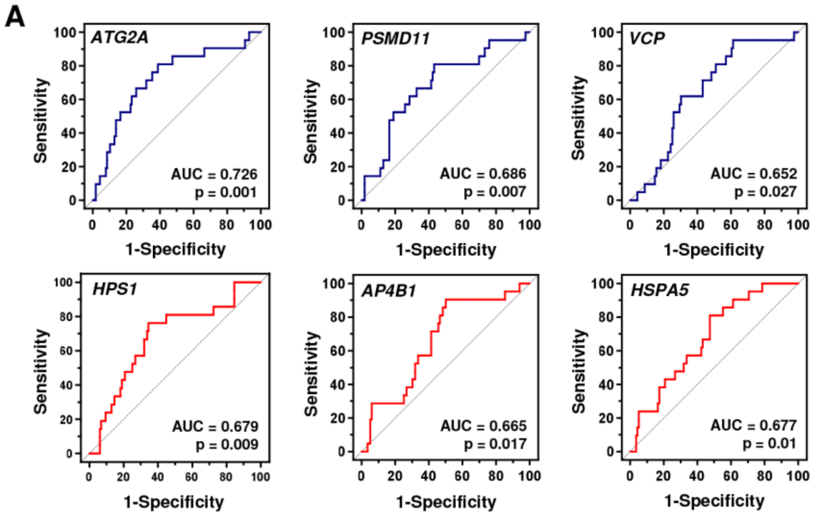


**B**

| Gene | Sensitivity (%) | Specificity (%) | Cut-off value |
|---|---|---|---|
| *AP3D1* | 52.38 | 81.03 | 11.89 |
| *AP4B1* | 90.48 | 50.00 | 8.23 |
| *ATG2A* | 80.95 | 61.21 | 10.10 |
| *ATG9A* | 76.19 | 59.48 | 10.48 |
| *BECN1* | 57.14 | 70.69 | 10.76 |
| *HPS1* | 76.19 | 65.52 | 10.75 |
| *HSPA5* | 80.95 | 52.59 | 14.04 |
| *PSMD2* | 76.19 | 55.17 | 11.76 |
| *PSMD8* | 57.14 | 69.83 | 11.47 |
| *PSMD11* | 80.95 | 56.90 | 9.48 |
| *SEC24* | 76.19 | 61.21 | 11.52 |
| *SQSTM1* | 80.95 | 51.72 | 14.15 |
| *VCP* | 61.90 | 69.83 | 12.77 |

**C** THCA (BRAF-like, RAI treated (n = 137): ROC analysis cut-off values

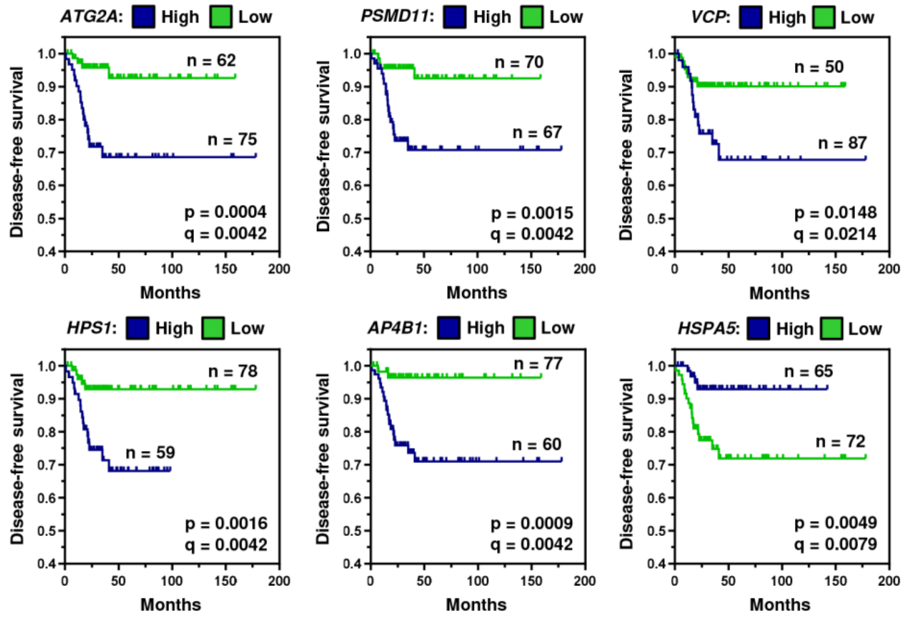


**D** THCA (BRAF-like, RAI-treated, n = 137): Percentile cut-off values

| Gene | THCA: BRAF-like, RAI-treated (n = 137) | | | | | | | | | | | | | | | | | |
|---|---|---|---|---|---|---|---|---|---|---|---|---|---|---|---|---|---|---|
| | HIGH (>66) v LOW (<33) | | | HIGH (>66) V LOW (<66) | | | HIGH (>33) V LOW (<33) | | | HIGH (>75) V LOW (<25) | | | HIGH (>50) V LOW (<50) | | | HIGH (>75) V LOW (<75) | | |
| | p | q | NREC | p | q | NREC | p | q | NREC | p | q | NREC | p | q | NREC | p | q | NREC |
| *AP3D1* | 0.0248 | 0.0465 | 12,4 | 0.0110 | 0.0300 | 12,9 | 0.1232 | 0.1719 | 17,4 | 0.0193 | 0.0380 | 11,3 | 0.1018 | 0.1103 | 14,7 | 0.0022 | 0.0141 | 11,10 |
| *AP4B1* | 0.0288 | 0.0465 | 2,9 | 0.0153 | 0.0300 | 2,19 | 0.3412 | 0.3412 | 12,9 | 0.1372 | 0.1372 | 2,6 | 0.0333 | 0.0481 | 6,15 | 0.7770 | 0.7770 | 2,19 |
| *ATG2A* | 0.0033 | 0.0216 | 14,3 | 0.0004 | 0.0049 | 14,7 | 0.0398 | 0.1719 | 18,3 | 0.0042 | 0.0350 | 11,2 | 0.0020 | 0.0174 | 17,4 | 0.0015 | 0.0141 | 11,10 |
| *ATG9A* | 0.0468 | 0.0554 | 11,4 | 0.0481 | 0.0625 | 11,10 | 0.1216 | 0.1719 | 17,4 | 0.0081 | 0.0350 | 9,1 | 0.0115 | 0.0331 | 16,5 | 0.0734 | 0.1061 | 9,12 |
| *BECN1* | 0.0322 | 0.0465 | 12,4 | 0.0115 | 0.0300 | 12,9 | 0.1455 | 0.1719 | 17,4 | 0.0526 | 0.0622 | 9,3 | 0.0303 | 0.0481 | 15,6 | 0.0376 | 0.0814 | 9,12 |
| *HPS1* | 0.0428 | 0.0554 | 12,4 | 0.0161 | 0.0300 | 12,9 | 0.1844 | 0.1997 | 17,4 | 0.0352 | 0.0572 | 10,3 | 0.0052 | 0.0224 | 17,4 | 0.0098 | 0.0317 | 10,11 |
| *HSPA5* | 0.0120 | 0.0390 | 2,10 | 0.0149 | 0.0300 | 2,19 | 0.1053 | 0.1719 | 11,10 | 0.0057 | 0.0350 | 1,9 | 0.0767 | 0.0906 | 7,14 | 0.0259 | 0.0673 | 1,20 |
| *PSMD2* | 0.0603 | 0.0653 | 11,4 | 0.0701 | 0.0759 | 11,10 | 0.1294 | 0.1719 | 17,4 | 0.0511 | 0.0622 | 9,3 | 0.0127 | 0.0331 | 16,5 | 0.0667 | 0.1061 | 9,12 |
| *PSMD8* | 0.0285 | 0.0465 | 11,3 | 0.0583 | 0.0689 | 11,10 | 0.0532 | 0.1719 | 18,3 | 0.0179 | 0.0380 | 8,1 | 0.1352 | 0.1352 | 14,7 | 0.1736 | 0.2052 | 8,13 |
| *PSMD11* | 0.0173 | 0.0451 | 13,4 | 0.0050 | 0.0300 | 13,8 | 0.1058 | 0.1719 | 17,4 | 0.0204 | 0.0380 | 11,3 | 0.0027 | 0.0174 | 17,4 | 0.0052 | 0.0224 | 11,10 |
| *SEC24C* | 0.1025 | 0.1025 | 10,4 | 0.1871 | 0.1871 | 10,11 | 0.1398 | 0.1719 | 17,4 | 0.1342 | 0.1372 | 9,4 | 0.0171 | 0.0335 | 16,5 | 0.0598 | 0.1061 | 9,12 |
| *SQSTM1* | 0.0108 | 0.0390 | 11,2 | 0.0439 | 0.0625 | 11,10 | 0.0209 | 0.1355 | 19,2 | 0.0181 | 0.0380 | 8,1 | 0.0180 | 0.0335 | 16,5 | 0.1566 | 0.2036 | 8,13 |
| *VCP* | 0.0018 | 0.0216 | 12,1 | 0.0260 | 0.0422 | 12,9 | 0.0037 | 0.0485 | 20,1 | 0.0415 | 0.0599 | 7,1 | 0.0679 | 0.0883 | 15,6 | 0.5690 | 0.6164 | 7,14 |
| Genes (p < 0.05; q < 0.05) | | 9/13 | | | 8/13 | | | 1/13 | | | 7/13 | | | 9/13 | | | 4/13 | |

**Figure S5 (related to Fig. 5D-F). A 13 Proteostasis Gene Panel is Associated with Recurrence in the BRAF-like, RAI-Treated THCA Cohort**

(**A**) Representative ROC curves of 6 proteostasis genes in the BRAF-like, RAI-treated THCA cohort (n = 137). (**B**) Comparison of clinical sensitivity and specificity of 13 proteostasis genes derived by ROC analysis using optimal cut-off expression values ($log_2$ values) in the BRAF-like, RAI-treated THCA cohort (n = 137). (**C**) Representative Kaplan-Meier analysis of DFS for the BRAF-like, RAI-treated THCA cohort stratified on high versus low tumoral expression of indicated core proteostasis genes; log-rank test. Number (n) of patients per expression sub-group (high/low), p-values and q-values are shown. (**D**) Kaplan-Meier analysis of the BRAF-like, RAI-treated THCA cohort stratified on high versus low tumoral expression according to indicated percentile cut-off values; log-rank test. Significance indicated by p- and q-values. $N_{REC}$ = number of recurrent cases in cohort with either high (left) or low (right) tumoral proteostasis expression. Green = FDR < 5%; Orange = greatest level of significance per percentile cut-off group; Yellow = highest percentage of stratified recurrent cases per percentile cut-off group.

**A** THCA (BRAF-like, RAI-treated, n = 137)

13 gene signature (continuous, multi-factor Cox)

| Gene | Multivariate | |
|---|---|---|
| | Coefficient | p-value |
| *AP3D1* | 0.000088 | 0.902 |
| *AP4B1* | 0.002892 | 0.584 |
| *ATG2A* | -0.000932 | 0.447 |
| *ATG9A* | 0.001931 | 0.116 |
| *BECN1* | 0.002862 | 0.110 |
| *HSPA5* | -0.000125 | 0.031 |
| *HPS1* | 0.000045 | 0.969 |
| *PSMD2* | -0.001065 | 0.087 |
| *PSMD8* | 0.000185 | 0.687 |
| *PSMD11* | 0.002498 | 0.440 |
| *SEC24C* | 0.002052 | 0.201 |
| *SQSTM1* | 0.000036 | 0.653 |
| *VCP* | 0.000265 | 0.354 |

**B** THCA (BRAF-like, RAI-treated, n = 137)

| Riskscore | Sensitivity (%) | Specificity (%) | HR (95% CI) | p-value |
|---|---|---|---|---|
| 10.84 | 100 | 18.97 | 26.381 (0.164-4231.231) | NS |
| 12.60 | 95.24 | 69.83 | 35.862 (4.81-267.405) | $4.79x10^{-4}$ |
| 12.73 | 90.48 | 71.55 | 17.794 (4.142-76.445) | $1.09x10^{-4}$ |
| 12.86 | 85.71 | 75.86 | 13.352 (3.930-45.358) | $3.30x10^{-5}$ |
| 13.03 | 80.95 | 80.17 | 11.978 (4.027-35.626) | $8.0x10^{-6}$ |
| 13.13 | 71.43 | 82.76 | 7.977 (3.090-20.589) | $1.8x10^{-5}$ |
| 13.16 | 66.67 | 82.76 | 6.980 (2.812-17.326) | $2.8x10^{-5}$ |
| 13.21 | 61.9 | 83.62 | 6.189 (2.562-14.951) | $5.1x10^{-5}$ |

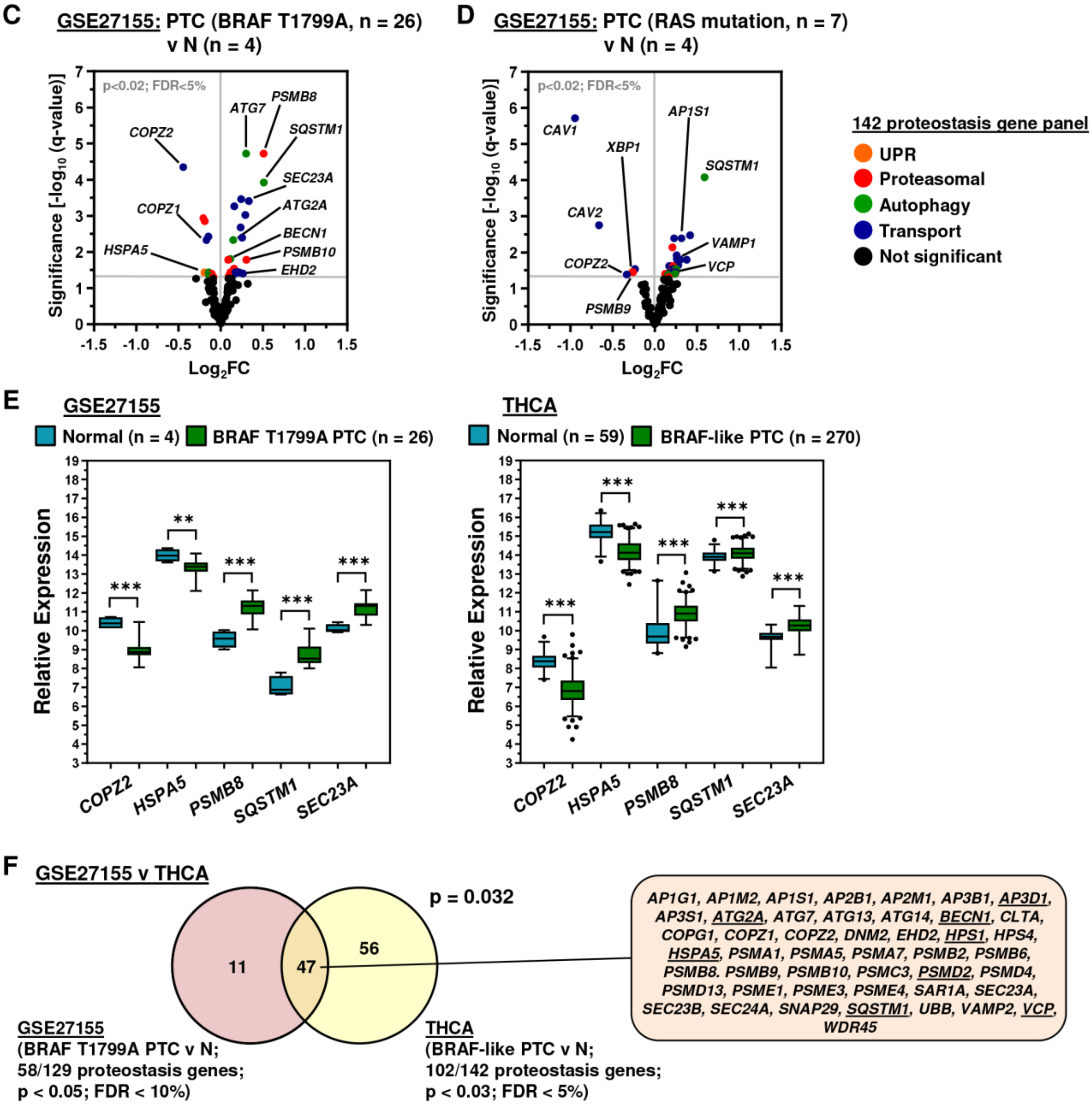

**Figure S6 (related to Fig. 5C, G, H, I). Profiling and Validation of the 13 Proteostasis Gene Riskscore Classifier**

(**A**) Regression coefficients of 13 proteostasis genes in the BRAF-like, RAI-treated THCA cohort (multivariate Cox regression analysis). (**B**) Profile of riskscore cut-off values of the 13 gene proteostasis riskscore classifier along with estimations of sensitivity and specificity (%) for the BRAF-like, RAI-treated THCA cohort (n = 137). Hazard ratios (HR) ± 95% CI using riskscore cut-off values to stratify into high and low risk cohorts are shown (univariate Cox regression analysis). (**C, D**) Volcano plot comparing $\log_2$FC with q-value (-log base 10) for 142 core proteostasis genes in the GSE27155 dataset [BRAF T1799A PTC versus N (C) and RAS mutation PTC versus N (D)]. Gene categories indicated by coloured spots. (**E**) Representative box and whisker plots showing expression ($\log_2$) of 5 core proteostasis genes in the GSE27155 (BRAF T1799A PTC versus normal; left) and THCA (BRAF-like PTC versus normal; right) datasets; P-values determined by Mann-Whitney test and adjusted using the Benjamini-Hochberg FDR correction procedure (**p < 0.01; ***p < 0.001). (**F**) Venn diagram showing significant overlap in the 142 core proteostasis gene panel between the GSE27155 (BRAF T1799A PTC versus normal) and THCA (BRAF-like PTC versus normal) datasets. Genes in the 13 gene proteostasis riskscore classifier are underlined.

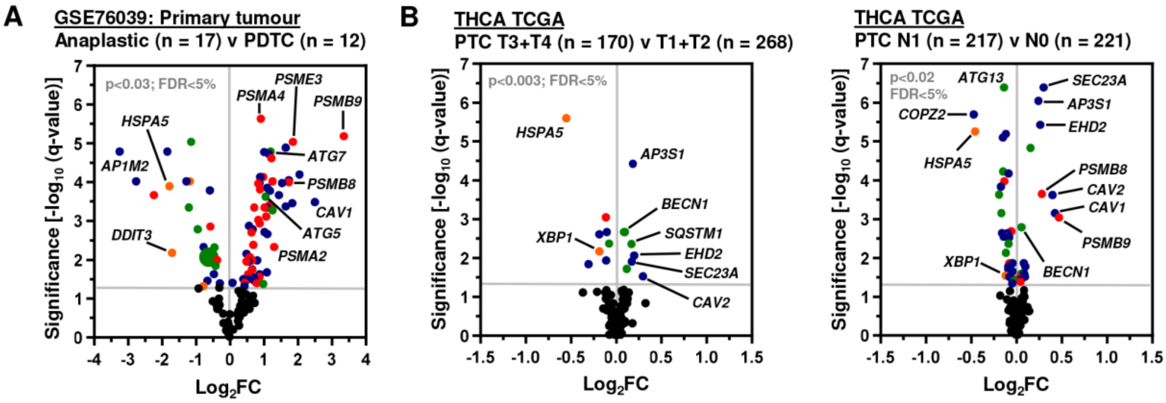


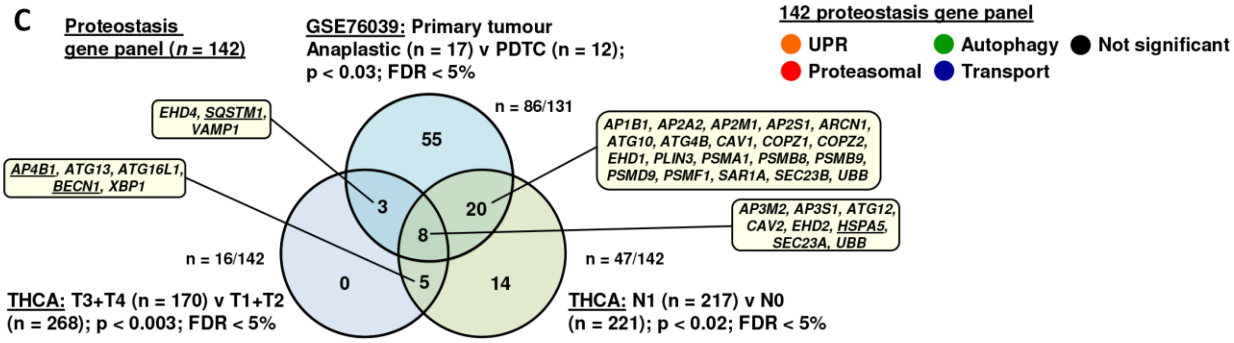


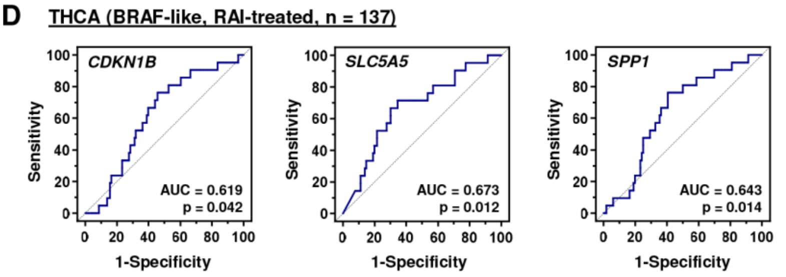


| Gene | AUC | p-value | Cut-off value |
|---|---|---|---|
| *CDKN1A* | 0.557 | NS | 12.89 |
| *CDKN1B* | 0.619 | 0.042 | 10.43 |
| *CCND1* | 0.527 | NS | 13.38 |
| *CDH1* | 0.538 | NS | 14.05 |
| *CEACAM1* | 0.638 | 0.021 | 5.52 |
| *SLC5A5* | 0.673 | 0.012 | 0.34 |
| *SPP1* | 0.643 | 0.014 | 9.61 |

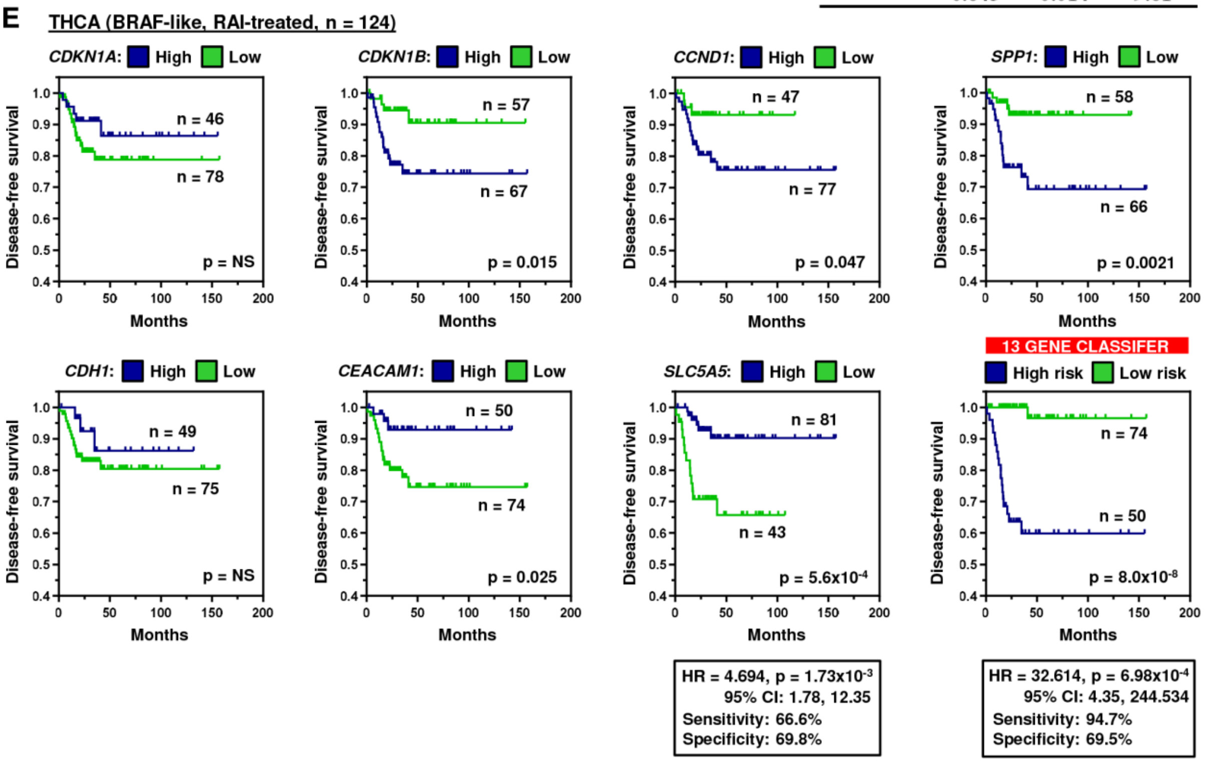

**Figure S7 (related to Fig. 5). Greater Predictive Value of the 13-Gene Riskscore Classifier Compared to a Panel of Molecular Biomarkers**

(**A**) Volcano plot comparing $log_2$FC with q-value (-log base 10) for 142 core proteostasis genes in the GSE276039 dataset [anaplastic thyroid cancer (n = 17) versus PDTC (n = 12)]. (**B**) Volcano plot illustrating $log_2$FC with q-value (-log base 10) for 142 core proteostasis genes in the entire THCA cohort [T3+T4 versus T1+T2 (middle); N1 versus N0 (right)]. Gene categories indicated by coloured spots. (**C**) Venn diagram showing overlap in the 142 core proteostasis gene panel between the GSE276039 (anaplastic thyroid cancer versus PDTC), THCA (T3+T4 versus T1+T2) and THCA (N0 versus N1) datasets. Genes in the 13 gene proteostasis risk score classifier are underlined. (**D**) Representative ROC curves (left) of 3 molecular biomarkers in the BRAF-like, RAI-treated THCA cohort (n = 137). Comparison of ROC analysis (right) using 7 molecular biomarkers in the BRAF-like, RAI-treated THCA cohort (n = 137). AUC, p-values and optimal cut-off expression values ($log_2$ values) are shown. (**E**) Representative Kaplan-Meier analysis of DFS for the BRAF-like, RAI-treated THCA cohort (n = 124) stratified on high versus low tumoral expression of 7 genes reported as biomarkers of thyroid cancer recurrence; log-rank test. Kaplan-Meier analysis using the 13-gene riskscore classifier to stratify patients is included for comparison. Number (n) of patients per expression/risk sub-group (high/low) and p-values are shown.

**Table S1 (related to Fig. 1D, 1E, S1A, S1B). Top 50 Drugs Identified in the Primary High Throughput Screen Using YFP as a Biosensor of Intracellular Iodide**

| Drug | ΔYFP | Cell Viability (%) | Category | Known or putative target(s) |
|---|---|---|---|---|
| Antimycin A | 5.063 | 89.30 | Piscicide | Autophagy |
| Demecarium | 5.008 | 77.22 | Anticholinesterase | N/A |
| Ebastine | 4.524 | 83.37 | Antihistamine | VCP |
| Tioconazole | 4.254 | 61.72 | Antifungal | Autophagy (ATG4B inhibitor) |
| Bromocryptine | 4.199 | 51.41 | Dopamine agonist | Autophagy |
| Vanoxerine | 4.145 | 64.00 | Dopamine reuptake | N/A |
| Dequalinium | 3.757 | 54.66 | Antimicrobial | N/A |
| Articaine | 3.598 | 84.90 | Anesthetic | N/A |
| Astemizole | 3.259 | 9.85 | Antifungal | Lysosomotrophic, VCP |
| Nomegestrol | 3.189 | 99.87 | Progestogen | N/A |
| Eucatropine | 3.087 | 96.65 | Anticholinergic | N/A |
| Lithocholic acid | 3.078 | 77.97 | Detergent | Autophagy, ER stress |
| Tosufloxacin | 2.959 | 76.29 | Antibiotic | N/A |
| Clotrimazole | 2.923 | 89.98 | Antifungal | VCP |
| Rosiglitazone | 2.854 | 96.47 | Hypoglycemic | Autophagy, mTOR |
| Vatalanib | 2.832 | 78.49 | Anticancer | Tyrosine kinase inhibitor (TKI) |
| Pyrvinium | 2.831 | 72.32 | Anthelminthic | Autophagy |
| Phentolamine | 2.830 | 108.85 | α-adrenergic antagonist | N/A |
| Atenolol | 2.808 | 98.52 | Beta blocker | N/A |
| Itopride | 2.760 | 80.40 | Dopamine D2 antagonist | N/A |
| Formoterol | 2.741 | 119.65 | β(2) -agonist | Autophagy, proteasomal |
| Fenofibrate | 2.606 | 103.58 | Lipid-lowering | Autophagy |
| Terfenadine | 2.578 | 83.49 | Antihistamine | VCP |
| Clofilium | 2.534 | 39.77 | Anti-arrhythmia | N/A |
| Rivastigmine | 2.483 | 122.32 | Anticholinesterase | N/A |
| Pravastatin | 2.467 | 101.97 | Lipid-lowering | HMG-CoA reductase |
| Fendiline | 2.412 | 70.95 | Calcium channel blocker | N/A |
| Imatinib | 2.358 | 105.36 | Anticancer | Autophagy, lysosomotrophic, TKI |
| Mifepristone | 2.341 | 93.41 | Antiprogestogenic | Autophagy, unfolded protein response |
| Piperacetazine | 2.321 | 85.25 | Antipsychotic | N/A |
| Methyldopate | 2.311 | 103.17 | Antihypertensive | N/A |
| Hesperidin | 2.270 | 68.98 | Flavanone glycoside | Autophagy, ER stress |
| Rufloxacin | 2.269 | 112.38 | Antibiotic | N/A |
| Nortriptyline | 2.248 | 74.00 | Antidepressant | Autophagy, lysosomotrophic |
| Methotrimeprazine | 2.222 | 91.01 | Phenothiazine | N/A |
| Oxyphenbutazone | 2.219 | 62.83 | Anti-inflammatory | N/A |
| Prednisolone | 2.209 | 92.22 | Anti-inflammatory | N/A |
| Phenformin | 2.165 | 102.43 | Antidiabetic | Autophagy, mTOR |
| Clomipramine | 2.151 | 87.88 | Antidepressant | Autophagy, lysosomotrophic |
| Halofantrine | 2.143 | 105.63 | Antimalarial | Autophagy, lysosomotrophic |
| Sisomicin | 2.122 | 147.72 | Antibiotic | N/A |
| Oxantel | 2.115 | 78.93 | Anthelminthic | N/A |
| Trimethoprim | 2.108 | 97.82 | Antibiotic | N/A |
| Chloroquine | 2.075 | 120.91 | Antimalarial | Autophagy, lysosomotrophic, vesicular transport |
| Propidium | 2.070 | 85.28 | Intercalation | N/A |
| Meclozine | 2.060 | 69.82 | Antihistamine | Histamine H1 antagonist |
| Quinidine | 2.059 | 88.15 | Anti-arrhythmia | Lysosomotrophic |
| Ceforanide | 2.049 | 89.90 | Antibiotic | N/A |
| Clonixin | 2.009 | 120.13 | Anti-inflammatory | N/A |
| Tropisetron | 2.004 | 78.57 | Antiemetic | N/A |

Drugs listed in order of ΔYFP values utilizing TPC-1-NIS cells treated with a 10 μM dose for 24 hr. TPC-1-NIS cell viability (%) after 24 hr drug treatment, drug category and known or putative proteostasis drug targets are shown.

**A** TPC-1-NIS-YFP cells (> 70% cell viability)

| | Drug | AUC | Maximal ΔYFP | Dose (μM) | | Drug | AUC | Maximal ΔYFP | Dose (μM) | | Drug | AUC | Maximal ΔYFP | Dose (μM) |
|---|---|---|---|---|---|---|---|---|---|---|---|---|---|---|
| 1 | Demecarium | 8.02 | 5.06 | 25 | 11 | Alverine | 3.63 | 2.52 | 12.50 | 21 | Levocabastine | 1.15 | 2.10 | 3.13 |
| 2 | Pyrvinium | 6.73 | 8.17 | 0.78 | 12 | Clotrimazole | 3.32 | 2.53 | 0.20 | 22 | Amikacin | 0.65 | 2.29 | 0.20 |
| 3 | Guanabenz | 5.56 | 4.12 | 1.56 | 13 | Phenformin | 3.19 | 3.97 | 50 | 23 | Sertaconazole | 0.38 | 2.44 | 25 |
| 4 | Proparacaine | 5.41 | 3.87 | 0.39 | 14 | Sulconazole | 3.09 | 3.83 | 12.5 | 24 | (R)-(+)Atenolol | 3.16 | 1.87 | 0.78 |
| 5 | Niflumic acid | 4.95 | 2.70 | 1.56 | 15 | Terfenadine | 3.00 | 2.58 | 6.25 | 25 | Azathioprine | 2.93 | 1.92 | 12.50 |
| 6 | Moroxidine | 4.86 | 2.61 | 0.39 | 16 | Halofantrine | 2.28 | 2.06 | 3.13 | 26 | Florfenicol | 2.61 | 1.74 | 0.10 |
| 7 | Nortriptyline | 4.48 | 2.52 | 12.50 | 17 | Hexachlorophene | 2.00 | 3.10 | 50 | 27 | Zimelidine | 2.23 | 1.94 | 6.25 |
| 8 | Chlorhexidine | 4.24 | 5.89 | 6.25 | 18 | Retinoic acid | 1.62 | 2.91 | 0.39 | 28 | Amrinone | 2.11 | 1.91 | 3.13 |
| 9 | Ebastine | 4.19 | 4.52 | 6.25 | 19 | Disulfiram | 1.55 | 2.78 | 3.13 | 29 | Praziquantel | 1.83 | 1.71 | 12.5 |
| 10 | Ethacrynic acid | 3.70 | 2.22 | 0.10 | 20 | Hesperidin | 1.15 | 2.76 | 0.20 | 30 | Articaine | 1.22 | 1.66 | 1.56 |

**B** TPC-1-YFP cells (> 70% cell viability)

| | Drug | AUC | Maximal ΔYFP | Dose (μM) | | Drug | AUC | Maximal ΔYFP | Dose (μM) |
|---|---|---|---|---|---|---|---|---|---|
| 1 | Prednisolone | 4.95 | 3.27 | 0.78 | 14 | Methotrimeprazine | 1.38 | 2.82 | 50 |
| 2 | Piperacetazine | 3.81 | 3.02 | 12.5 | 15 | Guanabenz | 2.40 | 1.94 | 3.13 |
| 3 | Demecarium | 3.50 | 4.51 | 12.5 | 16 | Fenofibrate | 2.25 | 1.59 | 12.5 |
| 4 | Pyrvinium | 3.37 | 4.99 | 0.78 | 17 | Praziquantel | 2.22 | 1.91 | 0.39 |
| 5 | Phenformin | 3.31 | 3.90 | 50 | 18 | Piretanide | 1.41 | 1.86 | 0.1 |
| 6 | Mianserine | 3.30 | 4.34 | 50 | 19 | Niflumic acid | 1.26 | 1.87 | 0.39 |
| 7 | Sertaconazole | 3.05 | 3.57 | 25 | 20 | Ticonazole | 1.15 | 1.54 | 0.20 |
| 8 | Ebastine | 2.94 | 4.56 | 6.25 | 21 | Tridihexethyl | 0.92 | 1.92 | 50 |
| 9 | Dexamethasone | 2.24 | 2.03 | 0.78 | 22 | Retinoic acid | 0.83 | 1.89 | 0.39 |
| 10 | Chlorhexidine | 2.17 | 2.49 | 6.25 | 23 | Clomiphene | 0.82 | 1.81 | 12.5 |
| 11 | Ethacrynic acid | 1.72 | 2.02 | 0.10 | 24 | Clomipramine | 0.78 | 1.67 | 25 |
| 12 | Disulfiram | 1.69 | 2.32 | 0.39 | 25 | Vanoxerine | 0.73 | 1.64 | 6.25 |
| 13 | Fendiline | 1.58 | 2.41 | 12.5 | 26 | Bisacodyl | 0.42 | 1.86 | 50 |

**C** TPC-1-NIS-YFP v TPC-1-YFP cells (> 70% cell viability; i.e adjusted for YFP effects)

| | Drug | ΔAUC | | Drug | ΔAUC | | Drug | ΔAUC |
|---|---|---|---|---|---|---|---|---|
| 1 | Proparacaine | 5.31 | 11 | Sulconazole | 3.09 | 21 | Hesperidin | 1.14 |
| 2 | Moroxidine | 4.57 | 12 | Azathioprine | 2.86 | 22 | Levocabastine | 1.13 |
| 3 | Demecarium | 4.53 | 13 | Florfenicol | 2.61 | 23 | Halofantrine | 1.05 |
| 4 | Nortriptyline | 4.24 | 14 | Terfenadine | 2.53 | 24 | Articaine | 0.95 |
| 5 | Niflumic acid | 3.70 | 15 | Chlorhexidine | 2.07 | 25 | Khellin | 0.92 |
| 6 | Alverine | 3.41 | 16 | Hexachlorophene | 2.00 | 26 | Retinoic acid | 0.79 |
| 7 | Pyrvinium | 3.36 | 17 | Ethacrynic acid | 1.98 | 27 | Quinidine | 0.68 |
| 8 | Clotrimazole | 3.32 | 18 | Zimelidine | 1.81 | 28 | Molindone | 0.66 |
| 9 | Guanabenz | 3.16 | 19 | Amrinone | 1.77 | 29 | Astemizole | 0.60 |
| 10 | (R)-(+)Atenolol | 3.16 | 20 | Ebastine | 1.25 | 30 | Amikacin | 0.54 |

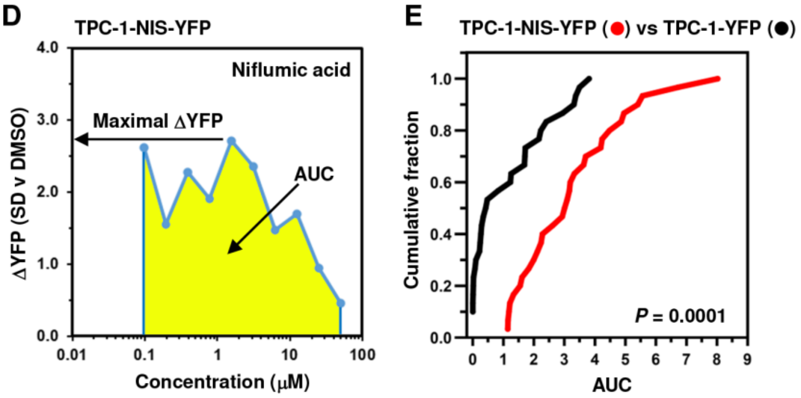

**Table S2 (related to Fig. 1F, S1C). Ranking of Drug Efficacy to Enhance Intracellular Iodide Adjusting for Multiple Doses, YFP-Only Effects, and Cell Viability**

(**A**) Top 30 drugs ranked on area under the curve (AUC) values derived from ΔYFP values of TPC-1-NIS-YFP cells treated with multiple drug doses (0.1-50 μM) and > 70% cell viability. Maximal ΔYFP value and associated dose (μM) for each drug are shown. Drugs with maximal ΔYFP values < 2 were ranked lower. (**B**) Same as (A) but with TPC-1-YFP cells. (**C**) Top 30 drugs ranked on ΔAUC values, i.e., difference in AUC values between drug-treated TPC-1-NIS-YFP and TPC-1-YFP cells. (**D**) Representative dose response YFP-iodide profile highlighting pharmacologic parameters used in analysis, i.e., area under the curve (AUC) and maximal ΔYFP values, in TPC-1-NIS-YFP cells treated with niflumic acid. (**E**) Comparison of drug efficacy in thyroid cells with different NIS levels. Cumulative frequency distribution plot comparing AUC values for top 30 drugs identified to increase intracellular iodide in parental TPC-1-YFP and TPC-1-NIS-YFP cells; P-value determined by the Kolmogorov-Smirnov test.

**Table S3 (related to Fig. 3A, Table 1). Chemical Structure of Putative VCP inhibitors**

| Drug | 2D Structure | 3D Structure | Supporting Evidence |
|---|---|---|---|
| Carebastine | 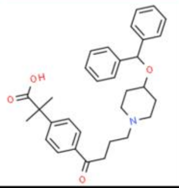 | 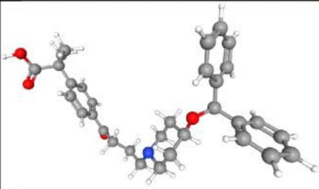 | Drug Combination, RAI Uptake & VCP siRNA assays (this study)<br><br>Active carboxylic acid metabolite of ebastine |
| Fexofenadine | 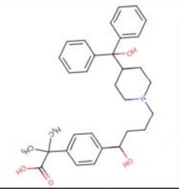 | 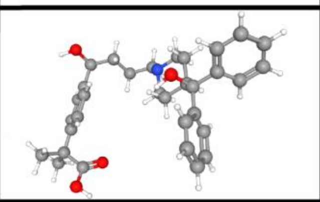 | RAI Uptake assays (this study)<br><br>Active metabolite of terfenadine |
| Terfenadine | 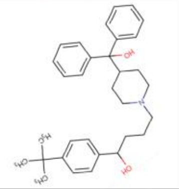 | 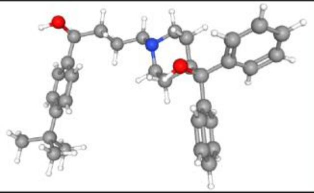 | CMAP (L1000) & RAI Uptake assays (this study)<br><br>CMAP (build 02): MCF7 Breast cells (p = 3.0E-31) (Segura-Cabrera et al., 2017) |
| Disulfiram | 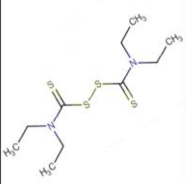 | 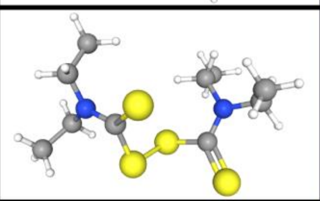 | Drug Combination & RAI Uptake assays (this study)<br>Disulfiram Metabolite (DTC) Binds NPL4 to Disable the VCP-NPL4-UFD1 Pathway (Skrott et al., 2017) |
| Ebastine | 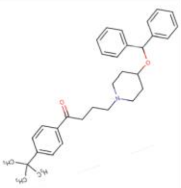 | 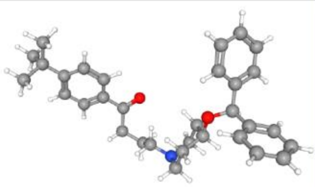 | Drug Combination & RAI Uptake assays (this study)<br><br>Structure-Based Virtual Screening, Cell-Based & Biochemical assays (Segura-Cabrera et al., 2017)<br><br>RAI Uptake, VCP siRNA & Cell Surface Biotinylation (CSB) assays (Fletcher et al., 2020) |
| Astemizole | 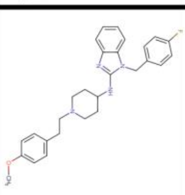 | 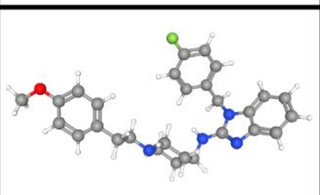 | Drug Combination & RAI Uptake assays (this study)<br><br>CMAP (build 02): MCF7 Breast cells (p = 8.4E-39) Structure-based Virtual Screening, Cell-Based & Biochemical assays (Segura-Cabrera et al., 2017)<br><br>RAI uptake, VCP siRNA & CSB assays (Fletcher et al., 2020) |
| Clotrimazole | 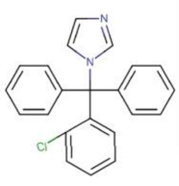 | 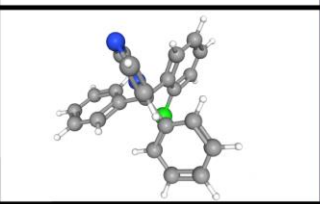 | CMAP (L1000), Drug Combination & RAI Uptake assays (this study)<br><br>CMAP (build 02): MCF7 Breast cells (p = 4.0E-46) Structure-Based Virtual Screening Cell-Based & Biochemical assays (Segura-Cabrera et al., 2017)<br><br>RAI uptake, VCP siRNA & CSB assays (Fletcher et al., 2020) |
| NMS-873 | 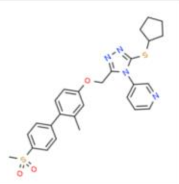 | 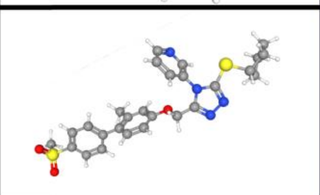 | Well-established VCP inhibitor<br>Used as a control drug for VCP inhibitor studies<br><br>Drug combination & RAI uptake assays (this study) |

2D and 3D images of putative VCP inhibitors are shown, as well as supporting evidence for their ability to target VCP function and enhance NIS function to increase radioiodide uptake. Source: ChemSpider; PubChem.

**Table S4 (related to Fig. 5, S4, S6, S7). Panel of 142 Core Proteostasis Genes**

| Unfolded Protein Response (UPR) | Proteasomal | | | Autophagy | | Transport (protein, vesicular) | | | |
|---|---|---|---|---|---|---|---|---|---|
| *ATF4* | *PSMA1* | *PSMC1* | *PSMD12* | *ATG2A* | *EPG5* | *AP1AR* | *AP3M2* | *COPG1* | *SEC23A* |
| *ATF6* | *PSMA2* | *PSMC2* | *PSMD13* | *ATG2B* | *ERN1* | *AP1B1* | *AP3S1* | *COPG2* | *SEC23B* |
| *DDIT3 (CHOP)* | *PSMA3* | *PSMC3* | *PSMD14* | *ATG3* | *GABARAPL1 (ATG8)* | *AP1G1* | *AP3S2* | *COPZ1* | *SEC24A* |
| *EIF2AK3 (PERK)* | *PSMA4* | *PSMC4* | *PSME1* | *ATG4A* | *SQSTM1 (p62)* | *AP1G2* | *AP4B1* | *COPZ2* | *SEC24B* |
| *HSPA5 (GRP78)* | *PSMA5* | *PSMC5* | *PSME2* | *ATG4B* | *ULK1 (ATG1)* | *AP1M1* | *AP4E1* | *DNM2* | *SEC24C* |
| *XBP1* | *PSMA6* | *PSMC6* | *PSME3* | *ATG4C* | *WDR45* | *AP1M2* | *AP4M1* | *DTNBP1* | *SEC24D* |
| | *PSMA7* | *PSMD1* | *PSME4* | *ATG4D* | *WDR45B* | *AP1S1* | *ARCN1* | *EHD1* | *SEC31A* |
| | *PSMB1* | *PSMD2* | *PSMF1* | *ATG5* | *WIPI1 (ATG18)* | *AP1S2* | *BLOC1S3* | *EHD2* | *SEC31B* |
| | *PSMB2* | *PSMD3* | *UBB* | *ATG7* | *WIPI2 (ATG18B)* | *AP1S3* | *CAV1* | *EHD4* | *SNAP29* |
| | *PSMB3* | *PSMD4* | *UBC* | *ATG9A* | | *AP2A1* | *CAV2* | *HPS1* | *VAMP1* |
| | *PSMB4* | *PSMD5* | *VCP* | *ATG10* | | *AP2A2* | *CLTA* | *HPS3* | *VAMP2* |
| | *PSMB5* | *PSMD6* | | *ATG12* | | *AP2B1* | *CLTB* | *HPS4* | *VAMP3* |
| | *PSMB6* | *PSMD7* | | *ATG13* | | *AP2M1* | *CLTC* | *HPS5* | *VPS13B* |
| | *PSMB7* | *PSMD8* | | *ATG14* | | *AP2S1* | *COPA* | *HPS6* | *VPS33B* |
| | *PSMB8* | *PSMD9* | | *ATG16L1* | | *AP3B1* | *COPB1* | *PLIN3* | |
| | *PSMB9* | *PSMD10* | | *ATG16L2* | | *AP3D1* | *COPB2* | *SAR1A* | |
| | *PSMB10* | *PSMD11* | | *BECN1 (ATG6)* | | *AP3M1* | *COPE* | *SEC13* | |

Functional categories of core proteostasis genes used in study which include: the unfolded protein response (UPR, 6 genes, orange), proteasomal degradation (45 genes, red), autophagy (26 genes, green) and transport (protein/ vesicular, 65 genes, blue).

**Table S5 (related to Fig. 5F). Core Proteostasis Genes are Predictive Indicators of an Increased Risk of Recurrence**

| Gene | Category | High vs Low Expression Groups | | | | | |
|---|---|---|---|---|---|---|---|
| | | Cut-Off Value | B | Hazard Ratio | 95.0% CI | | p-value |
| | | | | | Lower | Upper | |
| *AP3D1* | Transport | 11.89 | 1.311 | 3.709 | 1.574 | 8.741 | 0.003 |
| *AP4B1* | Transport | 8.23 | -1.620 | 0.198 | 0.058 | 0.672 | 0.009 |
| *ATG2A* | Autophagy | 10.10 | 1.747 | 5.736 | 1.928 | 17.063 | 0.002 |
| *ATG9A* | Autophagy | 10.48 | 1.414 | 4.111 | 1.505 | 11.224 | 0.006 |
| *BECN1* | Autophagy | 10.76 | 1.065 | 2.901 | 1.222 | 6.892 | 0.016 |
| *HPS1* | Transport | 10.75 | 1.481 | 4.399 | 1.611 | 12.010 | 0.004 |
| *HSPA5* | UPR | 14.04 | -1.437 | 0.238 | 0.08 | 0.707 | 0.010 |
| *PSMD2* | Proteasomal | 11.76 | 1.221 | 3.390 | 1.241 | 9.256 | 0.017 |
| *PSMD8* | Proteasomal | 11.47 | 0.892 | 2.441 | 1.029 | 5.794 | 0.043 |
| *PSMD11* | Proteasomal | 9.48 | 1.591 | 4.908 | 1.650 | 14.601 | 0.004 |
| *SEC24C* | Transport | 11.52 | 1.414 | 4.112 | 1.506 | 11.228 | 0.006 |
| *SQSTM1* | Autophagy | 14.15 | 1.179 | 3.251 | 1.191 | 8.876 | 0.021 |
| *VCP* | Proteasomal | 12.66 | 2.374 | 10.744 | 1.442 | 80.073 | 0.021 |

Univariate Cox regression analysis in the BRAF-like, RAI-treated THCA cohort stratified using optimal expression cut-off values for 13 proteostasis genes. Cut-off value- $\log_2$ expression value; B- regression coefficient.

**Table S6 (related to Fig. 5H, I). Univariate and Multivariate Analysis of the RAI-Treated (BRAF-like) and Non-RAI Treated THCA Cohort**

### A

| Clinical Variable | n | BRAF-like, RAI-treated (n = 124) | | | | n | Non-RAI treated (n = 151) | | | |
|---|---|---|---|---|---|---|---|---|---|---|
| | | Univariate | | Multivariate | | | Univariate | | Multivariate | |
| | | p-value | HR (95% CI) | p-value | HR (95% CI) | | p-value | HR (95% CI) | p-value | HR (95% CI) |
| **Age, years (±SD)** | | | | | | | | | | |
| < 50 | 79 | 0.072 | 2.288 (0.929-5.634) | 0.403 | 2.054 (0.380-11.105) | 83 | 0.857 | 1.159 (0.234-5.744) | 0.548 | 0.567 (0.089-3.604) |
| > 50 | 45 | | | | | 68 | | | | |
| **Gender** | | | | | | | | | | |
| Male | 39 | 0.994 | 0.996 (0.378-2.623) | 0.807 | 0.877 (0.308-2.500) | 28 | 0.300 | 2.456 (0.449-13.439) | 0.216 | 3.116 (0.516-18.826) |
| Female | 85 | | | | | 123 | | | | |
| **Stage** | | | | | | | | | | |
| I + II | 70 | 0.114 | 2.085 (0.838-5.185) | 0.856 | 0.855 (0.158-4.634) | 125 | 0.244 | 2.744 (0.502-15.004) | 0.677 | 2.104 (0.063-69.879) |
| III + IV | 54 | | | | | 26 | | | | |
| **T stage** | | | | | | | | | | |
| T1 + T2 | 62 | 0.222 | 1.789 (0.704-4.544) | 0.785 | 1.149 (0.424-3.113) | 120 | 0.403 | 2.063 (0.378-11.272) | 0.533 | 2.877 (0.104-79.966) |
| T3 + T4 | 62 | | | | | 31 | | | | |
| **Node stage** | | | | | | | | | | |
| N0 | 34 | 0.449 | 1.531 (0.508-4.617) | 0.109 | 2.587 (0.810-8.264) | 110 | 0.642 | 0.600 (0.070-5.147) | 0.412 | 0.398 (0.044-3.607) |
| N1 | 90 | | | | | 41 | | | | |
| **Risk score (13 gene classifier)** | | | | | | | | | | |
| High | 50 | **$7.0 \times 10^{-4}$** | **32.614 (4.350-244.534)** | **$7.5 \times 10^{-4}$** | **32.781 (4.309-249.369)** | 33 | 0.445 | 0.035 (0.000-190.755) | 0.974 | N/A |
| Low | 74 | | | | | 118 | | | | |

### B

| Clinical Variable | n | BRAF-like, RAI-treated (n = 124) | | | | n | Non-RAI treated (n = 151) | | | |
|---|---|---|---|---|---|---|---|---|---|---|
| | | Univariate | | Multivariate[1] | | | Univariate | | Multivariate[1] | |
| | | p-value | HR (95% CI) | p-value | HR (95% CI) | | p-value | HR (95% CI) | p-value | HR (95% CI) |
| **VCP expression** | | | | | | | | | | |
| High | 84 | **0.031** | **9.166 (1.223-68.679)** | **0.038** | **8.472 (1.122-63.969)** | 51 | 0.940 | 1.067 (0.195-5.830) | 0.872 | 0.867 (0.154-4.876) |
| Low | 40 | | | | | 100 | | | | |

(**A**) n, number; HR, hazard ratio; CI, confidence interval. P-values in bold were less than 0.05 and considered statistically significant. Some patients in the BRAF-like, RAI-treated (n = 13) and non-RAI treated cohorts (n = 16) were not included in univariate and multivariate analysis of the THCA dataset due to missing clinical variables. (**B**) Same as (A) except VCP expression was used instead of the 13-gene risk score classifier in multivariate analysis[1].

**Table S7 (related to Fig. 5H, I). Univariate and Multivariate Analysis of the RAI-Treated and Entire THCA Cohorts**

### A

| Clinical Variable | n | RAI-treated (n = 226) | | | | n | THCA (n = 438) | | | |
|---|---|---|---|---|---|---|---|---|---|---|
| | | Univariate | | Multivariate | | | Univariate | | Multivariate | |
| | | p-value | HR (95% CI) | p-value | HR (95% CI) | | p-value | HR (95% CI) | p-value | HR (95% CI) |
| **Age, years (±SD)** | | | | | | | | | | |
| < 50 | 136 | **0.039** | **2.113 (1.039-4.296)** | 0.352 | 1.771 (0.532-5.897) | 250 | 0.074 | 1.732 (0.949-3.162) | 0.938 | 1.036 (0.430-2.496) |
| > 50 | 90 | | | | | 188 | | | | |
| **Gender** | | | | | | | | | | |
| Male | 74 | 0.678 | 1.169 (0.560-2.44) | 0.537 | 1.271 (0.593-2.725) | 119 | 0.352 | 1.354 (0.715-2.564) | 0.456 | 1.282 (0.668-2.460) |
| Female | 152 | | | | | 319 | | | | |
| **Stage** | | | | | | | | | | |
| I + II | 133 | **0.028** | **2.228 (1.088-4.565)** | 0.719 | 0.795 (0.227-2.779) | 291 | **0.001** | **2.854 (1.561-5.217)** | 0.285 | 1.689 (0.646-4.415) |
| III + IV | 93 | | | | | 147 | | | | |
| **T stage** | | | | | | | | | | |
| T1 + T2 | 116 | **0.010** | **2.758 (1.270-5.99)** | 0.053 | 2.274 (0.990-5.223) | 268 | **0.002** | **2.638 (1.421-4.898)** | 0.243 | 1.516 (0.754-3.049) |
| T3 + T4 | 110 | | | | | 170 | | | | |
| **Node stage** | | | | | | | | | | |
| N0 | 81 | 0.391 | 1.404 (0.646-3.050) | 0.349 | 1.471 (0.656-3.302) | 221 | **0.046** | **1.892 (1.010-3.542)** | 0.413 | 1.32 (0.679-2.568) |
| N1 | 145 | | | | | 217 | | | | |
| **Risk score (13 gene classifier)** | | | | | | | | | | |
| High | 80 | **$8.7 \times 10^{-7}$** | **11.070 (4.248-28.851)** | **$2.0 \times 10^{-6}$** | **10.577 (3.996-28.001)** | 131 | **$3.8 \times 10^{-7}$** | **5.523 (2.801-10.038)** | **$5.0 \times 10^{-6}$** | **4.553 (2.379-8.714)** |
| Low | 146 | | | | | 307 | | | | |

### B

| Clinical Variable | n | RAI-treated (n = 226) | | | | n | THCA (n = 438) | | | |
|---|---|---|---|---|---|---|---|---|---|---|
| | | Univariate | | Multivariate[1] | | | Univariate | | Multivariate[1] | |
| | | p-value | HR (95% CI) | p-value | HR (95% CI) | | p-value | HR (95% CI) | p-value | HR (95% CI) |
| **VCP expression** | | | | | | | | | | |
| High | 78 | 0.102 | 1.801 (0.890-3.645) | 0.172 | 1.654 (0.804-3.404) | 163 | 0.159 | 1.539 (0.845-2.803) | 0.193 | 1.491 (0.817-2.722) |
| Low | 148 | | | | | 275 | | | | |

(**A**) n, number; HR, hazard ratio; CI, confidence interval. P-values in bold were less than 0.05 and considered statistically significant. Some patients in the RAI-treated (n = 31) and entire THCA cohorts (n = 50) were not included in univariate and multivariate analysis of the THCA dataset due to missing clinical variables. (**B**) Same as (A) except VCP expression was used instead of the 13-gene risk score classifier in multivariate analysis[1].
